# Deciphering the Biosynthesis and Physiological Function of 5-Methylated Pyrazinones Produced by Myxobacteria

**DOI:** 10.1021/acscentsci.3c01363

**Published:** 2024-02-07

**Authors:** Le-Le Zhu, Qingyu Yang, De-Gao Wang, Luo Niu, Zhuo Pan, Shengying Li, Yue-Zhong Li, Wei Zhang, Changsheng Wu

**Affiliations:** State Key Laboratory of Microbial Technology, Institute of Microbial Technology, Shandong University, 266237 Qingdao, P.R. China

## Abstract

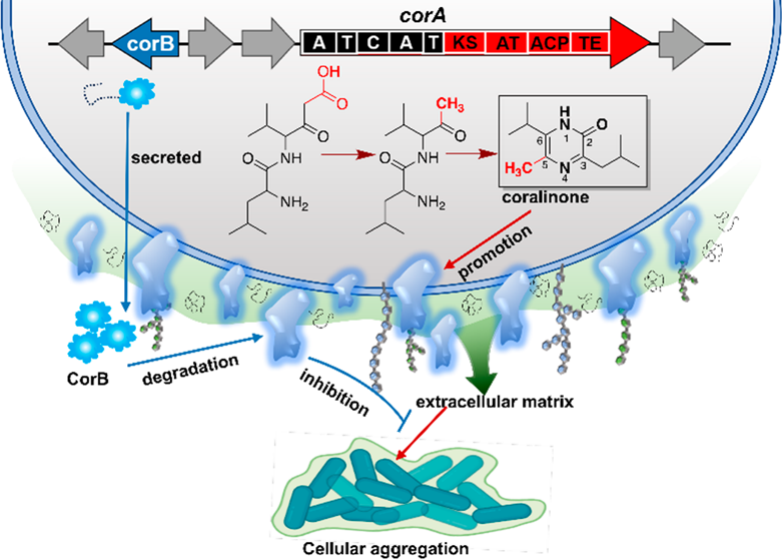

Myxobacteria are a prolific source of secondary metabolites with sheer chemical complexity, intriguing biosynthetic enzymology, and diverse biological activities. In this study, we report the discovery, biosynthesis, biomimetic total synthesis, physiological function, structure–activity relationship, and self-resistance mechanism of the 5-methylated pyrazinone coralinone from a myxobacterium *Corallococcus exiguus* SDU70. A single NRPS/PKS gene *corA* was genetically and biochemically demonstrated to orchestrate coralinone, wherein the integral PKS part is responsible for installing the 5-methyl group. Intriguingly, coralinone exacerbated cellular aggregation of myxobacteria grown in liquid cultures by enhancing the secretion of extracellular matrix, and the 5-methylation is indispensable for the alleged activity. We provided an evolutionary landscape of the *corA*-associated biosynthetic gene clusters (BGCs) distributed in the myxobacterial realm, revealing the divergent evolution for the diversity-oriented biosynthesis of 5-alkyated pyrazinones. This phylogenetic contextualization provoked us to identify *corB* located in the proximity of *corA* as a self-resistance gene. CorB was experimentally verified to be a protease that hydrolyzes extracellular proteins to antagonize the agglutination-inducing effect of coralinone. Overall, we anticipate these findings will provide new insights into the chemical ecology of myxobacteria and lay foundations for the maximal excavation of these largely underexplored resources.

## Abstract 2

*corA* autonomously codes for 5-methylated pyrazinone that promotes secretion of extracellular matrix to induce agglutination of myxobacteria, and *corB* encoding a protease is the self-resistance gene.

## Introduction

Myxobacteria are Gram-negative *Myxococotta* characterized by unique morphological characteristics, complex life cycles, predation, gliding, multicellular fruiting body formation, and large genome size.^1^ This branch of life domain is particularly fascinating because they are well-renowned for producing a wealth of bioactive secondary metabolites endowed with intricated architectures, extraordinary bioactivities, and exquisite mode of action.^2^ According to our recently compiled MyxoDB database,^3^ most of the scaffolds are exclusively found in myxobacteria, underpinning the importance of myxobacteria in terms of drug development. Furthermore, the advent of low-cost genome sequencing has revealed a far greater biosynthetic potential than what we have characterized, ensuring that myxobacteria stand out in the forefront of microbial natural products’ (NPs) research.^4,5^

**Figure 1:**
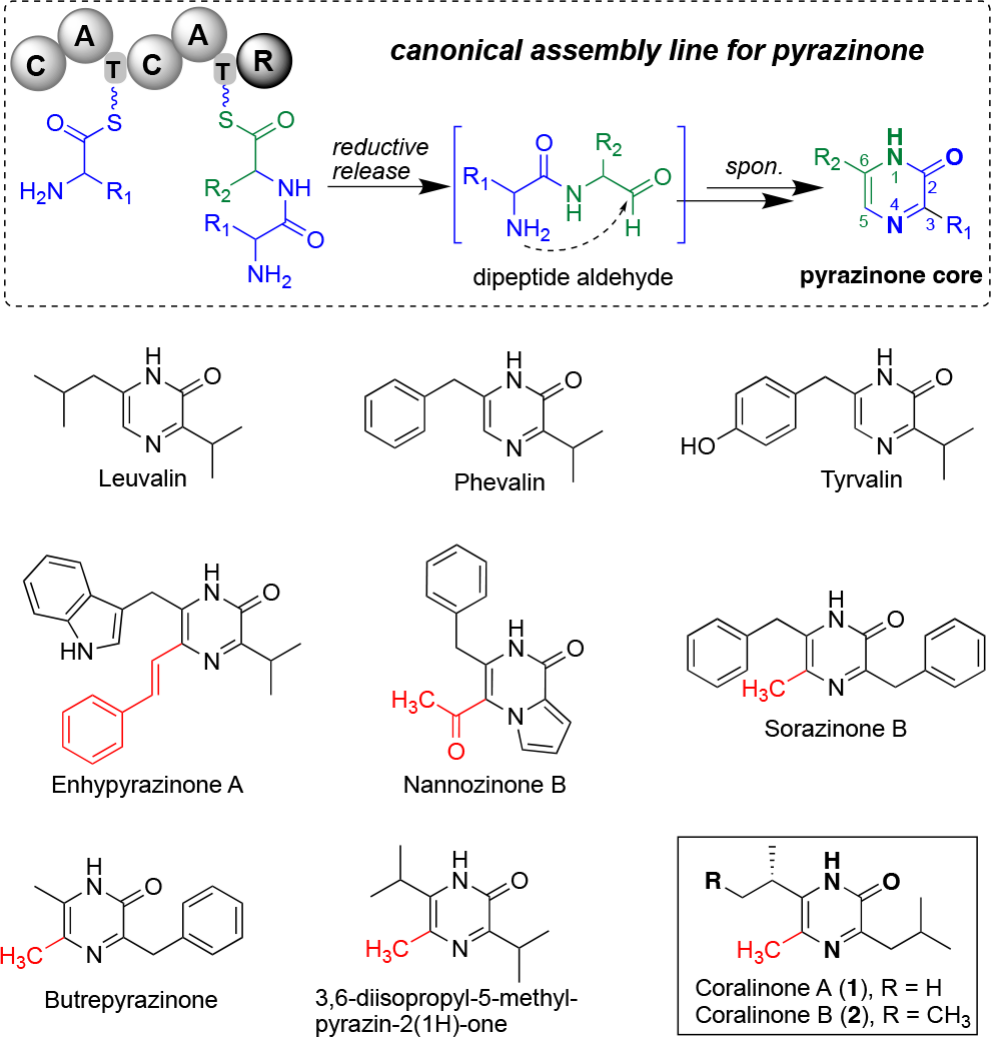
Natural products endowed with pyrazinone nuclei. Typically, the pyrazinone core is assembled by multidomain NRPS enzyme featuring domain organization of C-A-T-C-A-T-R. The substituents at C-5 of the exemplified pyrazinone derivatives are highlighted in red. The coralinones encrypted by *corA* (Figure 2) in *Corallococcus exiguus* SDU70 were previously undescribed *tri*-alkylated pyrazinones. Abbreviations: A, adenylation domain; C, condensation domain; T, thiolation domain; R, reductase domain.

Among all the known myxobacterial NPs, a small structural family is pyrazinone, a nonaromatic heterocyclic ring with one ketonized carbon and two *para*-situated nitrogen atoms (Figure 1). The representative examples are nannozinones and sorazinones discovered from *Nannocystis pusilla* MNa10913,^6^ as well as enhypyrazinone A from the marine-derived *Enhygromyxa* sp. WMMC2695.^7^ Typically, the biosynthesis of pyrazinones in microbes are derived from condensation of two molecules of amino acids through a multidomain nonribosomal peptide synthetase (NRPS) assembly line. The terminal R (reductase) domain releases the tethered dipeptide as a reactive aldehyde. The nucleophilic attack of the aldehyde by the α-amine forms a cyclic imine, followed by oxidation to afford pyrazinone core.^8−11^ Therefore, it is understandable that C-5 of the pyrazinone ring is mostly unsubstituted, as seen in the naturally occurring pyrazinones like phevalin,^12^ tyrvalin, and leuvalin.^8,11^ Intriguingly, there are also a considerable number of pyrazinone compounds bearing differential substituents on C-5 of the pyrazinone skeleton, including but not limited to cinnamoyl (enhypyrazinone),^7^ acetyl (nannozinone B),^6^ and methyl (sorazinone B,^6^ butrepyrazinone^13^), whereas the precise enzymatic machineries for these periphery moieties remained enigmatic.

Pyrazinones are found to be widely distributed in different domain of microorganisms such as fungi,^14^ actinomycetes,^15−17^ staphylococci,^8,11^ and sponges,^18,19^ which implies an important biological and ecological significance. Interestingly, pyrazinone and related chemical scaffolds are oftentimes employed by microbes as signaling molecules rather than chemical weapons to regulate various physiological processes. For example, pyrazinones functionate as quorum sensing regulator to control biofilm formation of *Vibrio cholerae*,^20^ or involve in the pathogenesis of enterohemorrhagic *Escherichia coli*;^21^ phevalin regulates virulence of *Staphylococcus aureus*.^8^ By contrast, the biological and ecological role of pyrazinones for myxobacteria has never been described.

Here, we report the discovery of coralinones, a family of 5-methylated pyrazinones from myxobacterium *Corallococcus exiguus* SDU70. Combining bioinformatics analysis, genetic disruption, and heterologous expression, we identified an NRPS/PKS gene (*corA*) that autonomously codes for coralinones. Further by biochemical characterization and biomimetic total synthesis, we explicitly elaborated the assembly mechanism of coralinones, especially unravelling the enzymology responsible for 5-methylation of the pyrazinone backbone. More remarkably, we decrypted the biological function of coralinones that act as morphogens to modulate the cellular aggregation of *C. exiguus* SDU70 and the model myxobacterium *Myxococcus xanthus* DK1622 grown in liquid cultures. Interestingly, *corB* located in the vicinity of *corA* functions as a self-resistance gene. This study advances our understanding of chemical ecology and/or biology of myxobacteria and inspires new perspectives to excavate this tremendous treasure trove.

## Results

### Discovery of Coralinone from *C. exiguus* SDU70

As a part of our continuing efforts to find drug leads from myxobacteria, we were fascinated with a group of secondary metabolites produced by the strain *C. exiguus* SDU70 during preliminary screening, which gave the characteristic UV absorption spectrum for pyrazinone in the HPLC-DAD profiling (Figure S1). A scaled-up fermentation (34 L) of SDU70 was performed. The repeated chromatography by tracking the sought-after UV peaks led to the purification of compounds **1** and **2**. The structure elucidation was done by a combination of 2D NMR and high-resolution MS analysis (see Supporting Information). As a consequence, the isolated compounds were identified as previously undescribed *tri*-alkylated pyrazinones differing in the isopropyl or isobutyl substituent at C-6 (Figure 1), and they were dubbed as coralinones A (**1**) and B (**2**), respectively. The absolute configuration of C-11 in **2** was determined to be *S* on the basis of X-ray diffraction analysis (Figure S2), concordant with the biosynthetic origin of l-isoleucine (see below). Remarkably, coralinones featured a methyl at C-5 of pyrazinone core further expanding the diversity and complexity of this structural family.

### Genetic and Biochemical Characterization of Biosynthesis Pathway of Coralinone

Retrobiosynthetic analysis and isotope labeling experiments unambiguously confirmed that l-leucine *d*_3_ and l-isoleucine *d*_10_ were the biosynthetic precursors (Figure S3). When SDU70 was fed with ^13^C-labeled S-adenosylmethionine (SAM), no isotopic coralinones were accumulated, excluding the possibility that 5-CH_3_ is an outcome of putative *C*-methyltransferase. As Motoyama and co-workers have shown that aldehydes could participate in the alkylation of pyrazines,^22^ we conducted an additional isotope experiment using [1-^13^C]-labeled formaldehyde, but still no incorporation was observed in the LC-MS analysis.

**Figure 2:**
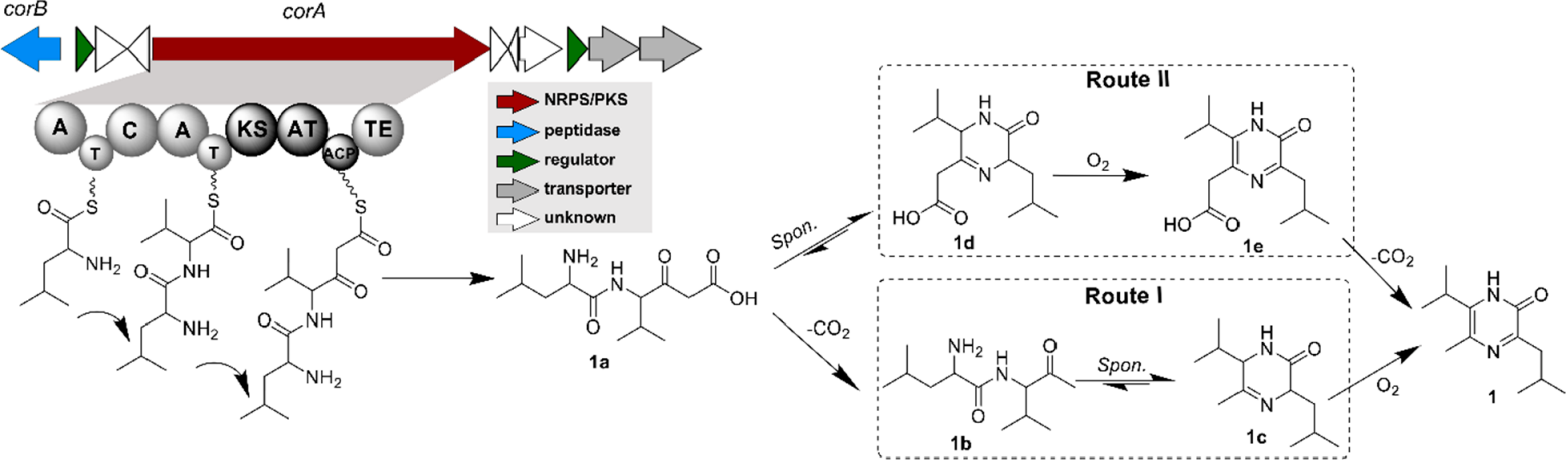
Biosynthetic pathway of coralinone. For the detailed annotation of *cor* gene cluster, see Table S1.

SDU70 was genome sequenced and then subjected to antiSMASH analysis,^23^ which led to the identification of 44 biosynthetic gene clusters (BGCs). We did not find any NRPS BGC encoding the multidomain organization of C-A-T-C-A-T-R (Figure 1) that canonically engages in the biosynthesis of pyrazinone backbone.^8−11^ Instead, a hybrid NRPS/PKS BGC namely *cor* caught our attention because the encoded domain architecture of A-T-C-A-T-KS-AT-ACP-TE was predictably attainable for a peptidic product containing two amino acids, one of the prerequisites for pyrazinone formation. This allowed us to propose a biosynthetic model for coralinones: the textbook modular NRPS/PKS assembly line warrants a dipeptidyl β-keto acid (**1a**) by CorA, which subsequently undergoes spontaneous domino reactions, including β-keto decarboxylation, intramolecular cyclization, and oxidation to generate 5-methylated pyrazinones (Figure 2). The divergence in the sequential reactivity order of decarboxylation and cyclization results in two possible routes (Route I versus II), since both reactions happen spontaneously. However, we postulated that Route I is more favorable, because β-keto acid loses carbon dioxide quite easily, where the immediate product will be a resonant stabilized enolate anion followed by tautomerism into ketone.^24^

To experimentally corroborate the proposed biosynthetic pathway, *corA* was cloned into the custom expression vector pET28a and expressed in *Escherichia coli* BAP1, a strain engineered with *sfp* gene that encodes a promiscuous phosphopantetheinyl transferase for the heterologous priming of T and ACP domains of both NRPSs and PKSs.^25^ The metabolites produced by *E. coli* harboring pET28a-*corA* and pET28a empty vector were extracted in parallel by ethyl acetate and then analyzed by HPLC-DAD (280 nm). HPLC-DAD profiling of pET28a-*corA* presented clearly distinguishable peaks for **1** and **2**. We also carried out *in vitro* biochemical reconstruction of the assembly line. CorA (∼310 kDa) was expressed as a full-length *holo*-protein containing an *N*-terminal His_6_ tag, and then purified as homogeneous from *E. coli* BAP1 (Figure S4).

**Figure 3:**
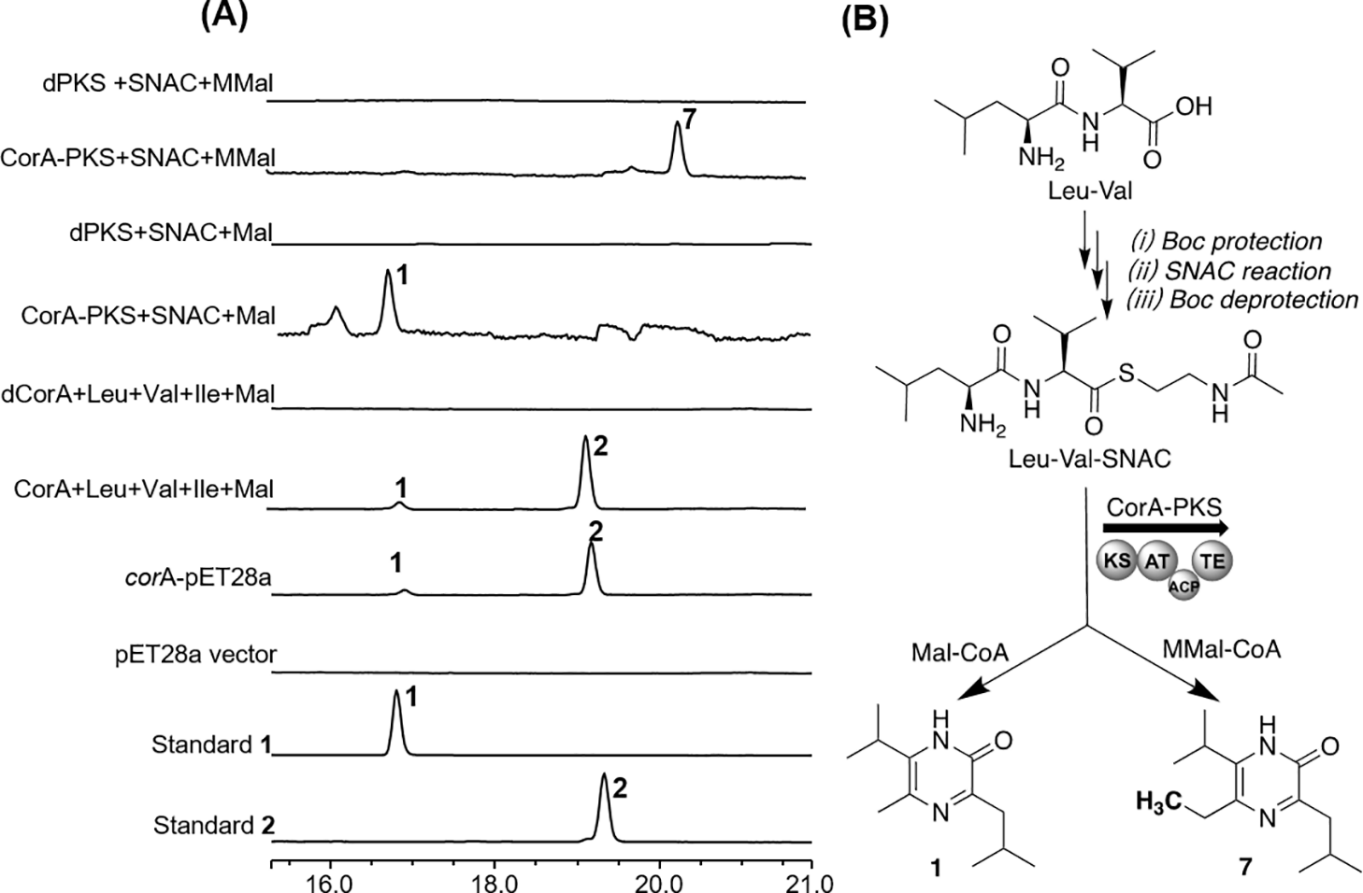
The gene *corA* autonomously codes for coralinones. (A) Demonstration of coralinones production by the single *corA* gene by *in vivo* heterologous expression and *in vitro* biochemical reconstitution. HPLC-DAD analysis was detected at 280 nm. (B) Scheme for the chemoenzymatic synthesis of 5-alkylated pyrazinones **1** and **7** using the truncated enzyme CorA-PKS. Abbreviations: dPKS, denatured CorA-PKS; dCorA, denatured CorA; SNAC, Leu-Val-SNAC; Mal-CoA, malonyl-CoA; MMal-CoA, methylmalonyl-CoA. Each biochemical experiment was done in triplicate.

The catalytic activity of the SFP-activated *holo*-CorA was assessed in the presence of substrates l-Leu, l-Ile, and l-Val alongside Mal-CoA, under the control of boiling-denatured CorA (dCorA). Subsequent HPLC-DAD analysis of the enzymatic reaction confirmed the production of **1** and **2**, whereby the substrate preference of CorA was in general accordance with the *in vivo* results of *E. coli* (Figure 3A). Notably, the inconsistency in the relative abundance of **1** and **2** given by the recombinant *E. coli* BAP1 and native *C. exiguus* SDU70 (Figure S1) implicated that the substrate selectivity of CorA and/or precursor supply is different in these two hosts. To unequivocally correlate 5-methylation with the PKS section of *corA*, we individually expressed the continuous DNA sequence encoding the multidomain KS-AT-ACP-TE in *E. coli* BAP1. The truncated CorA enzyme (hereinafter referred to as CorA-PKS) not surprisingly converted the chemically synthesized dipeptide Leu-Val-SNAC (see Supporting Information) into **1** in the presence of Mal-CoA (Figure 3B).

In consideration that substrate flexibility of A domains seems an intrinsic peculiarity of NRPS assembly line related to pyrazinone biosynthesis,^9^ we explored expanding the chemical repertoire of 5-methylated pyrazinones by virtue of combinational synthesis using *holo*-CorA. In the presence of Mal-CoA, Leu, Ile, or Val were individually paired with the other 19 proteinogenic amino acids, respectively. HPLC-DAD profiling of the 57 different pairwise combination confirmed Leu-Val, Leu-Ile, Met-Ile, Met-Val, Phe-Ile, and Phe-Val produced 5-methylated pyrazinones **1**–**6** (Figure S5), as established by the LC-MS measurements and characteristic UV absorption spectra (Figure S6), all of which were previously undescribed compounds. As the second A domain in CorA is able to accept Ile and Val, the production of **1**–**6** implied that the first A domain is promiscuous to tolerate Leu, Met, and Phe. According to the relative yield of **1**–**6** (Figure S7), we tentatively concluded that the substrate preference for the first A domain is Leu > Met > Phe, while that for the second A domain is Ile > Val. Subsequently, the substrates preference of the first A domain was unambiguously corroborated by the ATP–PPi exchange assay^26^ (Figure S7), in agreement with the pairwise combination assay. Significantly, the AT domain also exhibits substrate flexibility, because an additional new compound 5-ethylated pyrazinone (**7**) was generated when methylmalonyl-CoA (MMal-CoA) instead of Mal-CoA was incubated with CorA-PKS (Figure 3B). Altogether, the *in vivo* and *in vitro* experiments unambiguously corroborated that *corA* autonomously synthesizes coralinones, whereby the PKS moiety is responsible for the formation of 5-methylation.

### Coralinone Exacerbates Cellular Aggregation of *C. exiguus* SDU70 and *M. xanthus* DK1622

**Figure 4:**
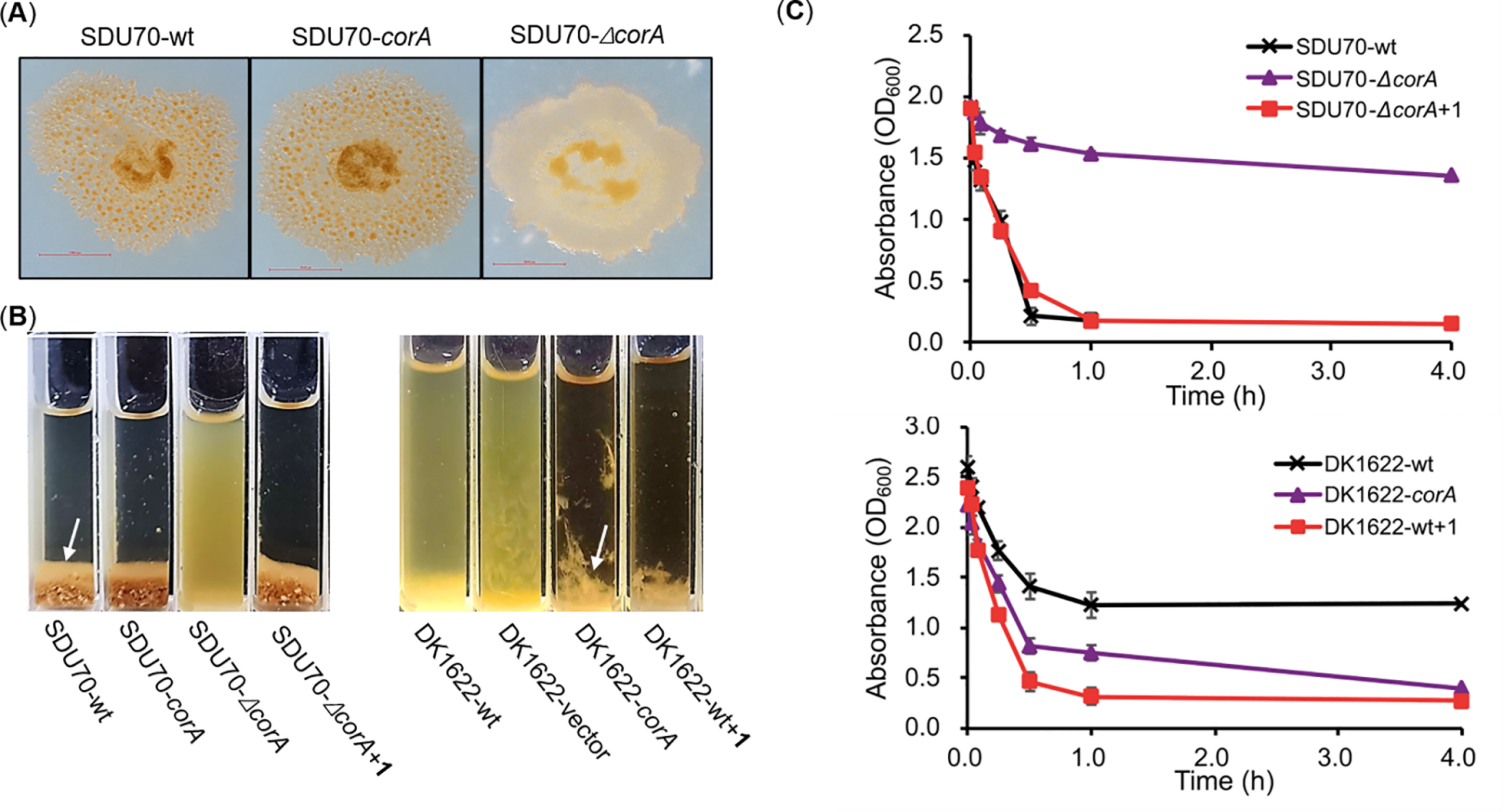
Coralinone caused the flocculation of *C. exiguus* SDU70 and *M. xanthus* DK1622 grown in liquid culture. (A) Comparison of colony morphology of SDU70-wt, SDU70-*corA* and SDU70*-ΔcorA*. Photos were taken after 7 days of growth on CTT agar plates. The colony of SDU70*-ΔcorA* was glossy and defective in fruiting body. (B) and (C) Agglutination assays of SDU70 and DK1622 strains. The photos were taken after samples were transferred into cuvettes and left static for 15 min. Some strains flocculated strongly, so that almost all cells clumped together and sinked to the bottom of the cuvettes, while others showed virtually no flocculation, leaving most cells in suspension. The white arrows denote the flocs of SDU70-wt and DK1622-*corA*. Decreasing absorbance represents the agglutination. Each point in the agglutination assay curve is the average of three independent experiments.

To understand the biological function(s) of the 5-methylated pyrazinones produced by myxobacteria, we assayed **1** and **2** against the Gram-negative bacterium *Acinetobacter baumannii*, Gram-positive bacterium *Staphylococcus aureus*, and the fungus *Candida albicans*. No growth inhibition was observed during agar diffusion assay when applied with 50 μg/disc, consistent with previous investigation by Magarvey and co-workers who demonstrated 5-unsubstituted pyrazinones are not antibiotics.^8^ Neither **1** nor **2** showed any significant antiproliferative activities against four human cell lines (Table S2). These intrigued us to surmise that coralinones might have a distinct activity and play an important physiological role toward SDU70. Therefore, *corA* was constitutively overexpressed or insertion inactivated in SDU70, respectively. The fidelity of genotypes of the resultant mutants SDU70-*corA* or SDU70*-ΔcorA* were confirmed by Sanger sequencing and metabolic analysis (Figure S8). The mutants obtained on solid agar plates were examined for growth development. Interestingly, while little morphogenetic difference was found between SDU70 wild type (SDU70-wt) and overexpression mutant SDU70-*corA*, defect in the fruiting body of null mutant SDU70-*ΔcorA* was conspicuous (Figure 4A). As the fruiting body is closely associated with aggregation of myxobacterial cells,^27,28^ we were intrigued to monitor the agglutination of the alleged three strains in the shaken liquid-grown cultures. The pellet morphogenesis of SDU70-*ΔcorA* was significantly impeded in comparison with SDU70-wt and SDU70-*corA*, which could be restored by the chemical complementation of **1** at 5 mg/L (Figure 4B), a concentration mirroring the total yield level (∼1.5 mg/L) of coralinones given by SDU70-wt (Figure S1). We also tested if coralinones could induce agglutination of model myxobacterium *M.* DK1622. The gene *corA* was expressed in DK1622, wherein the production of **1** and **2** were confirmed by HPLC-DAD analysis (Figure S8). Consistent with the results obtained from SDU70, the dispersed morphology of wild type DK1622 (DK1622-wt) was apparently disrupted. Likewise, exogenous supplementation of 5 mg/L **1** to DK1622-wt also led to flocculation (Figure 4B). In addition, **1** caused the flocculation of SDU70-*ΔcorA* and DK1622-wt in a dose-dependent manner, whereby 2 mg/L started to be active, and 5 mg/L caused severe flocculation (Figure S9). Taken together, given the results obtained from both SDU70 and DK1622, we concluded that coralinones serve as small-molecule morphogens for coordinating cellular aggregation of myxobacteria.

### Biomimetic Total Synthesis of Coralinone for Structure–Activity Relationship Study

**Figure 5:**
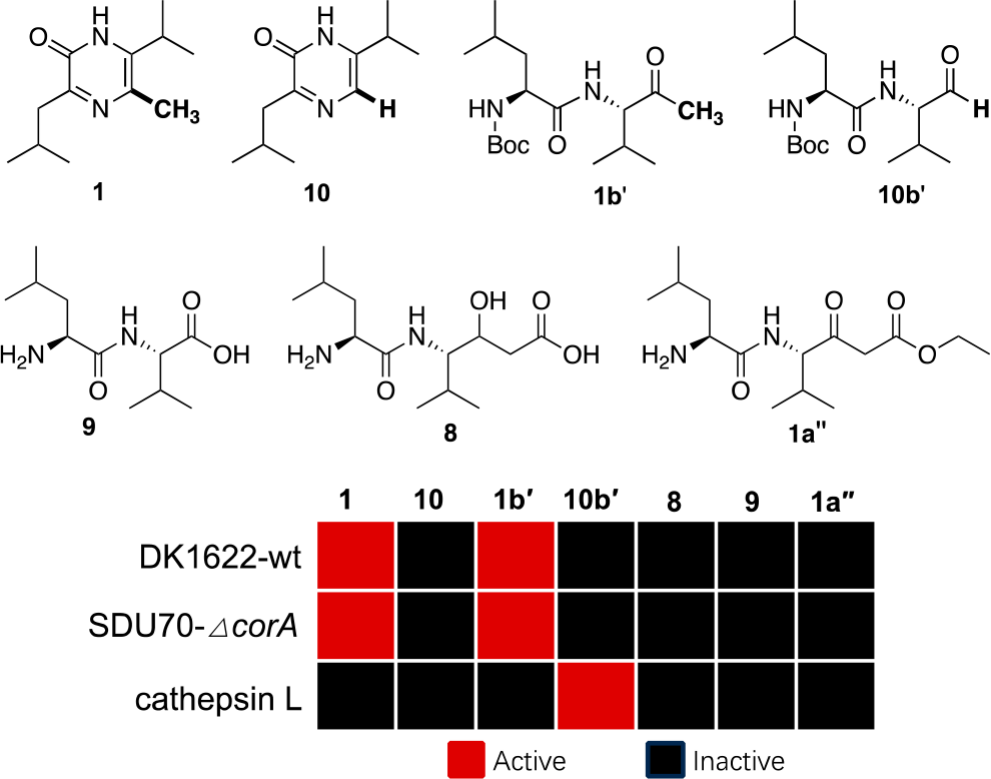
Structure–activity relationship study. The displayed are biosynthetically related compounds in relation to 5-unsubstituted and 5-methylated pyrazinones, for which the activities of protease inhibition and agglutination-induction were tested. For protease inhibitory activity, the cutoff of IC_50_ > 500 μM was arbitrarily deemed as “inactive”. Source files for comparison of agglutination induced by tested compounds can be found in Figure S10. Each experiment was performed in triplicate.

The Fischbach group has revealed that the NRPS enzyme with the domain organization of C-A-T-C-A-T-R that specifies 5-unsubstituted pyrazinone (Figure 1) is associated with protease inhibition, wherein the biosynthetic intermediate dipeptide aldehyde (in Boc-protected form) rather than the mature product is efficacious.^11,29^ In consideration of the close resemblance between 5-unsubstituted and 5-methylated pyrazinones in terms of biosynthetic logic and the chemical skeleton, we initially posited that the dipeptide ketone Leu-Val-CH_3_ (**1b**) might also have protease inhibitory activity. We thereby developed a strategy for the concise synthesis of 5-methylated pyrazinone coralinone A (**1**) and a panel of biosynthetically related compounds (**1b′**, **1a′′**, **8**, **9**) by emulating its biosynthetic routes (see Supporting Information). By contrast, the synthesis of 5-unsubstituted pyrazinone **10** and its biosynthetic intermediate Leu-Val-H (**10b′**) in a Boc-protected form were predicated upon the previously published method.^29^ All the compounds were assayed for the inhibition activity against protease cathepsin L. The Boc-protected dipeptide aldehyde **10b′** exhibited inhibition activity with IC_50_ value of 0.23 μM, but none of the other tested compounds showed efficacy even up to 500 μM. On the other hand, these compounds were also tested for their ability to induce agglutination of SDU70*-ΔcorA* and DK1622-wt, and it was **1** and **1b′** that were effective (Figure 5 and Figure S10). We suspected that SDU70-*ΔcorA* and/or DK1622-wt might serve as biocatalysts for the deprotection of Boc group in **1b′** to release the *bona fide* biosynthetic intermediate **1b**, followed by spontaneous reactions to generate the genuine effector **1**. However, this possibility was subsequently ruled out because **1b′** was found to be recalcitrant to biotransformation (Figure S11). **1b′** also induced the agglutination of SDU70-*ΔcorA* and DK1622-wt in a dose-dependent manner and seemingly shared an equivalent efficacy with **1** (Figure S9).

The structure–activity relationship (SAR) study enabled us to draw the conclusions: (1) both the mature product and biosynthetic intermediate of *corA* pathway functionate as small-molecule morphogens for myxobacteria; (2) 5-methylation substantially influence the bioactivities of the pyrazinone backbone; (3) the domain arrangement of A-T-C-A-T-KS-AT-ACP-TE of *corA* is exquisitely evolved in myxobacteria, whereby terminal free methyl derived from the PKS moiety is indispensable for modulating the morphogenesis of myxobacteria.

### Coralinone Stimulates the Secretion of Extracellular Matrix

**Figure 6:**
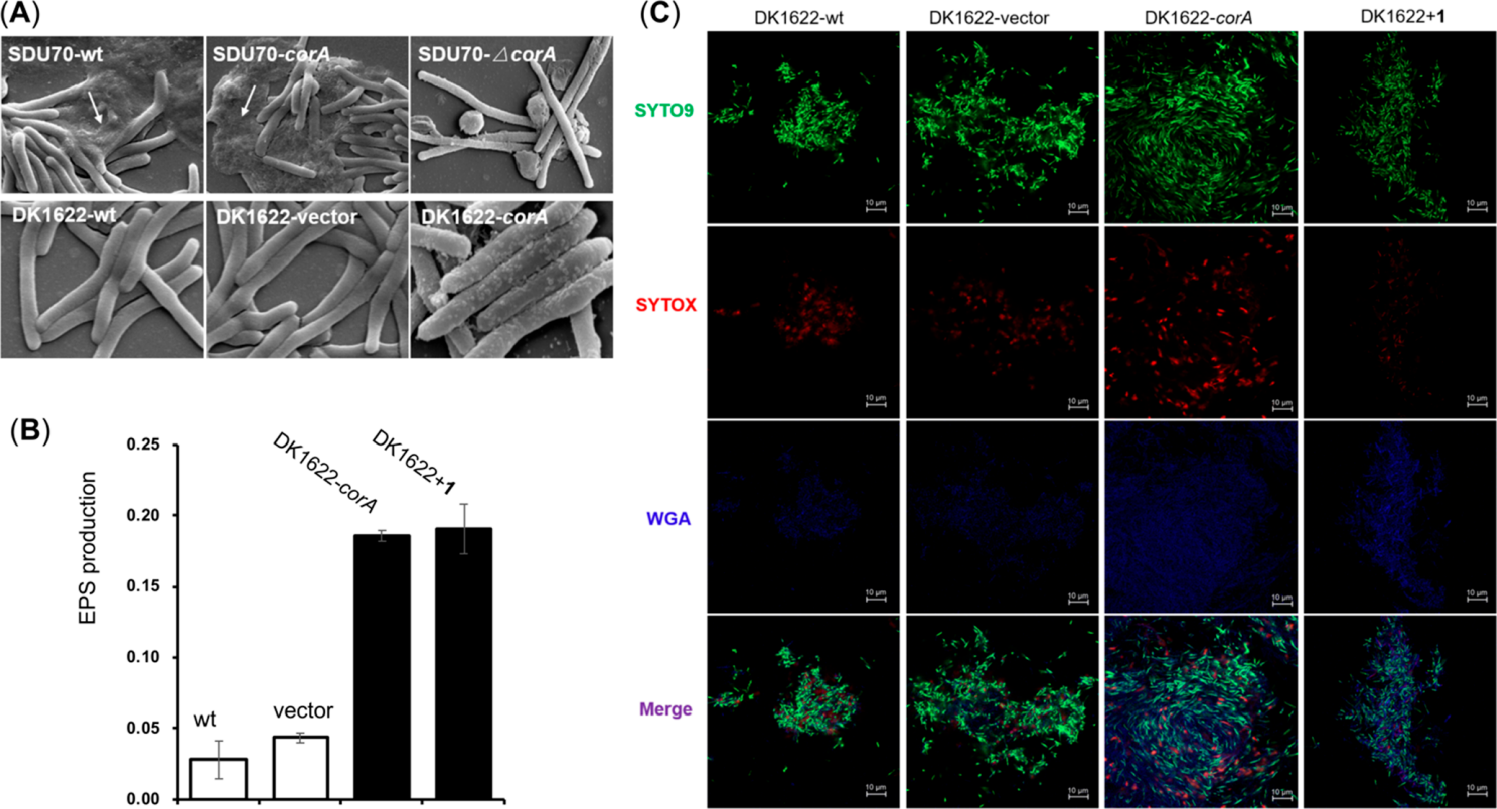
Coralinone promotes extracellular matrix production. (A) SEM imaging of flocculent and nonflocculent strains of DK1622 and SDU70. The white arrows highlight the ECM that are absent in the nonflocculent strains. (B) Comparison of the production of EPS given by DK1622 strains. Error bars correspond to standard deviation. (C) Examination of cells and EPS in biomass derived from different DK1622 strains using confocal laser scanning microscopy (CLSM). Nucleic acids inside cells with compromised membranes were revealed by SYTOX orange labeling, while viable cells with intact membranes were stained by SYTO9. WGA were specific for EPS. The bottom panel (Merge) are the overlay images of SYTO9 (green), SYTOX orange (red), and Alexa 350-WGA (blue) signals. The obvious blue color in the Merge photos of DK1622-*corA* and DK1622+**1** demonstrated a higher production of EPS.

The unique physiological role of coralinone intrigued our interest in investigating the underlying mechanism. It is known that bacterial clumps and/or biofilm systems are always exacerbated by the extracellular matrix (ECM) that normally consists of extracellular polysaccharides (EPS), proteins, and extracellular DNA (eDNA), among other components.^30^ Especially, the aggregative polysaccharides within ECM act as a molecular glue, driving the bacterial cells to adhere to each other as well as surfaces.^31^ We thereby hypothesized that coralinones enhance the production of ECM and promote intercellular adhesion. To test this assumption, the microscopic changes in the surface properties of SDU70 cells were visualized by scanning electron microscopy (SEM). An abundance of smeared ECM tightly glued cells of SDU70-wt and SDU70-*corA*, which was not observed for nonpelleting strain SDU70-*ΔcorA*. Likewise, the surface of DK1622-wt and the mutant DK1622-vector appeared smooth, in contrast to DK1622-*corA* coated with a dot-like scabrous (Figure 6A). To gain a deeper insight into the composites of the secreted ECM, we measured EPS production of DK1622 using the trypan blue binding assay.^32^ As a consequence, EPS produced by DK1622-*corA* was around four times that by DK1622-wt or DK1622-vector. The cumulation of EPS was also observed for DK1622-wt supplemented with 5 mg/L **1** (Figure 6B). More intuitively, confocal laser scanning microscopy (CLSM) in combination with specific indicator dyes was employed to *in situ* visualize the distributions of cells and EPS in the biomass of DK1622 samples (DK1622-wt, DK1622-vector, DK1622-*corA*, and DK1622+**1**). Counterstaining with the mixture of dyes SYTO9, SYTOX, and wheat germ agglutinin (WGA) presented different fluorescences and thus allowed differentiation of EPS from the alive and debris cells. EPS given by DK1622-wt and DK1622-vector were obviously less than that of DK1622-*corA*, and DK1622+**1**, as represented by the evident blue signal in the latter two groups after overlaying the images derived from the three indicator dyes (Figure 6C). The experimental evidence from SEM, trypan blue binding assay, and CLSM, demonstrated that coralinone promoted EPS production, which would contribute to the agglutination of *C. exiguus* SDU70 and *M. xanthus* DK1622 in liquid cultures.

### Phylogenetic Contextualization of *cor*-Like BGCs in Myxobacteria

**Figure 7:**
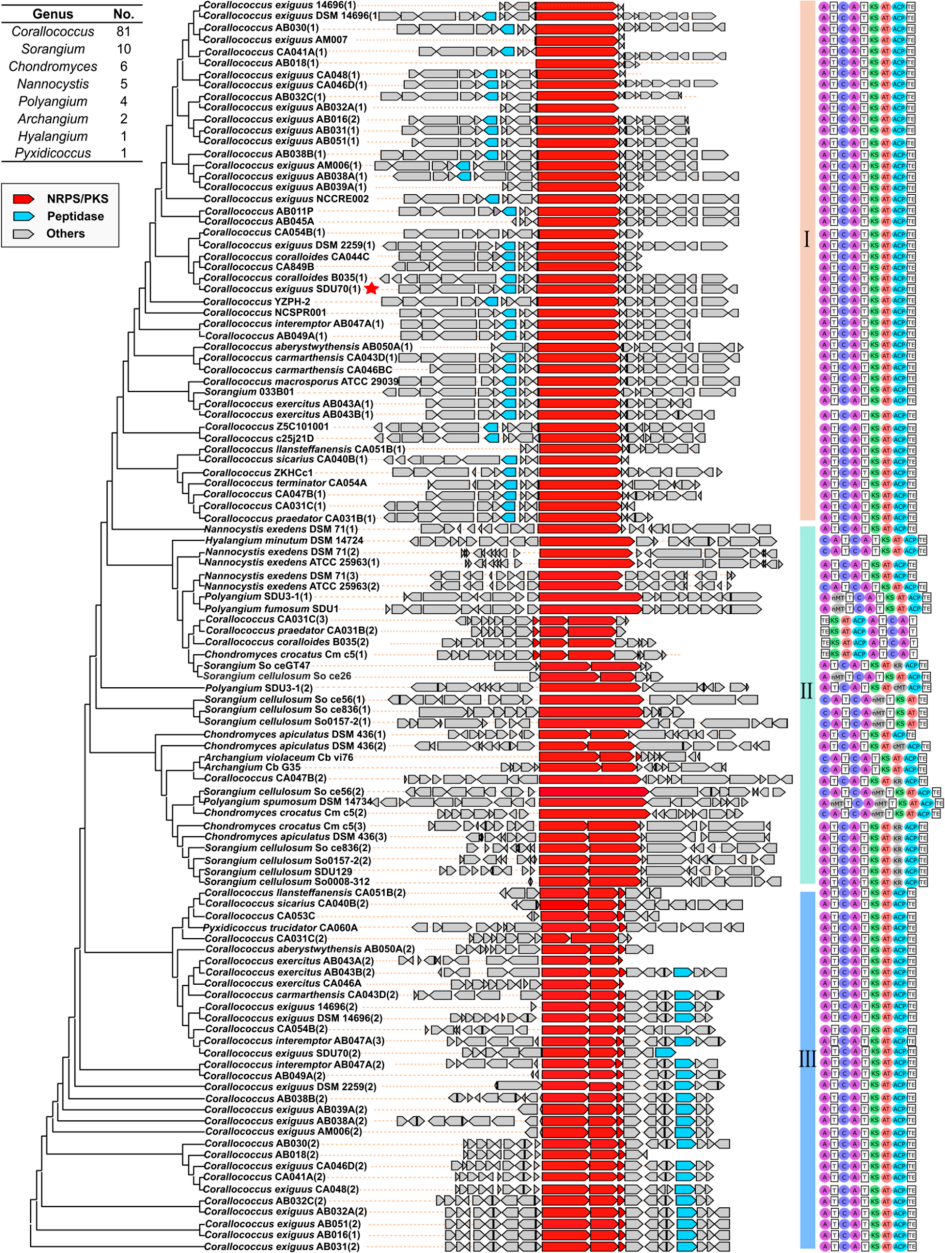
Phylogenetic analysis of *cor*-like BGCs in relation to 5-alkylated pyrazinones. The number of BGCs originating from each genus are summarized on the top left. The central NRPS/PKS genes are depicted in red, and the self-resistance genes homologous to *corB* encoding a putative peptidase are in blue. The *cor* BGC from SDU70 specifying coralinones is labeled with a red star. The domain organization of NRPS and PKS is appended at the right of each cluster. The phylogenetic tree is divided into three clades according to the domain organization of the NRPS/PKS megasynthetases. Abbreviations: A, adenylation domain; C, condensation domain: T, thiolation domain; KS, ketosynthase; AT, acyltransferase; KR, ketoreductase; ACP, acyl carrier protein; nMT, *N*-methyltransferase; cMT, *C*-methyltransferase; TE, thioesterase.

The classification of the enzymology governing the biosynthesis of coralinones prompted us to systematically survey the distribution of BGCs specifying 5-alkylated pyrazinones in myxobacteria. We analyzed all the myxobacterial genomes available to us by antiSMASH,^33^ and identified 110 putative BGCs that are closely associated with the biosynthesis of 5-methylated pyrazinones. Afterward, a multilocus phylogeny of the 110 BGCs was constructed by using CORASON tool^34^ to render the evolutionary relationships, wherein the source organisms and domain organization of central NRPS/PKS megasynthetases were also indicated (Figure 7). Interestingly, the alleged hybrid NRPS/PKS BGCs specifying 5-methylated pyrazinones are dominantly distributed in *Corallococcus*, followed by *Sorangium*, *Chondromyces*, *Nannocystis*, *Polyangium*, while none of them is derived from the genus *Myxococcus* that is so far the best cultivated and most genome-sequenced in the phylum of myxobacteria. The grouping of *cor*-like BGCs in the phylogenetic tree is generally genus-dependent, whereas the architectural arrangements are variable. Although the central NRPS/PKS genes show significant variance in size and arrangement, their total domain elements are invariably amenable to assemble two amino acids (prerequisite for pyrazinone backbone formation) and execute one round of PKS elongation (crucial for methylation/alkylation at C-5 of pyrazinone backbone). The phylogenetic tree could be classified into three clades according to the domain organization of the central NRPS/PKS. Clades I and III are predominantly derived from the genus *Corallococcus*, which contain a minimal set of domains indispensable for the biosynthesis of 5-methylated pyrazinones, via A-T-C-A-T-KS-AT-ACP-TE as exemplified in *corA*. These two clades differ in the splitting pattern and arrangement of NRPS/PKS genes as well as the standalone TE gene. By contrast, clade II shows much higher variation in the domain compositions, and most members are originated from other genera rather than *Corallococcus*. Other catalytic domains (KR, nMT, and cMT) are embedded into the minimal set of NRPS-PKS modules, which are expected to tune the canonical assembly line of 5-methylated pyrazinones by introducing modifications of reduction of β-ketone, *N*-methylation, and *C*-methylation, respectively. From the perspective of evolution, such divergent biosynthesis of 5-alkyated pyrazinones might confer advantages to their respective hosts. The unraveled flexibility in the domain arrangements of *cor*-like BGCs would not only underpin the discovery of many more natural 5-alkyated pyrazinones from our ever-growing myxobacterial collections but also provide clues for the bioinformatics-directed biomimetic total synthesis of unnatural 5-alkyated pyrazinones. All in all, the blueprint of the evolutionary scenario of the *cor*-like BGCs adds a new dimension to illustrate the correspondence between genetic and molecular variations of 5-alkyated pyrazinones.

### The Gene *corB* Is a Self-resistance Gene against Coralinone

**Figure 8:**
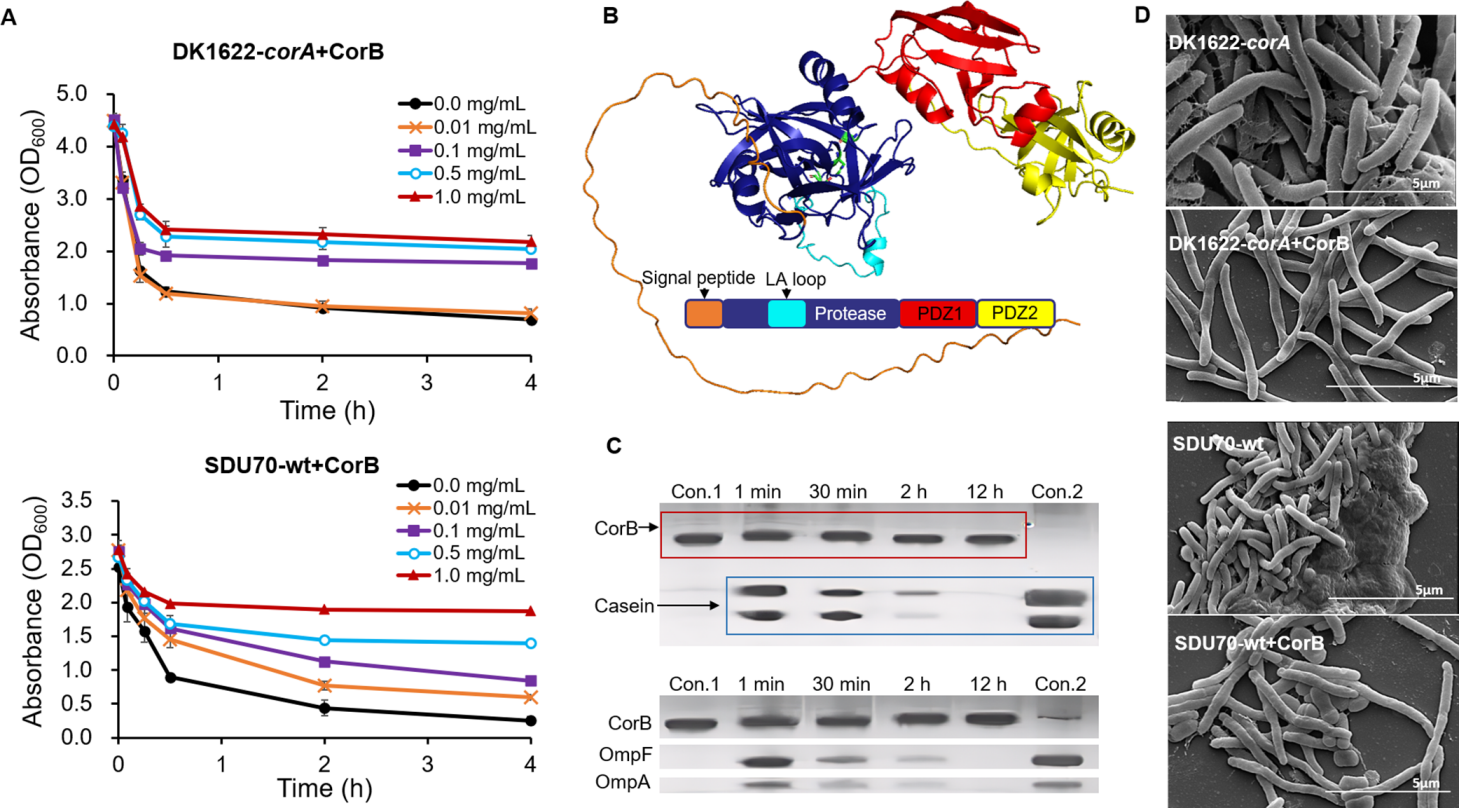
The gene *corB* encodes a protease to antagonize the agglutination-inducing effect of the cognate products encoded by *corA*. (A) Agglutination curve of SDU70-wt and/or DK1622-*corA* grown in the presence of 0.01–1 mg/L of CorB. The concentration of CorB was in a reverse relationship with the agglutination. Each point in the agglutination assay curve is the average of data calculated from three experiments; (B) AlphaFold2 prediction of 3D structure of CorB and schematic diagram functional domains typical of endopeptidase; (C) SDS–PAGE of the cleavage of different substrates by CorB (40 μM). The cleavage reactions with commercial casein and outer-membrane proteins (OmpF and OmpA) isolated from *E. coli* were performed at 37 °C and stopped at various time points. Each enzymatic degradation experiment was repeated three times. (D) SEM imaging of the biomass of SDU70-wt and/or DK1622-*corA* treated with or without 1 mg/L CorB. The ECM could be efficiently degraded by CorB.

We noticed that a peptidase gene (*corB*) is invariably coclustered with *corA*, especially for the *cor*-like BGCs from *Corallococcus* (Figure 7). To substantiate its function(s), *corB* was overexpressed in SDU70 using the strong constitutive promoter J23104.^35^ As a consequence, the clumping phenomenon was attenuated in the resultant overexpression strain SDU70-*corB* in comparison with SDU70-wt. We also constructed a strain SDU70-*ΔcorA-corB*, wherein *corB* was constitutively overexpressed and *corA* was concurrently inactivated. This double mutation maintained the dispersed morphology in spite of induction with 5 mg/L of **1** or **1b′**. SDU70-*corB* and SDU70-*ΔcorA-corB* did not clump at the presence of as high as 50 mg/L of **1** or **1b′**. The function of *corB* was further consolidated by the heterologous expression in DK1622. Overexpression of *corB* (under the control of promoter Tn5^35^) in DK1622 substantially abated agglutination induced by the endogenous expression of *corA* and/or exogenous addition of 5 mg/L of **1** or **1b′**. The threshold concentration that caused visible flocculation of DK1622-*corB* was 3–4 times higher than that for DK1622-wt (Figures S12–S14). In addition, *corB* was expressed in *E. coli* BL21(DE3), and the purified protein CorB was added to SDU70-wt and DK1622-*corA* at a gradient of concentration (0.01–1 mg/L). The flocculation of these two strains was indeed suppressed by exogenously supplemented CorB in a dose-dependent manner (Figure 8A). Therefore, both the in *vivo* and *in vitro* results unambiguously demonstrated that *corB* is a self-resistance gene^36^ that antagonizes the agglutination-inducing effect of cognate products encoded by *corA*. Someone might question why SDU70-wt severely clumped since it natively contains a copy of gene *corB*. We inferred that this discrepancy is due to the insufficient strength of innate promoter of *corB* in SDU70-wt, which leads to a lower expression level compared with that of strains DK1622-*corB* and SDU70-*corB* equipped with a much stronger promoter. Admittedly, RT-qPCR experiments confirmed that the transcription quantity of *corA* was around six times that of *corB* in SDU70-wt (Figure S15).

We were curious as to how *corB* bestows resistance to coralinone. The *in silico* analysis of the protein product of *corB* using BLASTp and HHpred indicated CorB belongs to the “DegP/Q family serine endoprotease”. On account of the peptidic origin of **1** and **1b′**, we initially speculated that enzymatic hydrolysis is the underlying mechanism. CorB was assessed its ability to hydrolyze **1** and/or **1b′***in vitro*. However, HPLC-DAD analysis indicated that both substrates remained unchanged in the reaction system (Figure S16). Ligand binding is not the mode of action, as judged by the microscale thermophoresis (MST) assay (Figure S17). These experiments compelled us to bioinformatically scrutinize its characteristics. Sequence alignment of CorB with the peptidases DegP and DegQ confirmed the conservation of key amino acid residues (His147, Asp177, and Ser252) requisite for the proteolysis activity^37^ (Figure S18). AlphaFold2^38^ prediction demonstrated that CorB shares a similar 3D structure with DegP and DegQ (Figure S19), containing one protease domain and two PDZ domains (Figure 8B).^37,39,40^ The existence of the signal peptide sequence implicates CorB is secreted as an extracellular enzyme. As the proteases DegP and PegQ are normally involved in degrading abnormal periplasmic and/or misfolded proteins in *E. coli*,^37,39,40^ we thus conceived that CorB hydrolyzes the proteins in ECM to degrade the framework of this so-called molecular glue, and accordingly antagonizes the agglutination-inducing effect of the cognate products encoded by *corA*. To test this supposition, CorB was first experimentally assayed its proteolytic activity toward the commercial casein. Next, CorB was found to be capable of degrading the outer-membrane proteins (OmpA and OmpF) isolated from *E. coli* cells in a time-dependent manner (Figure 8C). Finally, the biomass of SDU70-wt and DK1622-*corA* were treated with 1 mg/mL of CorB, subsequent observation using SEM confirmed that ECM of these two strains were substantially eliminated (Figure 8D). Taken together, these data strongly suggested that proteolysis is the underlying self-resistance mechanism imparted by CorB.

## Discussion

Myxobacteria are one of the gifted producers of a large array of secondary metabolites and potentially industrial enzymes.^2^ Although many myxobacterial NPs have been described for potential therapeutic applications, such as “cytotoxic”, “antibacterial”, “antifungal”, and “anti-inflammatory”, the bioactivities of a considerable number of sophisticated chemical scaffolds still remain ambiguous.^3^ In fact, microorganisms produce NPs for their own benefit, and the encrypted secondary metabolites useless in therapeutic applications might play an important role in coordinating the physiological development *in situ*. Of note, myxobacteria are a fertile ground for such chemical ecology scenarios. For instance, the model *Myxococcus xanthus* produces a small-molecule pigment DKxanthene for modulating sporulation process.^41^ Ambruticin from *Sorangium cellulosum* affects fruiting body formation of *M. xanthus* under starvation.^42^ Antibiotic TA (myxovirescin) plays an important role in the predatory behavior of *M. xanthus*.^43^ The lipid stigmalone produced by *Stigmatella aurantiaca* functions as a pheromone that induces cellular aggregation and enhances fruiting body formation.^44^ Coralinone represents the first experimentally validated case for the small-molecule-regulated agglutination of myxobacteria in liquid cultures. Romanowski and co-workers recently identified the lipodepsipeptide selethramide that promotes motility of its native producer belonging to Gram-negative *Burkholderia*, a bacterial family sharing considerable physiological similarities to myxobacteria.^45^ These examples strongly support a close connection between developmental growth and sophisticated small molecules deliberately encoded by myxobacteria. It is conceivable that many more alike cases remain to be unveiled, which will provide a new avenue to investigate the sociobiology of myxobacteria. In turn, it might be particularly fruitful to exploit the ecology-based strategies to unlock the silent BGCs^46^ for continued natural products discovery from myxobacteria, given their distinct life patterns (social behavior, predation, swarming, etc).

The aggregation phenotypes actually occur in most familiar NPs producers, such as actinomycetes and myxobacteria. From the perspective of ecology, flocculation (and biofilm formation alike) is a protective social response that shields cells from stressful environments.^47^ As diffusion in flocs is severely impaired, the outer cells protect the inner cells from harmful challenges that cannot be achieved by single cells alone. On the other hand, the pellet formation restricts the efficient transfer of nutrients and gases to the center, which is adverse to growth and culture heterogeneity and thus lowers the maximal obtainable product yield.^32,48^ For myxobacteria, cellular aggregation has been previously ascribed to peptidic substances like A-factors^49,50^ and/or C-factor.^51^ The involvement of small-molecule morphogens in coordinating this physiological process is surprising to us. It is yet unclear if mechanistic connections exist for the signaling pathways of coralinone and A-factors or C-factor. In addition, the scenario that *corA* autonomously encodes the small molecules regulating the morphogenesis of myxobacterial cells is reminiscent of the quorum-sensing (QS) circuitry ubiquitous in microbes. QS systems function to control cell density-dependent processes, such as LuxI/LuxR in *Vibrio fischeri*,^52^ EsaI/EsaR in *Pantoea stewartii*,^53^ and the γ-butyrolactones (GBLs) signaling systems in streptomycetes.^54^ Typically, each QS system is composed of a core synthase (*e.g.* LuxI) governing the biosynthesis of specific small-molecule signals (*e.g*. acyl-homoserine lactone), and a cognate signal-responsive transcription regulator (*e.g*. LuxR). Therefore, unraveling the molecular underpinnings and/or signaling circuits of coralinone will lay foundations for developing the *corA*-based biosensor for perturbing and rewiring the underlying regulatory gene networks to achieve a trade-off between essential pathways and product synthesis. Although it is currently arduous to identify the receptor(s) of coralinone, we speculate that it interacts with an anonymous global regulator and thus impinge on multiple chemosensory signal transduction pathways to display multifaceted regulatory effects.

Strictly speaking, self-resistance means that antibiotic-producing bacteria develop necessary survival adaptation to prevent self-toxicity caused by the produced antibiotics.^55^ In general, the self-resistance strategies include export and reduced influx, target modification, sequestration, and enzymatic inactivation of antibiotics.^56^ Although the metabolites encoded by *corA* are not chemical weapons, the antagonism between the products encoded by *corA* and *corB* conforms to the paradigm of self-resistance. We summarized the relationship between *corA* and *corB*: *corA* autonomously specifies coralinone following the textbook NRPS/PKS collinearity assembly line. The PKS moiety is responsible for the installation of 5-methyl on the pyrazinone backbone to exert the agglutination-inducing activity through enhancing the secretion of extracellular substances. The gene *corB* encodes a protease that is exported outside the cell and hydrolyzes extracellular proteins to abate aggregation. It was the first report that bacteria devise a unique self-resistance machinery for the purpose of regulating growth development instead of survival competition. There is an enticing opportunity to understand mechanistically how SDU70 regulates the transcription level of *corA* and *corB* to fine-tune the status of aggregation, which would open a new avenue to study social interactions of myxobacteria, such as biofilm formation, swarming, fruiting body, and predation, among others. Moreover, the concerted evidence for functional relationship between *corA* and *corB* in two distinct genera *Myxococcus* and *Corallococcus*, alongside the phylogenetic contextualization of *cor*-like BGCs in myxobacterial realm, imply the generality of our findings.

With respect to the biotechnological applications, the physiological relevance of the *cor*-like pathway and especially the self-resistance mechanism of coralinones apparently provide the impetus to develop foundamental counter-measures to circumvent the tricky fermentative flocculation of myxobacteria in industry.^28^ As well, most nonmodel myxobacterial species are barely tractable to *in situ* genetic manipulation.^57^ The cellular aggregation intercepts the entry of exogenous DNA into the cells and thus hinders the genetic manipulation. The elucidation of the self-resistance mechanism of coralinone would direct our efforts to tackle the genetic recalcitrance of myxobacteria, which is critical for the development of an efficient chassis for the heterologous expression of myxobacterial BGCs.

All in all, the biosynthetic elaboration of coralinone furthers our appreciation of hybrid NRPS-PKS assembly line chemistry and would be undoubtedly a boon for the precise control of production and genetic reprogramming of NPs structurally related to 5-alkylated pyrazinones. The acquired knowledge that coralinone serves as a signaling molecule that regulates the aggregation process of myxobacteria merits to be further explored in the future for the maximal excavation of these largely underexplored resources.

## Methods

### General Experimental Procedures

Medium pressure liquid chromatography (MPLC) was performed using a Buchi Pure C-810 Flash apparatus equipped with FlashPure EcoFlex C_18_ columns. Gel chromatography was packed by Sephadex LH-20 gel (GE). HPLC analysis was performed with an Agilent 1260 series HPLC apparatus (Agilent technologies Inc., Santa Clara, CA, U.S.A.), using a 250 × 4.6 mm Luna 5 μm C_18_ (2) 100 Å column equipped with a guard column containing C_18_ 4 × 3 mm cartridges (Phenomenex Inc., Torrance, CA, U.S.A.). NMR spectra were acquired on a Bruker Avance III 600 spectrometer with TMS as an internal reference. The HR-Q-TOF ESI-MS analyses were performed on a rapid separation liquid chromatography system (Dionex, UltiMate3000, UHPLC) coupled to an ESI-Q-TOF mass spectrometer (Bruker Daltonics, Impact HD). TLC analysis was developed by precoated silica gel GF_254_ (Qingdao Haiyang Chemical Co., Ltd., Qingdao, China). According to specific experiments, all organic solvents and chemicals were analytical or HPLC grade. The ClonExpress II One Step Cloning Kit was used for Gibson cloning (Vazyme). PCR products were purified from agarose gels by using the Cycle Pure Kit (Omega). Plasmids were prepared by using the QIAprep spin miniprep kit (QIAGEN). Phanta Super-Fidelity DNA Polymerase was used in all PCRs according to the supplier’s instructions (Vazyme). *E. coli* cells were disrupted using a NanoGenizer (Genizer LLC) high-pressure homogenizer for proteins purification.

### Bacterial Strains, Plasmids, and Growth Conditions

The details of the plasmids and strains utilized in this study were provided in Table S3 and S4. *E. coli* strains were cultivated in Luria–Bertani (LB) medium (10 g/L tryptone, 5 g/L yeast extract, 5 g/L NaCl, pH 7.0). For the growth of myxobacteria, CTT (10 g/L Casein peptone, 1.97 g/L MgSO_4_·7H_2_O, 10 mL of 1 M Tris-HCl, pH 7.6), 10 mL of 0.1 M PBS), and VY/2 (5 g/L Baker yeast, 1 g/L CaCl_2_, 0.5 g/L MgSO_4_·7H_2_O) media were employed. Where needed, the medium was supplemented with appropriate antibiotics (40 μg/mL kanamycin, 100 μg/mL ampicillin, 25 μg/mL chloramphenicol, or 50 μg/mL apramycin) to achieve selective pressure. All the myxobacterial strains were shaken at 30 °C, 200 rpm for growth.

### Isolation and Identification of *C. exiguus* SDU70

The myxobacterium SDU70 was isolated from a soil sample collected from the campus of Shandong University (Qingdao, China), according to the methodology described before.^58^ The 16S rRNA gene of the strain was amplified with the universal primers 27F (5′-AGTTTGATCCTGGCTCAG-3′) and 1492R (5′-TACCTTGTTACGACTT-3′). The PCR product was sent for Illumina barcode sequencing (Beijing Qingke Biotechnology Co., LTD). The resultant 16S rRNA gene sequence was subjected to BLASTN analysis in the NCBI database (https://www.ncbi.nlm.nih.gov/) and/or EzBioCloud (http://www.ezbiocloud.net/), which returned the highest similarity (99.93%) to the strain *Corallococcus exiguus* DSM 14696 (Figure S20). The 16S rRNA sequence of SDU70 was deposited at the National Center for Biotechnology Information (NCBI) under access number SAMN27682953.

### Isotope Labeling

SDU70 was transferred to 100 mL liquid medium and grown for 36 h, and then fed with l-leucine *d*_3_, l-isoleucine *d*_10,_^13^C-formic acid, ^13^C-formaldehyde or *S*-(5′-adenosyl)- l-methionine-*d*_3_ at a final concentration of 1 mM, respectively. After an additional 5.5 days of cultivation, the supernatant was harvested by centrifugation and extracted by 0.2 g of HP-20 resin. Then, the adsorbed compounds were eluted with 5 mL of methanol. The organic layer was concentrated by reduced pressure at 40 °C, and the crude extract was redissolved in 0.5 mL of methanol for LC-MS analysis.

### Isolation of Coralinones from *C. exiguus* SDU70

The strain was grown in VY/2 medium at 30 °C and 200 rpm for 7 days. All the cultures (34 L) were combined and the cell mass were removed by filtration. Around 0.68 kg of Diaion HP-20 resin were added to the supernatant and shaken for 12 h. The resin was washed with H_2_O containing 1% MeOH and then eluted by MeOH. After the organic solvent was removed under reduced pressure at 30 °C, the crude extracted was fractionated by reverse-phase preparative medium-pressure liquid chromatography (Buchi, Flawil, Japan) eluting with MeOH-H_2_O solvent system, to give 10 fractions (Fr1–Fr10). Fr6 that contained the compounds featuring with characteristic UV spectrum for pyrazinone backbone was further separated by semipreparative HPLC eluting with gradient eluent of acetonitrile in water from 30% to 50% containing 1‰ TFA in 30 min at a flow rate of 1.8 mL/min, to afford compound **1** (3.8 mg) and **2** (7.2 mg).

### Genome Sequencing, Assembly, and Annotation

Genome sequencing, assembly, and annotation were performed according to our previously published reports.^59,60^ Briefly, genomic DNA of SDU70 was extracted by a whole genome DNA sequencing kit (Oxford Nanopore Technologies Inc., Oxford, U.K.). Genome sequencing was conducted by the joint use of Illumina and Nanopore technologies (Wuhan Benagen Technology Co., Ltd.). The processed reads were assembled by Unicycler (0.4.8) software (https://github.com/rrwick/Unicycler). The annotations of genes were done based on the BLAST against databases COG (https://www.ncbi.nlm.nih.gov/COG/), KEGG (https://www.kegg.jp/kegg/), Refseq (https://www.ncbi.nlm.nih.gov/refseq/), Uniprot (https://www.uniprot.org/).

### Heterologous Expression of *cor* Gene Cluster in *E. coli*

The full-length of *corA* (8.6 kb) was PCR amplified from SDU70 genomic DNA, and cloned into the PCR-linearized pET28a vector through Gibson cloning. The resultant recombinant plasmid *corA-*pET28a and empty vector pET28a were electroplorated into *E. coli* BAP1.^25^ Single colony was inoculated in 5 mL of liquid LB medium containing kanamycin and shaken at 220 rpm and 37 °C. One mL of overnight culture was diluted 100 times with LB medium and grown until OD_600_ ∼ 0.6. Then, 0.2 mM isopropyl β-D-thiogalactopyranoside (IPTG) was added to induce protein expression at 180 rpm and 16 °C, and the culture was incubated for another 24 h. The cells were harvested by centrifugation at 6,000*g* for protein purification (see below). The supernatant was extracted with 100 mL of ethyl acetate. The organic layer was evaporated under a vacuum at 40 °C, and the residue was dissolved in 0.5 mL of methanol for HPLC-DAD analysis. HPLC program was as follows: 0–30 min, 5–100% ACN in H_2_O; 30–40 min, 100% ACN in H_2_O; 40–41 min 100% ACN in H_2_O; 41–46 min 5% ACN in H_2_O. Each experiment was done in triplicate.

### Enzymatic Reactions Catalyzed by CorA and CorA-PKS

*E. coli* BAP1 cells expressing proteins CorA or CorA-PKS were suspended in 50 mL of lysis buffer (20 mM Tris, 500 mM NaCl, 10 mM imidazole, 10% glycerol, pH 8.0). Cell suspensions were sonicated on ice, and the resultant debris were removed by centrifugation at 10,000*g* for 60 min at 4 °C. The His_6_-tagged proteins in the supernatant were adsorbed by Ni-NTA column (GE Healthcare, USA) and then recovered with imidazole in Tris-HCl buffer (pH 8.0) according to the manufacturer’s protocol. The semipurified proteins were further purified by Superdex 200 pg gel-filtration column (GE Healthcare), eluting with a buffer of 20 mM Tris-HCl (pH 8.0) and 150 mM NaCl. The obtained *holo*-form of proteins were used for enzymatic reactions. The reaction system for CorA was as follows: enzyme CorA (10 μM), CoA (2 mM), ATP (3 mM), NADPH (10 mM), Mal-CoA (2 mM), l-amino acids (2 mM), MgCl_2_ (10 mM), NaCl (50 mM), and TCEP (0.5 mM) in a 100 μL of Tris-HCl (100 mM, pH 8.0) buffer. The reaction system for CorA-PKS was basically same with CorA, except the l-amino acids was substituted by chemically synthesized Leu-Val-SNAC. After incubation at 37 °C for 4 h, enzymatic reactions were quenched with 200 μL of ice-cold methanol. The metamorphic proteins were removed by high-speed centrifugation at 12,000*g* for 10 min, and the supernatant was analyzed by HPLC-DAD.

### ATP–PPi Exchange Assay

This experiment basically followed the method reported by Wilson and Aldrich.^26^ Specifically, the reaction system contains 10 mM amino acid substrates, 2 mM ATP (Solarbio), 0.2 mM MESG (Medchemexpress), 1 U/mL purine nucleoside phosphorylase (Shanghai yuanye Bio-Technology Co., Ltd.), 0.4 U/mL inorganic pyrophosphatase (Sigma), 150 mM hydroxylamine (Sigma) and 2 μM proteins in 100 μL buffer (50 mM Tris, 200 mM NaCl and 5 mM MgCl_2_, pH 8.0). The value was read at A360 in a SpectraMax M5 plate reader (Molecular Devices, Sunnyvale, California) in 96 well, clear-bottomed plates (Corning). A360 values were converted to pyrophosphate release, by comparing with a standard curve (KH_2_PO_4_ from 2 μM to 128 μM) for known quantities of pyrophosphate.

### Organic Synthesis

The organic synthesis and spectral data assignment of Leu-Val-SNAC, **1**, **1a′′**, **1b′**, **1e**, and **8** were detailed in Supporting Information.

### Promoter Exchange of *corA* and *corB* in *C. exiguus* SDU70

The first ∼1 kb of *corA* and *corB* (counting from the start codon ATG) was PCR amplified from the SDU70 genome, whereby the promoter J23104 or Tn5 was added into the forward primers, respectively (Table S5). The resulting fragments Tn5-*corA* and J23104-*corB* were ligated with the linearized plasmid pBJ113 by Gibson assembly to afford the overexpression plasmid pBJ113-*corA* and pBJ113-*corB*. The resultant constructs were electroporated into SDU70 to instigate single-crossover recombination (Figure S21). The correct mutants SDU70-*corA* and SDU70-*corB* were selected by 40 μg/mL kanamycin and further checked by tandem colony PCR with Sanger sequencing.

### Insertional Disruption of *corA* in *C. exiguus* SDU70

A ∼1 kb homologous arm was PCR amplified from the middle region of *corA*. The resultant product was ligated with the linearized plasmid pBJ113 by Gibson assembly. The knockout construct pBJ113-*ΔcorA* obtained was then transferred via electroporation into SDU70 for single-crossover recombination (Figure S21). Inactivation mutants were identified by kanamycin resistance, PCR verification, and Sanger sequencing.

### Concurrent Disruption of *corA* and Overexpression of *corB* in *C. exiguus* SDU70

The aforementioned DNA fragment J23104-*corB* was cloned into plasmid pBJ113-*ΔcorA* to construct the double mutation construct pBJ113-*ΔcorA*-J23104-*corB*. The obtained construct was electroporated into SDU70 followed by kanamycin selection for double mutant SDU70-*ΔcorA*-*corB*. The primer pair *ΔcorA*-*corB*-check F/R (Table S5) distinguished the desired single-crossover recombination at *corA* from the undesired single-crossover recombination at *corB* (Figure S21).

### Heterologous Expression of *corA* and/or *corB* in *M. xanthus* DK1622

The full length of *corA* and/or *corB* were cloned into the integrative plasmid pSWU19^61^ under the control of the promoter Tn5 (Figure S22).^35^ The recombinant plasmids were then transferred via electroporation to DK1622 cells. The correct mutants were selected by the kanamycin resistance, followed by PCR validation (Table S5).

### Agglutination Assay

The agglutination assay basically followed the method described by Velicer.^62^ Briefly, DK1622 strains were cultured in CTT medium, whereas SDU70 strains were cultured in VY/2 medium. The aggregation-inducing compounds were added together with the initial inocula. After 3 days of growth, ∼2 mL of liquid cultures (in logarithmic growth stage) were transferred into cuvettes. Samples were left static for 15 min to visualize the clumping phenomenon. To plot the agglutination curves, the absorbance readings (600 nm) were measured on a UV-8000 spectrophotometer at differential intervals within 4 h. Decreasing the absorbance represents the settling of agglutinated cell clumps. Data points are the means of three replicates per strain.

### Microscopy

The microscopic observation of SDU70 and DK1622 basically followed our recently published methods.^63^*Scanning electron microscopy (SEM):* DK1622 and SDU70 strains (wild type and mutants) were grown to logarithmic stage. The cells were washed three times with 1× PBS buffer and then fixed overnight at 4 °C in 2.5% glutaraldehyde solution. Samples were prepared using the standard ethanol dehydration method,^62^ and observed by QUANTA FEG250 scanning electron microscope (Thermo Fisher Scientific, USA). *Confocal laser scanning microscopy (CLSM)*: DK1622 strains were grown in CTT liquid medium for 24 h, while SDU70 strains were grown in VY/2 medium for 72 h. The presence of EPS, eDNA dead cells, and alive cells were labeled with Alexa 350-labeled wheat germ lectin (WGA), SYTOX Orange, and STYO 9, respectively. The images were taken on LSM 900 CLSM (Zeiss, Germany), using 63× oil immersed objective lens. The CLSM images were captured by ZEN software (Zeiss, Germany) and exported by using ImageJ software.

### Trypan Blue Binding Assay

Exopolysaccharide (EPS) production was measured according to the method published by Black et al.^64^ Briefly, wild type and mutant strains of DK1622 were inoculated in liquid CTT medium and incubated for 24 h at 30 °C and 200 rpm. The cells grown to the logarithmic phase were collected by centrifugation, and the supernatants were discarded. Cells were resuspended and adjusted to a density of 5 × 10^8^ CFU/mL with TPM buffer (1.97 g/L MgSO_4_·7H_2_O, 1 M Tris-HCl, 10 mL 0.1 M PBS, pH 7.6). Next, the cell suspension was evenly mixed with an equal volume of Trypan Blue dye solution (150 μg/mL), and TPM buffer was used as a blank control. The supernatant was centrifuged at 12000 rpm for 5 min, and 200 μL was used for the absorbance measurement at 585 nm. Each experiment was performed in triplicate.

### Extraction of Bacterial Outer Membrane Proteins

The bacteria were cultured and harvested as described above. Cells were resuspended in precooled Tris-Mg^2+^ buffer (pH 7.3), and then sonicated on ice, followed by centrifugation at 3,000*g*, 4 °C for 10 min to remove the unbroken bacteria. The resultant supernatant was further centrifuged at 10,000*g*, 4 °C for 60 min to remove the soluble proteins.

### Proteolytic Assay of CorB

In a 50 μL proteolysis reaction system, 40 μM CorB and 130 μM substrate proteins were incubated in a buffer solution (25 mM Hepes–NaOH pH 7.5, 150 mM NaCl and 5 mM MgCl_2_) at 37 °C. At differential time points (1 min; 30 min; 1, 2, and 12 h), reactions were stopped by SDS loading buffer supplemented with 8 M urea. Subsequently, the reaction solution was incubated at 95 °C for 15 min, and the degradation of substrate proteins was detected by SDS-PAGE.

### Construction of Phylogenetic Tree of *cor*-like BGCs

In total, all the 253 myxobacterial genomes deposited in RefSeq database (https://www.ncbi.nlm.nih.gov/refseq/) were downloaded as of mid-2022 and analyzed using antiSMASH 6.0^33^ to identify biosynthetic gene clusters (BGCs). Subsequently, we wrote a Python script to fetch the targeted BGCs from the obtained data set. The constraints were: 1) no more than three adjacent genes were NRPS and/or PKS genes; 2) NRPS/PKS contain two A domains, no more than one C domain, one KS domain, one AT domain, one TE domain; 3) other variable domains (*e.g*. KR or MT) could be flexibly incorporated. The returned 135 hits were manually checked and trimmed to filter out 110 putative BGCs that were closely associated with the biosynthesis of 5-methylated pyrazinones. Phylogenetic comparison of all the *cor*-like BGCs was performed by CORASON tool.^34^ The gene *corA* from *C. exiguus* SDU70 was used as the query gene. The e_value of “minimal for a gene to be considered a hit” was set to 3e^–174^, while all the other parameters of CORASON were set as default. The software MEGA 7.0 was used to the visualize the phylogenetic tree. The schematic diagram for module organization of the central NRPS and PKS was manually drawn according to the antiSMASH prediction.

## Supporting Information Available

Supplementary experimental methods; structure elucidation of coralinones; biomimetic total synthesis of coralinone A; annotation of the *cor* gene cluster; cytotoxic activities, NMR data assignments, and X-ray crystallography data of coralinones; details of the strains, plasmids, and primers used in this study; metabolic profiling of strains; agglutination assays of strains; biochemical investigation of the recombinant enzymes; bioinformatic analysis of protein CorB; diagrammatic sketch for the genetic manipulation including gene disruption and heterologous expression; spectra of compounds (PDF)
Transparent Peer Review report available (PDF)
