# Supplementary material for: Deciphering the Biosynthesis and Physiological Function of 5-Methylated Pyrazinones Produced by Myxobacteria
Source: ACS Cent Sci. 2024 Feb 7;10(3):555–68. doi: 10.1021/acscentsci.3c01363 (PMC10979478; doi:10.1021/acscentsci.3c01363)
Supplement: Supplementary file 1 — oc3c01363_si_001.pdf [file oc3c01363_si_001.pdf]

Supporting Information for the manuscript:

Deciphering The Biosynthesis and Physiological Function of 5-Methylated Pyrazinones Produced by Myxobacteria

Le-Le Zhu,[⊥] Qing-Yu Yang,[⊥] De-Gao Wang,[⊥] Luo Niu, Zhuo Pan, Shengying Li, Yue-Zhong Li,* Wei Zhang,* and Changsheng Wu*

State Key Laboratory of Microbial Technology, Institute of Microbial Technology, Shandong University, 266237 Qingdao, P.R. China

email: wuchangsheng@sdu.edu.cn; zhang_wei@sdu.edu.cn; lilab@sdu.edu.cn

* Corresponding authors emails: wuchangsheng@sdu.edu.cn (C.W.); zhang_wei@sdu.edu.cn (W.Z.); lilab@sdu.edu.cn (Y.Z.L.)

[⊥] These authors contributed equally

Table of contents

Supplementary experimental methods **4**

Structure elucidation of coralinones A and B. **5**

Biomimetic total synthesis of coralinone A (1) **6**

Supplementary tables **12**

Table S1. Annotations for *cor* gene cluster. 12

Table S2. Cytotoxic activity (IC_{50} in μM) for coralinones A (**1**) and B (**2**) on a panel of human cell lines.. 13

Table S3. Plasmids used in this study 14

Table S4. Strains used in this study. 15

Table S5. Primers used in this study. 16

Table S6. X-ray crystallographic data for coralinone B. 17

Table S7. ^1H and ^{13}C NMR data of coralinones A (**1**) and B (**2**) in methanol-d_4. 18

Supplementary graphs **19**

Figure S1. HPLC profiling of SDU70 crude extract. 19

Figure S2. ORTEP diagram of the crystal structure of coralinone B (**2**). 20

Figure S3. Isotope feeding experiment with L-leucine d_3 and L-isoleucine d_{10}. 21

Figure S4. SDS-PAGE analysis of recombinant proteins CorA, CorA-PKS, and CorB. 22

Figure S5. Combinational enzymatic synthesis of diversified coralinones by exploring the substrates promiscuity of CorA. 23

Figure S6. HR-ESI-MS data of the enzymatic products **1–7**. 24

Figure S7. The relative yield of compounds **1–6** produced by CorA during *in vitro* enzymatic assays. 25

Figure S8. Metabolic analysis verified the fidelity of genotypes after genetic manipulation of SDU70 and DK1622. 26

Figure S9. Compounds **1** and **1b'** induced the agglutination of strains SDU70-△corA and DK1622-wt in a dose-dependent manner. 27

Figure S10. Agglutination assay of the biosynthetically related compounds in relation to 5-unsubstituted and 5-methylated pyrazinones. 27

Figure S11. Biotransformation of 1b' by the strains SDU70-△corA and DK1622-wt. 28

Figure S12. corB is a self-resistance gene against the cognate products encoded by corA. 30

Figure S13. Agglutination assay of SDU70 and DK1622 mutants endowed with different genotypic combination of *corA* and *corB*. 31

Figure S14. Agglutination assay of *corB*-overexpression mutants treated with an increasing concentration of **1** or **1b'**. 32

Figure S15. Transcription of *corA* and *corB* in SDU70-wt. 33

Figure S16. Enzymatic hydrolysis of coralinones using CorB. 34

Figure S17. MST assay of CorB to coralinone A.. 35

Figure S18. Sequence alignment of CorB with the peptidases DegP and DegQ. 36

Figure S19. Comparison of 3D structures of CorB, DegP and DegQ. 37

Figure S20. Taxonomic identification of the myxobacterium SDU70. 38

Figure S21. Diagrammatic sketch for the disruption and/or overexpression of *corA* and *corB* in SDU70. 39

Figure S22. Schematic diagram for the plasmid construction for heterologous expression of *corA* and/or *corB* in DK1622. 40

All spectra employed in structure elucidation. **41**

S1. ^1H NMR (600 MHz, methanol-d_4) of coralinone A (**1**). 41

S2. ^{13}C NMR (150 MHz, methanol-d_4) spectrum of coralinone A (**1**). 41

S3. DEPTQ (150 MHz, methanol-d_4) spectrum of coralinone A (**1**). 42

S4. HSQC (600 MHz, methanol-d_4) spectrum of coralinone A (**1**). 42

S5. HMBC (600 MHz, methanol-d_4) spectrum of coralinone A (**1**). 43
S6. ^{1}H-^{1}H COSY (600 MHz, methanol-d_4) spectrum of coralinone A (**1**). 43
S7. ^{1}H NMR (600 MHz, methanol-d_4) of coralinone B (**2**). 44
S8.^{13}C NMR (150 MHz, methanol-d_4) spectrum of coralinone B (**2**). 44
S9. DEPTQ (150 MHz, methanol-d4) spectrum of coralinone B (**2**). 45
S10. HSQC (600 MHz, methanol-d_4) spectrum of coralinone B (**2**). 45
S11. HMBC (600 MHz, methanol-d_4) spectrum of coralinone B (**2**). 46
S12. ^{1}H-^{1}H COSY (600 MHz, methanol-d4) spectrum of coralinone B (**2**). 46
S13. HRESIMS spectrum of coralinone B (**2**). 47
S14. HRESIMS spectrum of coralinone A (**1**). 47
S15. ^{1}H NMR (600 MHz, methanol-d4) of Leu-Val-SNAC. 47
S16. DEPTQ (150 MHz, methanol-d_4) spectrum of Leu-Val-SNAC. 48
S17. ^{1}H NMR (600 MHz, $CDCl_3$) of 1**a''**. 48
S18. ^{13}C NMR (150 MHz, $CDCl_3$) of **1a''**. 49
S19. ^{1}H NMR (600 MHz, methanol-d_4) of cyclic-Leu-Val-ethyl formate. 49
S20.^{13}C NMR (150 MHz, methanol-d_4) spectrum of cyclic-Leu-Val-ethyl formate. 50
S21. ^{1}H NMR (600 MHz, $CDCl_3$) of **1b'**. 50
S22.^{13}C NMR (150 MHz, methanol-d_4) spectrum of **1b'**. 51
S23. ^{1}H NMR (600 MHz, methanol-d_4) of Boc-Leu-Val-hydroxy-methylhexanoic acid. 51
S24.^{13}C NMR (150 MHz, methanol-d_4) spectrum of Boc-Leu-Val-hydroxy-methylhexanoic acid. 52
S25. ^{1}H NMR (600 MHz, methanol-d_4) of pre-**8**. 52
S26.^{13}C NMR (150 MHz, methanol-d_4) spectrum of pre-**8**. 53
S27. ^{1}H NMR (600 MHz, $CDCl_3$) of **10b'**. 53
S28. ^{1}H NMR (600 MHz, methanol-d_4) of **10**. 54
S29. HRESIMS spectrum of synthesis compounds. 54
References. 57

Supplementary experimental methods

X-ray crystal structure determination of coralinone B (2). The white needle crystals of **2** were obtained from MeOH solvent by slowly volatilizing in a glass vial under ambient condition. The X-ray diffraction measurement of crystals basically followed our previously published method.[1] The crystallographic data for **2** was summarized in Table S6, and deposited in CCDC database under the access number 2278050.

Antibacterial Assay. The antibacterial assay was performed by agar diffusion method.[2] Single colony of Gram-positive bacteria *Staphylococcus aureus*, Gram-negative bacterium *Acinetobacter baumannii*, and fungus *Candida albicans* were picked and inoculated into 8 mL of LB liquid medium for overnight growth at 37 °C, respectively. Next day, the cultures were 100-fold diluted to $1x10^7$ CFU/mL with fresh LB liquid medium, to grow at 37 °C until OD_{600} 0.4~0.6. Compounds **1** and **2** were dissolved in methanol (2 mg/mL), and 20 μL of the solution was applied on a paper disk. The disks were then placed onto an agar plate containing a soft agar overlay with the indicator microorganisms. Kanamycin (for *S. aureus*) and apramycin (for *C. albicans* and *E. coli*) at a concentration of 1 mg/mL were used as positive controls, and the solvent methanol as the negative control. After incubation at 37 °C for 18 h, compounds **1** and **2** did not show any obvious growth inhibition zones against the tested strains.

Protease inhibition assay. In principle, the activity of cathepsin L (0.02 μg/mL) in MES buffer (50 mM MES, 5 mM DTT, pH 5.5) was detected by proteolysis of fluorescent substrate z-FR-AMC. Enzyme was pre-incubated with a gradient concentration (0~500 μM) of compounds for 60 min at 37 °C in a black 96-well plate. Then, 20 μM of z-FR-AMC was added to each well to make up 200 μL reaction system, and the reaction continued for another 30 min. The absorbance was measured by microplate reader (Spectra max GEMINI XPS, excitation wavelength 380 nm, emission wavelength 460 nm). The protease inhibitor E-64 was used as positive control and DMSO was the negative control. All the experiments were completed in triplicate.

Enzymatic cleavage of 1 and 1b' using CorB. The reaction was conducted in a 50 μL system comprising of 20 mM Tris-HCl buffer (pH 7.5), 150 mM NaCl, 0.22 mM CorB, and 0.05 mg **1**, or **1b'** (dissolved in DMSO) respectively. Tris-HCl buffer was used as a negative control, and all experiments were repeated in triplicates. The reactions were incubated at 37 °C for 12 hours and then terminated by adding 50 μL of MeOH. The mixture was centrifuged at 12000 rpm for 20 minutes, and the supernatant was subsequently analyzed HPLC-DAD.

MST assay. The purified CorB was mixed with an equal volume of RED-tris-NTA dye solution and incubated in the dark for 30 min, centrifuged at 12000 rpm, 4 °C for 10 min. Then, 10 μL of the CorB-dye complex was mixed with an equal volume of test compound solution ranging from 15.2 μM to 0.5 mM. The resulting samples were collected by capillary tubes and analyzed by Microscale Thermophoresis (Monolith NT.115).

Structure elucidation of coralinones A and B.

Coralinone A (**1**) was obtained as a white needle crystal with the formula $C_{12}H_{20}N_2O$, as established from the HRESIMS $[M+H]^+$ peak at *m/z* 209.1653 (calculated 209.1648 for $C_{12}H_{21}N_2O$), indicating four degrees of unsaturation. The ^1H NMR date (Table S7) combined with ^1H-^1H COSY spectrum presented a set of proton signals at δ_H 2.62 (2H, d J = 7.2),1.16 (1H, m), 0.95 (6H, d, J = 6.6) assignable to one isobutyl group; δ_H 3.15 (1H, m) and 1.27 (6H, d, J=7.2) for an isopropyl group; and olefinic methyl at δ_H 2.31. The DEPTQ NMR spectrum exhibited signals for five methyl groups, two methylene groups, one methyl group and one carbonyl carbon atom, as readily resolved by HSQC spectrum. The pyrazinone backbone was established on the basis of the key HMBC correlations from H-7 to C-2 and C-3, from H-15 to C-5 and C-6, and from H-11 to C-6, which also located the three side chains (via isopropyl, isobutyl moiety, and methyl group) on the backbone, respectively.

Coralinone B (**2**) was also isolated as a white crystal with chemical formula of $C_{13}H_{22}N_2O$, as determined by HRESIMS $[M+H]^+$ peak at *m/z* 223.1806 (calculated 223.1805 for $C_{13}H_{23}N_2O$). Detailed analysis of 2D NMR spectrum indicated that compound **2** was an analogue of **1**, with the subtle difference was the *sec*-butyl instead of isopropyl at C-6 in **2**. Furthermore, the planar structure of **2** was unambiguously verified by X-ray diffraction and the absolute configuration of the chiral center C-11 was *S* (Figure S2).

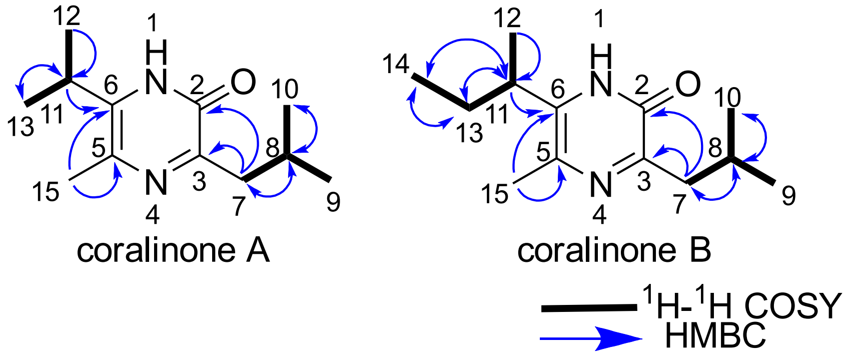

2D NMR correlations of coralinones A and B

Biomimetic total synthesis of coralinone A (1)

The characterization of the biosynthesis pathway of coralinones provoked us to carry out the biomimetic total synthesis of 5-alkylated pyrazinones and the associated biosynthetic intermediates. The total synthesis scheme commenced on the commercially available dipeptide Leu-Val-OH (**9**). After Boc protection of primary amino group, the terminal acid group of **9** was activated by carbonyldiimidazole (CDI), and then elongated with ethyl malonate potassium (EPM) to afford the Boc-protected dipeptidyl *β*-keto ester (**1a''**). The sequential deprotection of ethyl and Boc in **1a''** using NaOH and trifluoroacetic acid (TFA) drove the decarboxylation and Mannich cyclization, respectively, which mimic the biosynthetic route I in Figure 2. Conversely, treating **1a''** first with TFA and then NaOH made to mimic the biosynthetic route II. The dipeptidyl *β*-keto acid **1a'** were highly susceptible to spontaneous decarboxylation to generate **1b'**, so that LC-MS did not detect **1a'** in the reaction solution. Although **1e** was also unstable, we could detect it by LC-MS. In addition, we synthesized a dipeptidyl *β*-hydroxyl acid (**8**) primarily based on $NaBH_4$ reduction of ketone group in **1a''** for structure-activity relationship study (see below). Therefore, this organic total synthesis once again verified our proposed biosynthetic pathway for coralinones (Figure 2), wherein route I is advantagenous than route II. Actually, a plethora of research interest exist in terms of the organic synthesis of pyrazinone and its related chemical skeletons owing to the pronounced biological activities, but most reaction schemes involve tedious steps and/or harsh conditions.[3, 4] The strategy developed herein for the facile synthesis of coralinone A (**1**) is envisioned to spur a diversity of pyrazinone derivatives and/or relevant chemical skeletons.

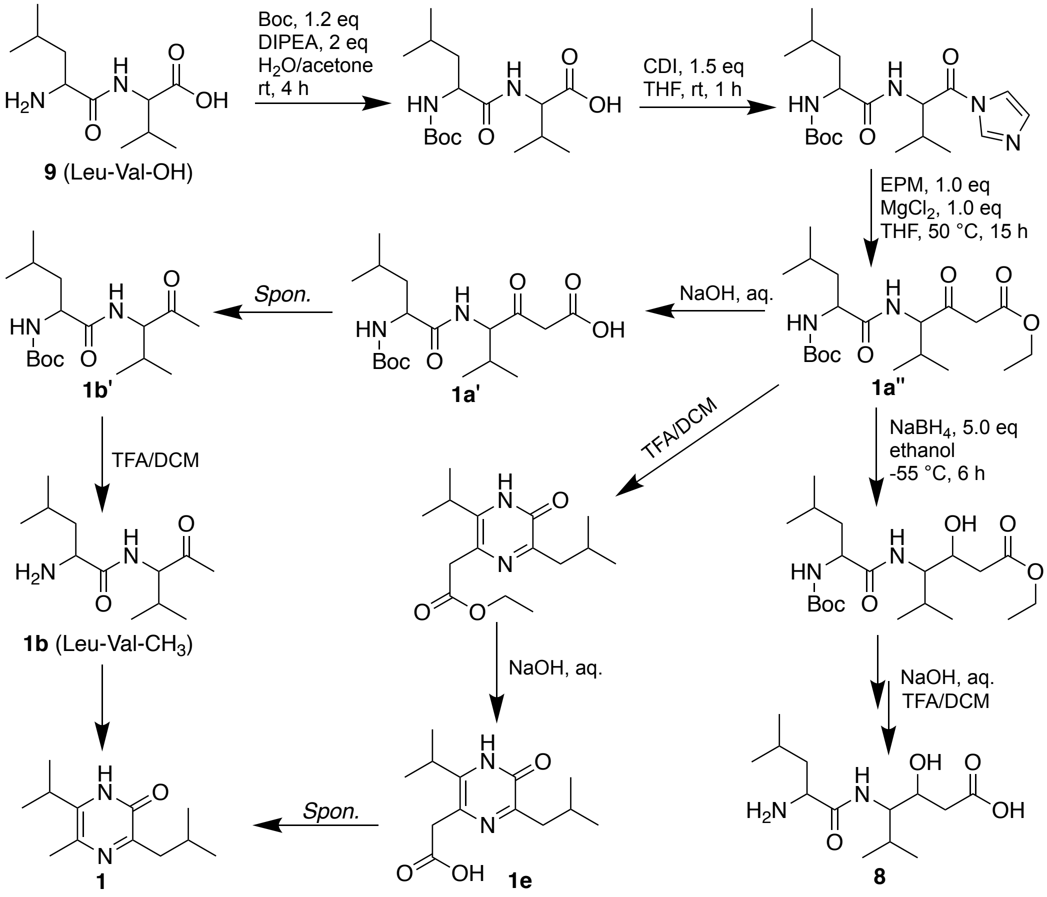

Scheme for the biomimetic total synthesis of 1. CDI, carbonyldiimidazole; EPM, ethyl malonate potassium; DIPEA, *N*, *N*-diisopropylethylamine; TFA, trifluoroacetic acid

Synthesis of Boc-Leu-Val.

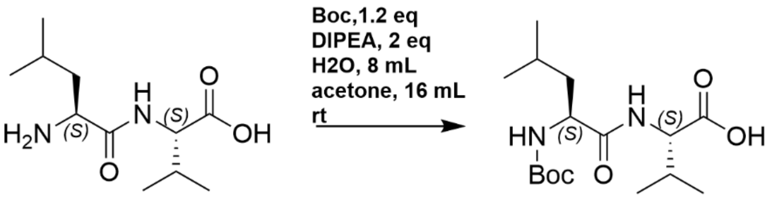

To a solution of dipeptide Leu-Val (4.35 mmol, 1 g, 1 eq) in 8 mL ddH_2O and 16 mL acetone was added DIPEA (8.7 mmol, 1.12 g, 2 eq) and Boc anhydride (5.22 mmol, 353.7 mg, 1.2 eq) at 0 °C. The reaction was heated to room temperature and stirred for 4 h. Afterwards, acetone was removed under reduced pressure. The remaining water was washed with EtoAc twice. The aqueous phase was brought to pH 2~3 by adding 1 M HCl solution and extracted three times with 30 mL of EtOAc. Then, the organic layers were combined and washed by brine, dried by anhydrous Na_2SO_4, and concentrated by vacuum evaporation to afford product (0.89 g).

Synthesis of Leu-Val-SNAC.

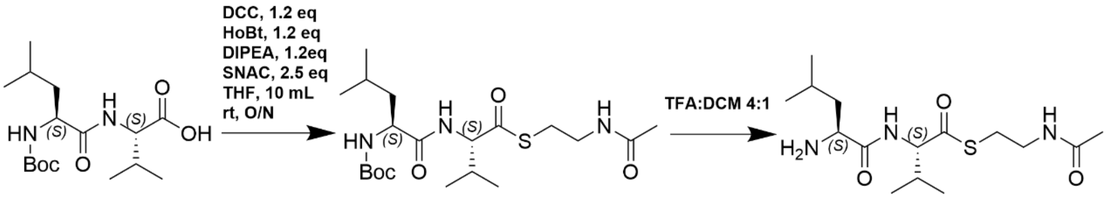

Boc-Leu-Val (1.1 mmol, 1 eq) was dissolved in THF (10 mL) in the presence of DCC (2.2 mmol, 1.2 eq), HoBt (2.2 mmol, 1.2 eq) and DIPEA. Then, SNAC (2.75 mmol, 2.5 eq) was added and allowed to react overnight at room temperature. 10 mL of saturated NH_4Cl aqueous solution was added to terminate the reaction and sodiumhydroxide solution (10% in water) was used to adjust pH 8~10. The mixture was extracted 3 times with CH_2Cl_2.The organic layer was collected, dried over Na_2SO_4 and concentrated in vacuo. Boc deprotection was carried out by directly treating with a mixture of TFA:DCM (4:1). Subsequently, preparative HPLC was performed using a C_{18} column, eluting with a gradient of 25%~45% ACN in water containing 0.1‰ TFA over 30 min. The purification process resulted in the isolation of the desired product, with a yield of 220.1 mg.^{1}H NMR (600 MHz, methanol-d_4) δ_H 4.46 (m, 1H), 4.02 (q, 1H), 3.32 (t, 2H), 3.04 (t, 2H), 2.26 (m, 1H), 1.92 (s, 3H), 1.8 (m, 2H), 1.71 (m, 1H), 1.03 (dd, 6H), 0.99 (dd, 6H). ^{13}C NMR (150 MHz, methanol-d_4) δ_C 200.7, 173.7, 172.2, 66.6, 53.2, 42.1, 40.3, 32.0, 29.3, 25.7, 23.7, 22.8, 22.0, 19.9, 18.4; HRMS-ESI (*m/z*): $[M + H]^+$ calculated for $C_{15}H_{30}N_3O_3S$, 332.2002; found 332.2006.

Synthesis of compound 1a".

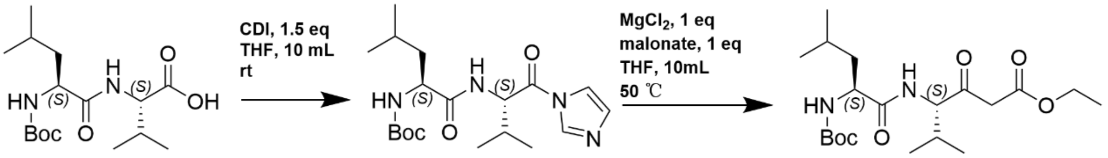

Boc-Leu-Val (2 mmol, 1 eq) and CDI (3 mmol, 1.5 eq) were added in a flask containing 10 mL of THF and stirred for 1 h at room temperature, followed by addition of $MgCl_2$ (2 mmol, 1 eq) and malonate (2 mmol, 1 eq). The reaction was continued for 15 h at 50 °C, and then cooled down to room temperature and quenched by 1 M HCl. EtOAc (3×50 mL) were used for extraction and the organic layer was separated, dried over Na_2SO_4 and concentrated in vacuo. The crude was purified on silica gel (hexanes/EtOAc 20:1) to afford the product (348.4 mg) as yellow oil. ^{1}H NMR (600 MHz, methanol-d_4) δ_H 4.91 (brs, 1H), 4.66 (m, 1H), 4.23 (m, 2H), 3.42 (s, 2H), 2.31 (m, 2H), 1.67 (m, 2H), 1.47 (m, 1H), 1.43 (s, 9H), 1.31 (t, 3H), 0.97 (s, 3H), 0.95 (d, 3H), 0.93 (d, 3H), 0.80 (d, 3H). ^{13}C NMR (150 MHz, $CDCl_3$) δ_C 203.3, 176.5, 169.0, 158.2, 81.0, 64.9, 62.6, 54.9, 42.1, 30.6, 29.0, 29.0, 29.0, 26.2, 23.7, 22.2, 20.4, 18.3, 17.9, 14.7. MS-ESI (*m/z*): $[M + Na]^+$ calculated for $C_{20}H_{36}N_2O_6Na$, 423.24; found 423.43.

Synthesis of cyclic-Leu-Val-ethyl formate.

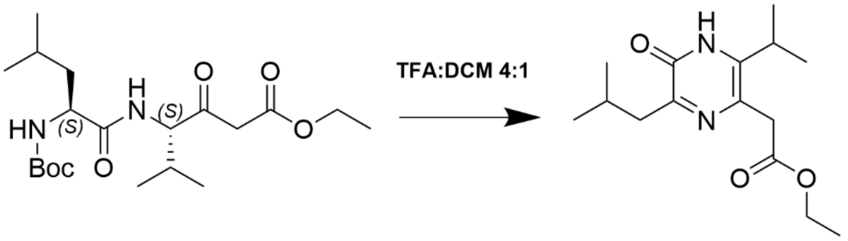

1a" was subjected to Boc-deprotection in TFA:DCM (4:1) followed by preparative HPLC eluting with 80% MeOH in water at a flow rate of 1.8 mL/min to give product.^{1}H NMR (600 MHz, methanol-d_4) δ_H 4.15 (m, 2H), 3.70 (s, 2H), 3.02 (m,1H), 2.57 (d, 2H), 2.15 (m, 1H), 1.26 (d, 6H), 1.24 (t, 3H), 0.92 (d, 6H). HRMS-ESI (*m/z*): $[M + H]^+$ calculated for $C_{15}H_{25}N_2O_3$, 281.1860; found 281.1871.

Synthesis of compound 1b'.

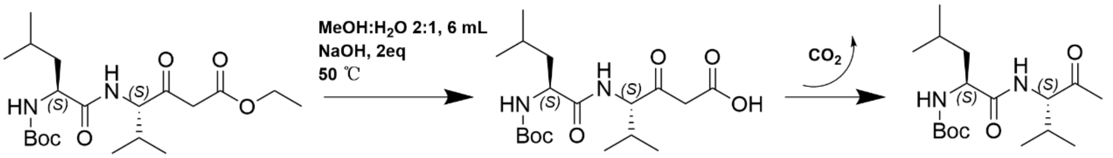

1a'' (80 mg, 1 eq) was dissolved in MeOH:H_2O (2:1, 6 mL). NaOH (0.4 mmol, 2 eq) was added and the resulting mixture was stirred at 50 °C for 10 h. The mixture was adjusted acidic using 3 M HCl. The crude was extracted with 10 mL of EtoAc 3 times and washed with water, dried over Na_2SO_4 and concentrated in vacuo. The crude was re-dissolved in MeOH and stood for 24 h. Then, the mixture was purified by preparative HPLC using a C_{18} column with a gradient of 30% to 70% MeOH in water containing 0.1‰ TFA in 30 min to give product (26.5 mg). ^{1}H NMR (600 MHz, methanol-d_4) δ_H 4.87 (brs, 1H), 4.61 (m, 1H), 2.26 (m, 1H), 2.21 (s, 3H), 1.65 (m, 2H), 1.48 (m, 1H), 1.44 (s, 9H), 0.98 (s, 3H), 0.95 (d, 3H), 0.94 (d, 3H), 0.81 (d, 3H). ^{13}C NMR (150 MHz, methanol-d_4) δ_C 208.8, 176.3, 158.2, 81.0, 65.2, 55.0, 49.9, 42.2, 31.0, 29.0, 29.0, 29.0, 26.3, 23.7, 22.2, 20.4,17.8. HRMS-ESI (*m/z*): $[M + H]^+$ calculated for $C_{17}H_{33}N_2O_4$, 329.2435; found 329.2432.

Synthesis of Boc-Leu-Val-hydroxy-methylhexanoic acid.

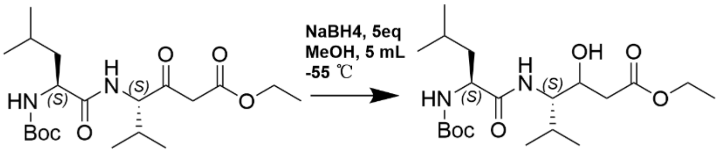

To a solution of **1a''** (0.2 mmol, 1 eq) in MeOH (5 mL) at -55 °C was added $NaBH_4$ (1 mmol, 5 eq). The reaction was stirred for 6 h and then quenched with 10% aqueous citric acid (100 mL) to pH 2~3. EtOAc (100 mL × 3) were used for extraction, and organic phases were washed with brine and dried over Na_2SO_4 and concentrated under reduced pressure. The crude extract was purified on silica gel (hexanes/EtOAc 20:1) to give product. ^{1}H NMR (600 MHz, methanol-d_4) δ_H 4.14 (m, 2H), 4.05 (t, 1H), 4.00 (td, 1H), 3.76 (dd, 1H), 2.54 (dd, 1H), 2.29 (m, 1H), 2.17 (m, 1H), 1.69 (m, 1H), 1.51 (t, 2H), 1.43 (s, 9H), 1.25 (t, 3H), 0.96 (d, 3H), 0.93 (d, 3H), 0.92 (d, 3H), 0.89 (d, 3H). ^{13}C NMR (150 MHz, methanol-d_4) δ_C 174.8, 172.4, 156.3, 79.2, 68.1, 60.2, 57.6, 53.7, 40.4, 39.4, 27.5, 27.3, 27.3, 27.3, 24.6, 19.3, 13.1. HRMS-ESI (*m/z*): $[M + Na]^+$ calculated for $C_{20}H_{38}N_2O_6Na$,425.2629; found 425.2628.

Synthesis of compound 8.

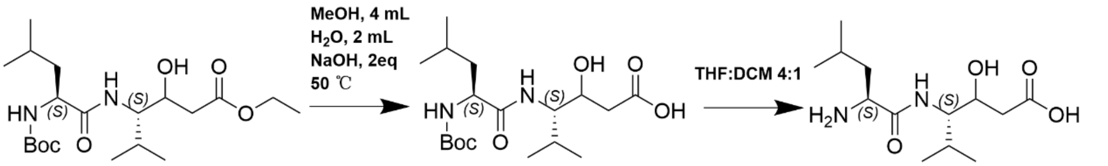

The removal of ethyl group in Boc-Leu-Val-hydroxy-methylhexanoic acid basically followed the same method for the synthesis of **1b'**, as described above. The product was then deprotected in TFA:DCM (4:1) to give **8**. ^1H NMR (600 MHz, methanol-d_4) δ_H 4.06 (t, 2H), 3.99 (t, 1H), 3.73 (m, 1H), 2.49 (m, 1H), 2.45 (m, 1H), 1.67 (m, 1H), 1.51 (m, 2H), 1.44 (s, 9H), 1.01 (d, 2H), 0.98 (d, 6H), 0.89 (d, 3H). ^{13}C NMR (150 MHz, methanol-d_4) δ_C 176.6, 176.5, 158.2, 81.0, 69.8, 59.3, 55.3, 42.1, 41.0, 29.3, 29.0, 16.3, 23.8, 22.3, 21.0, 16.5. HRMS-ESI (*m/z*): $[M + H]^+$ calculated for $C_{13}H_{27}N_2O_4$,275.1965; found 275.1968.

Synthesis of 10b'.

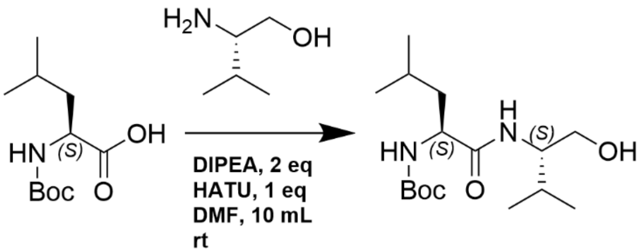

Boc-Leu-OH (1 mmol, 1 eq) and 2-amino-3-methylbutan-1-ol (1 mmol, 1 eq) were dissolved in DMF (10 mL). Then, DIPEA (2 mmol, 2 eq) and HATU (1 mmol,31 eq) were added. The reaction was stirred for 3 h at room temperature. 50 mL of ddH_2O was added to quench the reaction and the mixture was extracted with EtOAc (20 mL × 3). The organic layer was collected, washed with brine, dried by anhydrous Na_2SO_4, and concentrated by rotary evaporation. The resulting residue was used for the next step without purification.

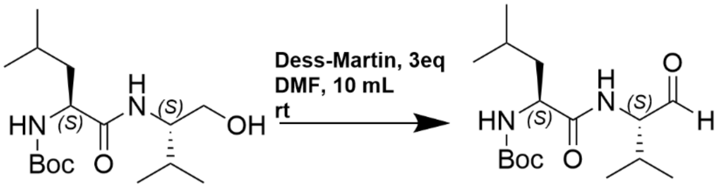

Boc-Leu-Val-OH (1 mmol, 1 eq) and Dess-Martin periodinane (3 mmol, 3 eq) were dissolved in DMF (10 mL). The reaction was stirred for 5 h at rt. 50 mL of ddH_2O was added to quench the reaction and the mixture was extracted with EtOAc (20 mL × 4). The organic layer was collected, washed with brine, dried by anhydrous Na_2SO_4, and concentrated by rotary evaporation. The crude extract was purified by semi-preparative HPLC eluting with 55% MeOH in water at a flow rate of 1.8 mL/min to give Boc-Leu-Val-H (29.8 mg). ^{1}H NMR (600 MHz, methanol-*d*$_4$) δ_H 9.64 (s, 1H), 4.85 (brs, 1H), 4.53 (m, 1H), 2.33 (m, 1H), 1.69 (m, 2H), 1.45 (s, 9H), 1.01 (d, 3H), 0.97 (d, 3H), 0.96 (d, 3H), 0.94 (d, 3H). HRMS: $[M + H]^+$ calculated for $C_{16}H_{31}N_2O_4$,315.2278; found 315.2270.

Synthesis of 10.

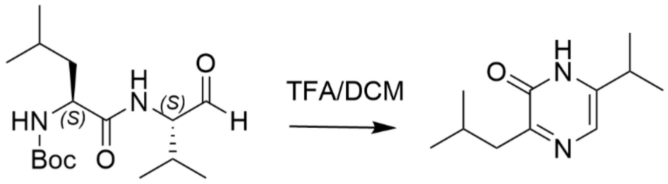

Boc-Leu-val-H was deprotected in TFA: DCM (4:1), and **10** was formed spontaneously. The target compound was purified by preparative HPLC eluting with 80% MeOH in water at a flow rate of 1.8 mL/min. ^{1}H NMR (600 MHz, methanol-*d*$_4$) δ_H 7.17(s, 1H), 2.79 (m, 1H), 2.58 (d, 2H), 2.15 (m, 1H), 1.28 (d, 6H), 0.94 (d, 6H).

Supplementary tables

Table S1. Annotations for *cor* gene cluster (GeneBank Accession NO. SAMN27682953)**.**

ORF	Gene size(bp)	Gene name	NCBI similarity	ID%
corB	1515	ctg1_4398	Do family serine endopeptidase [*Corallococcus sp.* AB011P]; WP_120562136.1	99%
2	459	ctg1_4397	phosphorelay signal transduction system [*Corallococcus coralloides*]; WP_128794817.1	99%
3	804	ctg1_4396	glutaminyl-peptide cyclotransferase activity [*Corallococcus aberystwythensis*]; WP_120554135.1	99%
4	537	ctg1_4395	metal-dependent hydrolase [*Corallococcus coralloides*]; WP_171422663.1	99%
corA	8604	ctg1_4394	non-ribosomal peptide synthetase [*Corallococcus coralloides*]; WP_171422662.1	96%
6	450	ctg1_4393	nuclear transport factor 2 family protein [*Corallococcus coralloides*); WP_171422661.1	97%
7	234	ctg1_4392	hypothetical protein [*Corallococcus sp.* AB038B]; WP_120593232.1	96%
8	1092	ctg1_4391	phosphoric diester hydrolase activity [*Corallococcus sp.* CA054B]; WP_120590857.1	93%
9	507	ctg1_4390	MarR family transcriptional regulator [*Corallococcus sp.* NCSPR001]; WP_206799689.1	99%
10	1284	ctg1_4389	HlyD family secretion protein [*Corallococcus sp.* CA049B]; WP_120621535.1	99%
11	1551	ctg1_4388	DHA2 family efflux MFS transporter permease subunit [*Corallococcus sp.* AB011P]; WP_233591435.1	97%

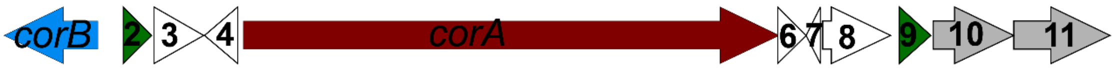

Table S2. Cytotoxic activity (IC_{50} in μM) for coralinones A (1) and B (2) on a panel of human cell lines. The presented data is the mean value of three replicates.

**Cell lines**	**1**	**2**	Docetaxel
Human lung carcinoma A549	383.44	145.31	33.14
Human hepatocarcinoma HepG2	892.22	503.87	10.32
Human prostatic carcinoma PC3	796.15	432.10	71.85
Human esophageal squamous carcinoma KYSE410	463.09	312.23	17.78

Table S3. Plasmids used in this study

Vectors and plasmids	Description[a]	Source/references
pET28a	E. coli expression vector, ColEl ori, Km^r	Novagen
pET28a-*corA*	pET28a derivative containing *corA* from *Corallococcus exiguus* SDU70, Km^r	This study
pET28a-*corB*	pET28a derivative containing *corB* from *Corallococcus exiguus* SDU70, Km^r	This study
pET28a-*corA*-PKS	Ligation of the PKS portion of the *corA* module to pET28a, Km^r	This study
pSWU19	Site specific integration vector with Mx8 attB integration site (Mx8); Km^r	(Wu and Kaiser 1995)[5]
pSWU19-*corA*	Ligating *corA* to the pSWU19, Km^r	This study
pSWU19-*corB*	Ligating *corB* to the pSWU19, Km^r	This study
pSWU19-*corAB*	Ligating *corA* and *corB* to the pSWU19, Km^r	This study
pBJ113	Gene replacement vector with KG cassette; Used to create insertiondeletions, GalK, Km^r	(Yang, Virginia Tech)[6]
pBJ113-△*corA*	Ligating the one homologous arm of *corA* for insertion to the pBJ113, Km^r	This study
pBJ113-*Ptn5-corA*	pBJ113 with a strong promoter *Ptn5* and homologous sequence of *corA*, Km^r	This study
pBJ113-*PJ23104-corB*	pBJ113 with a strong promoter *PJ23104* and homologous sequence of *corB*, Km^r	This study
pBJ113-△*corA-PJ23104-corB*	pBJ113 with one homologous arm of *corA*, strong promoter *PJ23104* and homologous sequence of *corB*, Km^r	This study

[a] Km^r, resistance to kanamycin.

Table S4. Strains used in this study.

Organisms/Strains	Description	Source/references
Escherichia coli DH5α	F φ80*lacZ*ΔM15 Δ(*lacZYA-argF*) U169 *recA1 endA1 hsdR17(*rk^- mk^+ *phoA*Δ*supE44 thi-1 gyrA96 relA1* λ-	Life Technologies
Escherichia coli BAP1	BL21(DE3) Δ*prpRBCD :: PT7-sfp-PT7-prpE*	(Pfeifer and Admiraal 2001)[7]
Corallococcus exiguus SDU70	Separated from campus soil.	This Lab
Myxococcus xanthus DK1622	Separated from campus soil.	This Lab
M. xanthus DK1622-*corA*	*Myxococcus xanthus* DK1622 with integration of pSWU19-*corA*, to heterogeneously express *corA.*	This study
M. xanthus DK1622-*corB*	*Myxococcus xanthus* DK1622 with integration of pSWU19-*corB*, to heterogeneously express *corB.*	This study
M. xanthus DK1622-*corAB*	*Myxococcus xanthus* DK1622 with integration of pSWU19-*corAB*, to heterogeneously express *corAB.*	This study
M. xanthus DK1622-pSWU19	*Myxococcus xanthus* DK1622 with integration of pSWU19, for experimental control.	This study
C. exiguus SDU70-pBJ113	*Corallococcus exiguus* SDU70 with integration of pBJ113, for experimental control.	This study
C. exiguus SDU70-△*corA*	*Corallococcus exiguus* SDU70 with integration of pBJ113-△*corA*, to knock out the *corA* of the original strain.	This study
C. exiguus SDU70-*corA*	*Corallococcus exiguus* SDU70 with integration of pBJ113-*Ptn5,* to overexpress the *corA* of the source strain.	This study
C. exiguus SDU70-*corB*	*Corallococcus exiguus* SDU70 with integration of pBJ113-*PJ23104,* to overexpress the *corB* of the source strain.	This study
C. exiguus SDU70-△*corA-corB*	*Corallococcus exiguus* SDU70 with integration of pBJ113-△*corA*, the *corB* gene was overexpressed while the *corA* gene was knocked out in the original strain.	This study

Table S5. Primers used in this study. (Normal fonts: homologous arms；Bold fonts: priming sequence；Underlined fonts: restriction site)

Primer	Sequence（5’ → 3’）	Function
pET28a-Fw	**GAATTCGAGCTCCGTCGACAAG**	PCR linearize the plasmid pET28a
pET28a-Rv	**ATGGCTGCCGCGCGGCACC**	
corA-28HA-Fw	TGCCGCGCGGCAGCCAT**ATGGAATTCCATCTCCTCGCACCC**	cloning *corA* into plasmid pET28a for protein expression
corA-28HA-Rv	GTCGACGGAGCTCGAATTC**TCATCCCGCGTCGAGGTCATC**	
corA-PKS-Fw	TGCCGCGCGGCAGCCAT**ATGTCCATCGCCATCATCGGCAC**	cloning *corA-pks* into plasmid pET28a for protein expression
corA-PKS-Rv	GTCGACGGAGCTCGAATTC**TCATCCCGCGTCGAGGTCATC**	
corB-28HA-Fw	CTGGTGCCGCGCGGCAGCCAT**ATGGTCCGCACGCTTCCGTC**	cloning *corB* into plasmid pET28a for protein expression
corB-28HA-Rv	CCAGCGGCGCCAGGCTCAT**GCCGCTGCTGTGATGATGATG**	
corA-19HA-Fw	GACGGCCAGTGCCAAGCTT**TCATCCCGCGTCGAGGTCATC**	cloning *corA* into plasmid pSWU19 for heterologous expression in DK1622
corA-19HA-Rv	ACCATGATTACGAATTC**ATGAATTCCATCTCCTCGCACCC**	
corB-19HA-Fw	CGACGGCCAGTGCCAAGCTT**ATGGTCCGCACGCTTCCGTC**	cloning *corB* into plasmid pSWU19 for heterologous expression in DK1622
corB-19HA-Rv	GCTATGACCATGATTACGAATTC**CTACCGCTGCGGCACTT**	
pSWU19-Fw	**GAATTCGTAATCATGGTCATAGCTG**	PCR linearize the plasmid pSWU19
pSWU19-Rv	**AAGCTTGGCACTGGCCGTCG**	
corB-Fw	**ATGAGCCTGGCGCCGCTGG**	cloning *corB* into plasmid pSWU19-*corA* for co-expression *corB* and *corA* in DK1622
corB-Rv	**CTACCGCTGCGGCACTTCAA**	
pSWU19-*corAB*-HA-Fw	CTTGAAGTGCCGCAGCGGTAG**AGCTTGGCACTGGCCGTCG**	PCR linearize plasmid pSWU19-*corA.*
pSWU19-*corAB*-HA-Rv	CGGAAGCGTGCGGACCATT**TCATCCCGCGTCGAGGTCATC**	
corA-pBJ-HA-Fw	GAGCTCGGTACCCGGGGATCC**CTGGAACAGCGTGGCCATG**	amplification of homologous arm for insertional inactivation of *corA*
corA-pBJ-HA-Rv	GCCTGCAGGTCGACTCTAGA**CTGGCGGACTTCCTGGAGAC**	
pBJ113-Fw	**TCTAGAGTCGACCTGCAGGC**	PCR linearize plasmid pBJ113
pBJ113-Rv	**GGATCCCCGGGTACCGAGC**	
△*corA*-check-F1	**GTCTGCACCATCCGTTCGAAG**	PCR verification for insertional inactivation of *corA* in SDU70
△*corA*-check-R1	**GGTAACTGGCTTCAGCAGAG**	
△*corA*-check-F2	**CCCACTGCAAGCTACCTGC**	PCR verification for insertional inactivation of *corA* in SDU70
△*corA*-check-R2	**GCGAAGAGGGAGATGCCTC**	
Ptn5-HA-Fw	AGCTCGGTACCCGGGGATCC**TCACGCTGCCGCAAGCACTC**	amplification of promotor Tn5 for the overexpress of *corA* in SDU70
Ptn5-HA-Rv	CCACCAGTCATGCTAGCCAT**GCGAAACGATCCTCATCCTG**	
pBJ113-*Ptn5-corA*-HA-Fw	CAGGATGAGGATCGTTTCGC**ATGGCTAGCATGACTGGTGG**	overlapping PCR ligation of *corA* and promotor Tn5
pBJ113-*Ptn5-corA*-HA-Rv	GCCTGCAGGTCGACTCTAGA**GGACATGCAGCTGGAGCTTC**	
corA-check-F	**GACCGAAATCGGCAAAATCCC**	PCR verification for overexpression of *corA* in SDU70
corA-check-R	**GGCATCGAAGGCATACGACG**	
corB-*J23104*-HA-Fw	TTGACAGCTAGCTCAGTCCTAGGTATTGTGCTAGCGGAATTGTGAGCGGATAACAATTCCCCTCTAGAAATAATTTTGTTTAACTTTAAGAAGGAGATATCAT**ATGGTCCGCACGCTTCCGTC**	overexpression of *corB* in SDU70
corB-Rv	**CTACCGCTGCGGCACTTCAA**	
pBJ113-*corB*-HA-Fw	TTGAAGTGCCGCAGCGGTAG**GAATTCCTTATTGCCCGACG**	concurrent overexpression of *corB* and insertional inactivation of *corA* in SDU70
pBJ113-*corB*-HA-Rv	GCTAGCACAATACCTAGGACTGAGCTAGCTGTCAA**AAGCTTTCTGCCTCGCGCG**	
corB-check-F	**GCTCTCCGAGCAATTCCGTC**	PCR verification for overexpression of *corB.*
corB-check-R	**GTTGGTAGCTCTTGATCCGG**	
△*corA-corB*-check-F	**GAGGTGAAGGTGAGCCTGGTG**	PCR verification for concurrent overexpression of *corB* and insertional inactivation of *corA* in SDU70
△*corA-corB*-check-R	**CAGCTCGATGCGGAAGCC**	
corA-RT-Fw	**GCAACGCGGCCTCAATCTC**	RT-PCR of *corA* in the SDU70
corA-RT-Rv	**CTGCTGGATGAAACCCTGAC**	
corB-RT-Fw	**GTTCGTGAAGCTGGGGGATTC**	RT-PCR of *corB* in the SDU70
corB-RT-Rv	**CGATGATGGCCGTGTTCATC**	

Table S6. X-ray crystallographic data for coralinone B.

Empirical formula	$C_{13}H_{22}N_2O$
Formula weight	222.32
Temperature/K	99.99(10)
Crystal system	monoclinic
Space group	C2
a/Å	16.19520(10)
b/Å	19.58410(10)
c/Å	26.1333(2)
α/°	90
β/°	103.2980(10)
γ/°	90
Volume/Å^3	8066.41(10)
Z	24
ρ_{calc}g/cm^3	1.098
μ/mm^{-1}	0.545
F (000)	2928.0
Crystal size/mm^3	0.2 × 0.15 × 0.1
Radiation	Cu Kα (λ = 1.54178)
2Θ range for data collection/°	6.952 to 148.882
Index ranges	-20 ≤ h ≤ 19, -24 ≤ k ≤ 23, -32 ≤ l ≤ 32
Reflections collected	74435
Independent reflections	15798 [R_{int} = 0.0365, R_{sigma} = 0.0237]
Data/restraints/parameters	15798/22/977
Goodness-of-fit on F^2	1.103
Final R indexes [I>=2σ (I)]	R_1 = 0.0528, wR_2 = 0.1571
Final R indexes [all data]	R_1 = 0.0567, wR_2 = 0.1584
Largest diff. peak/hole / e Å^{-3}	0.43/-0.28
Flack parameter	0.00(8)

Table S7. ^{1}H and ^{13}C NMR data of coralinones A (1) and B (2) in methanol-d_4.

Pos.	**1**		**2**	
	δ_C, type	δ_H, mult. (J in Hz)	δ_C, type	δ_H, mult. (J in Hz)
1	NH	-	NH	-
2	C	158.8	C	158.7
3	C	153.5	C	153.5
4	N	-	N	-
5	C	130.1	C	130.1
6	C	126.2	C	126.3
7	42.3, CH_2	2.62, d (7.2)	42.6, CH_2	2.58, d (7.2)
8	28.7, CH	2.16, m	28.6, CH	2.15, m
9	23.2, CH_3	0.95, d (6.6)	23.2, CH_3	0.93, d (6.6)
10	23.2, CH_3	0.95, d (6.6)	23.2, CH_3	0.93, d (6.6)
11	30.4, CH	3.15, m	37.4, CH	2.84, m
12	20.9, CH_3	1.27, d (7.2)	19.0, CH_3	1.25, d (6.6)
13	20.9, CH_3	1.27, d (7.2)	29.3, CH_2	1.66, m
14			12.6, CH_3	0.86, t (7.2)
15	18.5, CH_3	2.31 s	19.1, CH_3	2.31, s

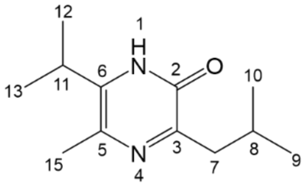

1

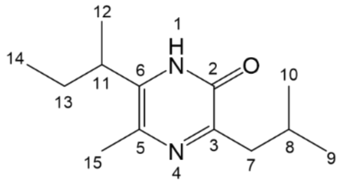

2

Supplementary graphs

Figure S1. HPLC profiling of SDU70 crude extract. The characteristic UV absorption spectra for compounds **1** and **2** were provided.

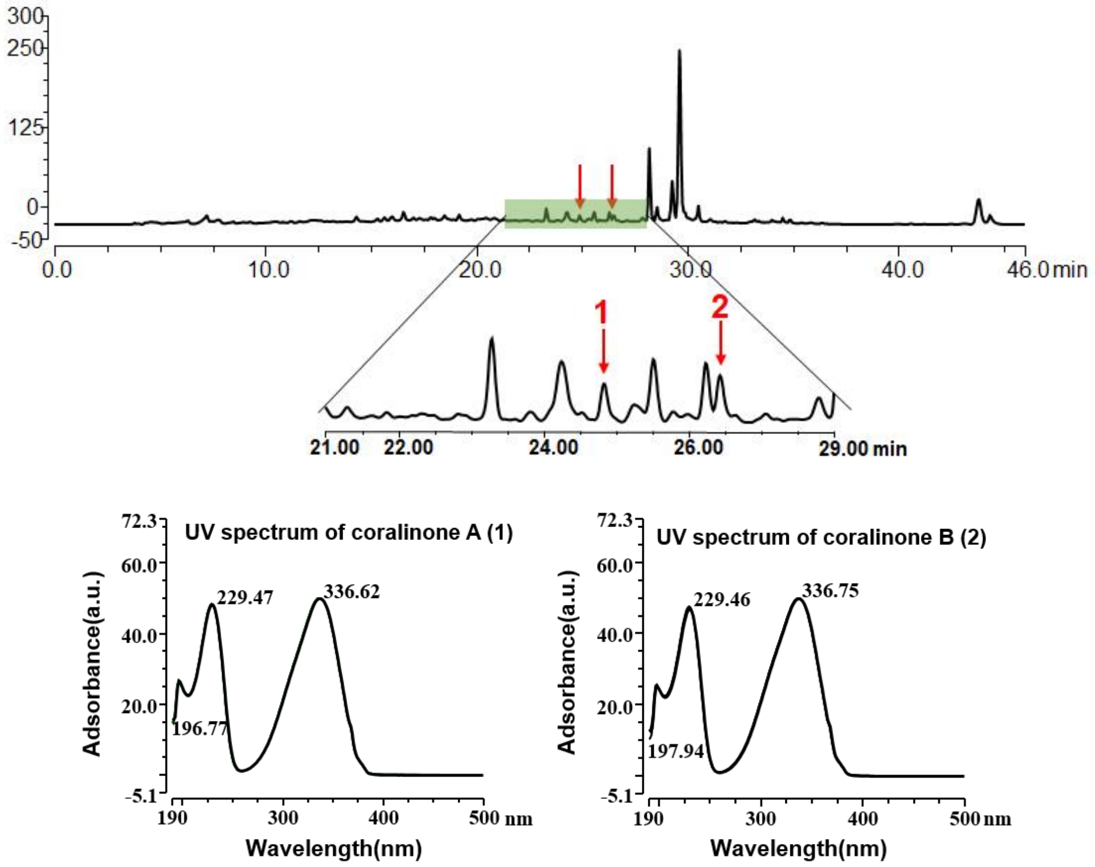

Figure S2. ORTEP diagram of the crystal structure of coralinone B (2). The absolute configuration of isobutyl group is *S*.

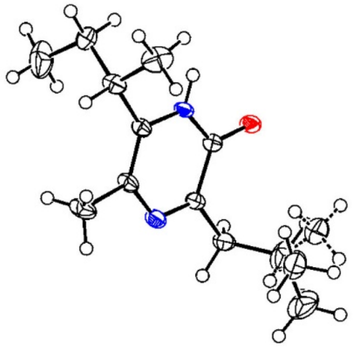

Figure S3. Isotope feeding experiment with L-leucine d_3 and L-isoleucine d_{10}. (**A**) Comparison of MS spectra of L-leucine d_3-labelled (red) and non-labelled (black) coralinone A. (**B**) Comparison of MS spectra of L-leucine d_3-labelled coralinone B (red) and natural abundance coralinone B (black). (**C**) Comparison of MS spectra of L-isoleucine d_{10}-labelled coralinone B (down) and natural coralinone B (up).

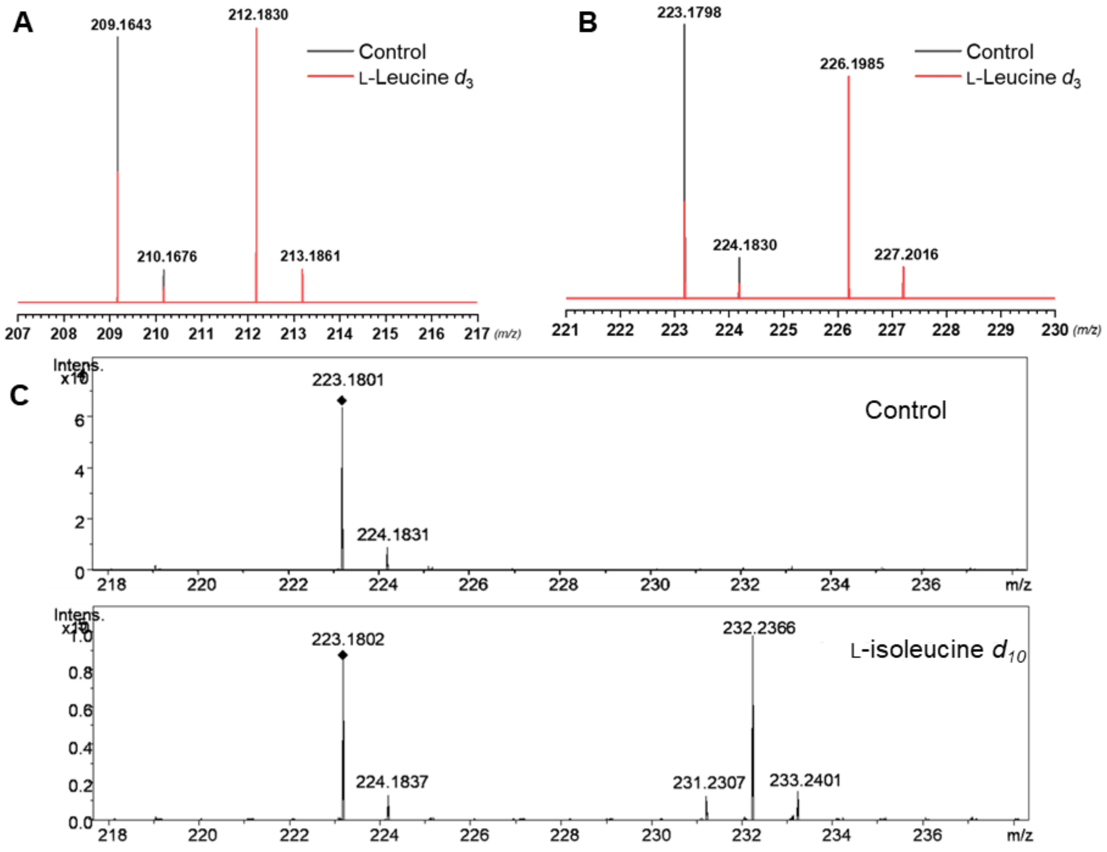

Figure S4. SDS-PAGE analysis of recombinant proteins CorA, CorA-PKS, and CorB. M: molecular mass standards. CorA: 310.7 kDa; CorA-PKS: 131.5 kDa; CorB: 45.6 kDa.

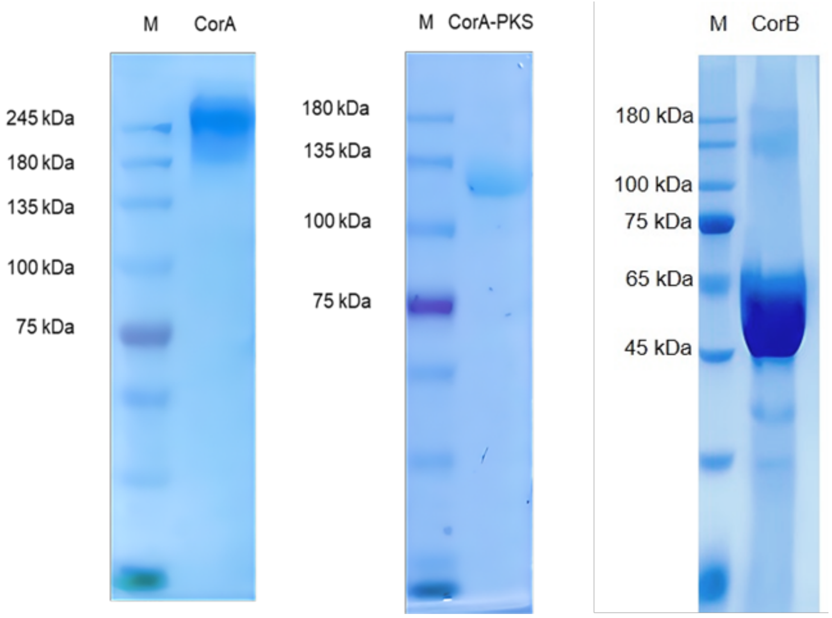

Figure S5. Substrate preference of the two A domains in CorA. (**A**) Tabular summarization of pairwise combination of amino acid substrates for SFP-activated *holo*-CorA, while keeping Mal-CoA as a constant substrate. The three amino acids Leu, Ile, or Val was individually coupled with the other 19 proteinogenic amino acids. (**B**) Structures of coralinones A–F (**1**–**6**) generated by **combinational enzymatic synthesis**, all of which are new compounds. Compounds identification was done on the basis of high-resolution mass (Figure S6) and the characteristic UV spectra (Figure S1).

(A)

	Asp	Ser	Glu	Gly	His	Arg	Thr	Ala	Pro	Cys	Try	Val	Met	Lys	Ile	Leu	Phe	Trp	Gln	Asn
**Leu**												2			1					
**Ile**													4			2	6			
**Val**													3			1	5			

(B)

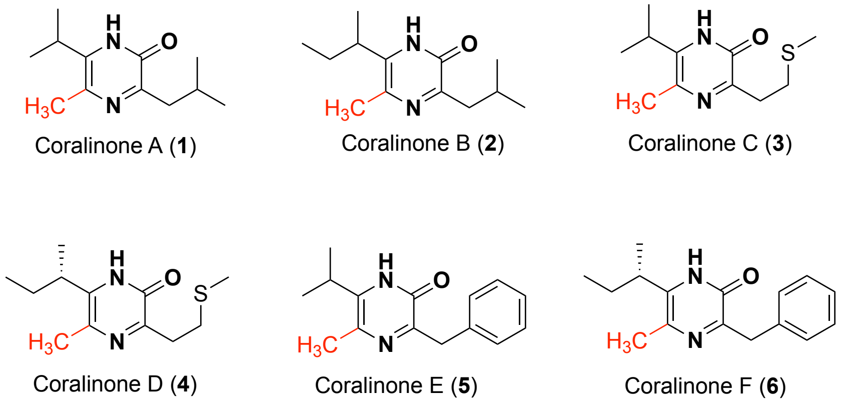

Figure S6. HR-ESI-MS data of the enzymatic products 1–7.

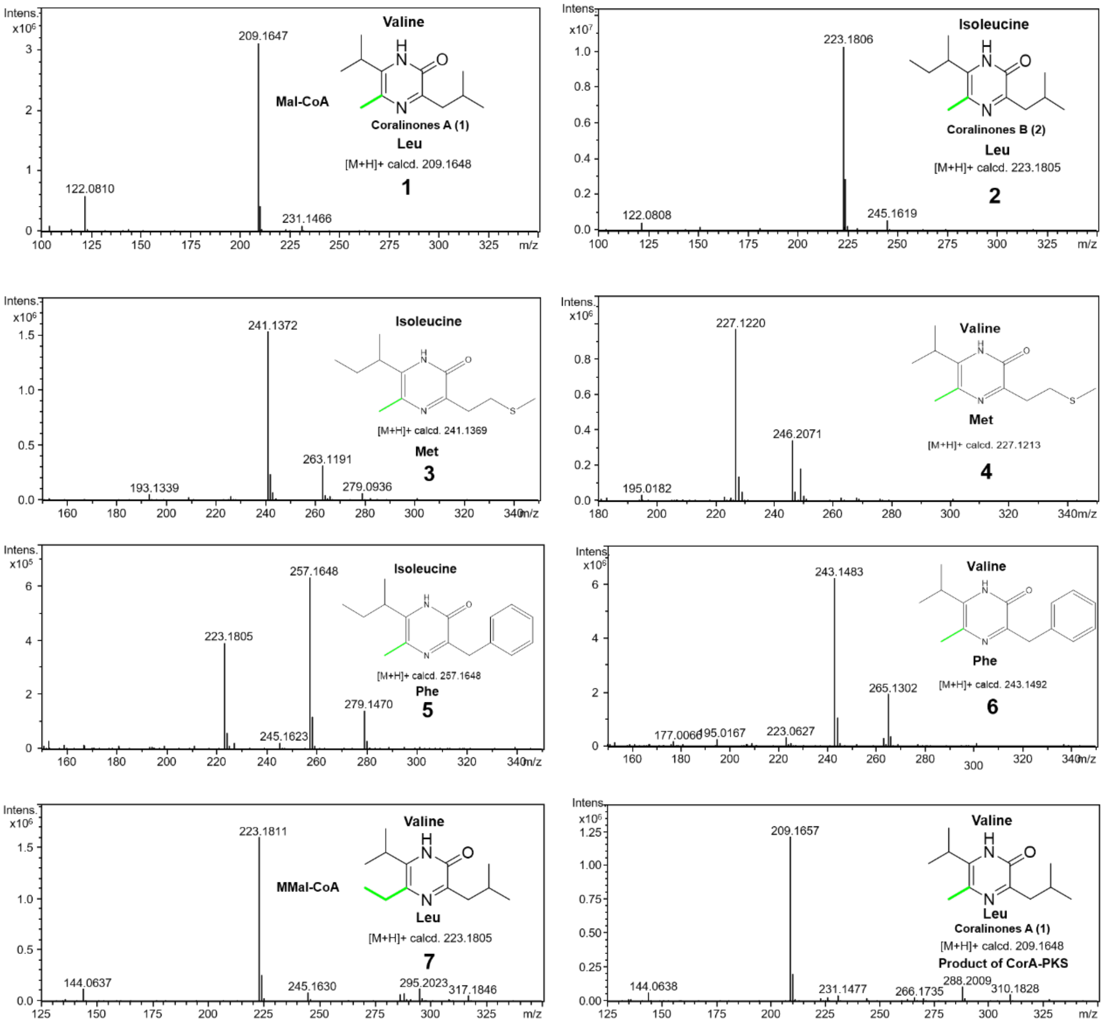

Figure S7. The relative yield of compounds 1–6 produced by CorA during *in vitro* enzymatic assays. (**A**) The amount of each compound (**1**, **3–5**) was proportionated to the amount of the most abundant compound **2**. Each experiment was done in triplicate. (**B**) Substrate preference of the first A domain in CorA, as tested by the ATP–PPi exchange assay. The relative activity of A1 domain for the activation of various amino acids. The activity of A1 for activation of L-Leu was quantified as a reference (100%). Data are presented as mean values ±SD, n=3 biologically independent samples.

(A)

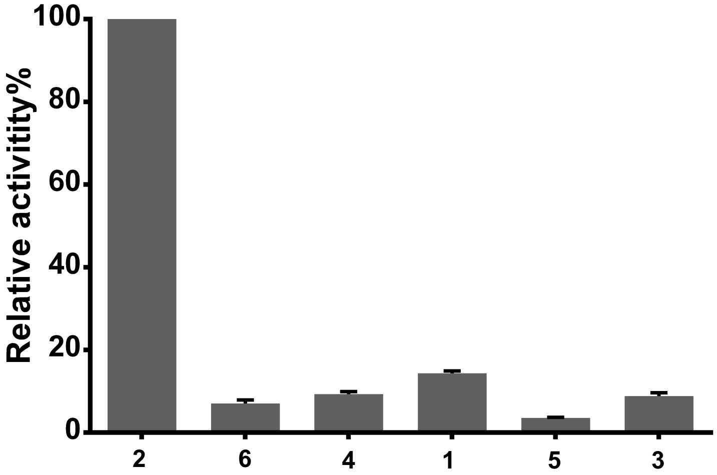

(B)

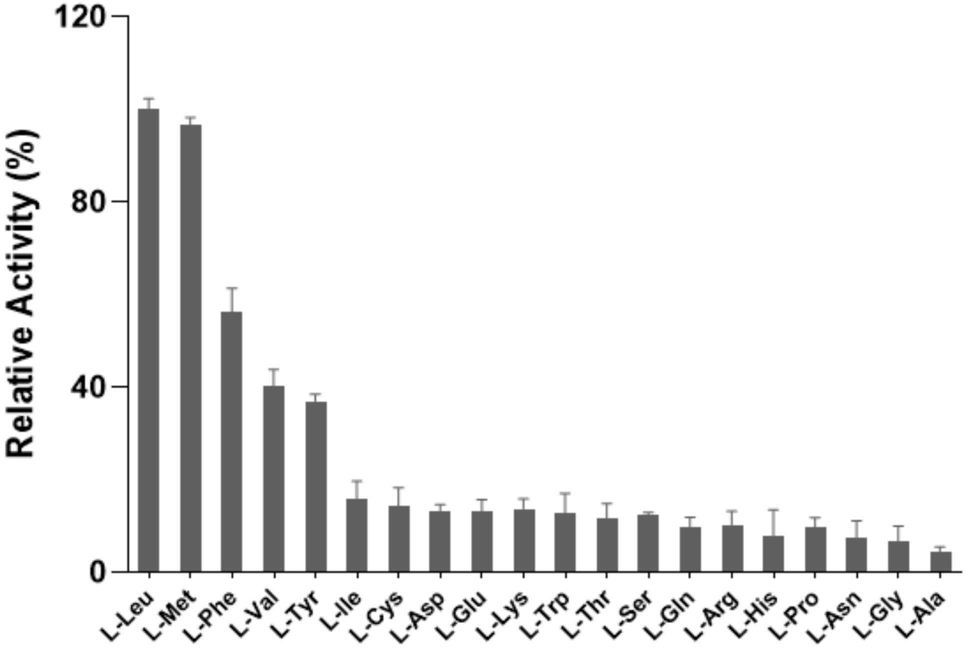

Figure S8. Metabolic analysis verified the fidelity of genotypes after genetic manipulation of SDU70 and DK1622. HPLC-DAD profiling (detected at 280 nm) demonstrated that the yield of coralinones was enhanced in the *corA*-overexpression mutant SDU70-*corA* and abolished in the knockout mutant SDU70-*∆corA*. Heterologous expression of *corA* in DK1622 (DK1622-*corA*) led to the production of coralinones, whilst not in control strain DK1622-vector with empty vector pSWU19.

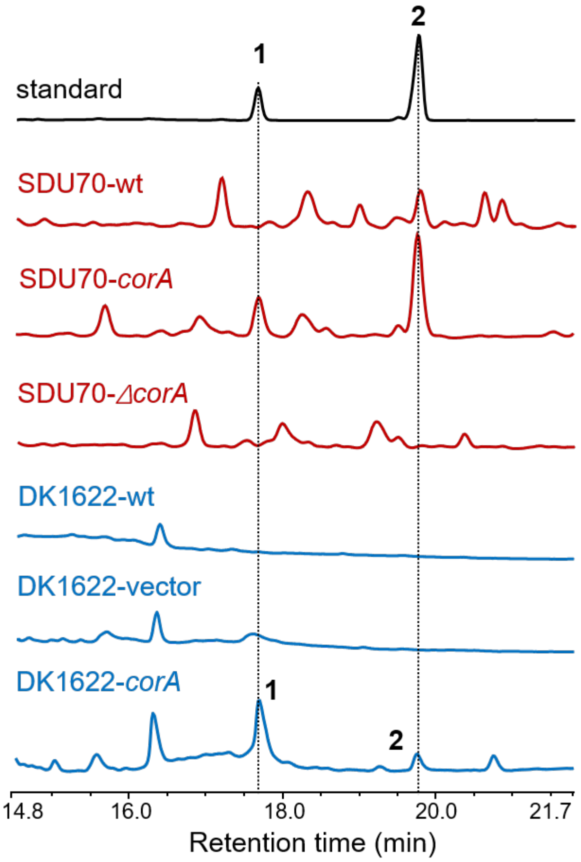

Figure S9. Compounds 1 and 1b' induced the agglutination of strains SDU70-*⊿corA* and DK1622-wt in a dose-dependent manner. The tested compounds concentration was 1~50 mg/L.

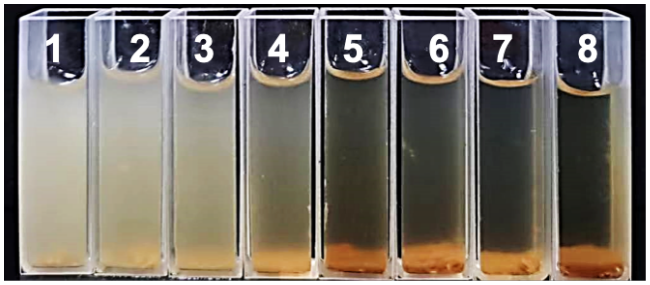

1：SDU70-*⊿corA*
2：SDU70-*⊿corA* +1 mg/L of **1**
3：SDU70-*⊿corA* +2 mg/L of **1**
4：SDU70-*⊿corA* +5 mg/L of **1**
5：SDU70-*⊿corA* +10 mg/L of **1**
6：SDU70-*⊿corA* +15 mg/L of **1**
7：SDU70-*⊿corA* +20 mg/L of **1**
8：SDU70-*⊿corA* +50 mg/L of **1**

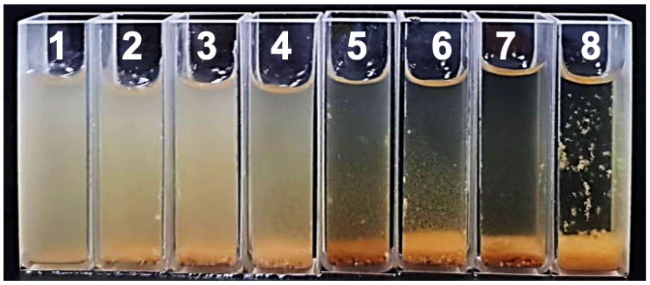

1：SDU70-*⊿corA*
2：SDU70-*⊿corA* +1 mg/L of **1b'**
3：SDU70-*⊿corA* +2 mg/L of **1b'**
4：SDU70-*⊿corA* +5 mg/L of **1b'**
5：SDU70-*⊿corA* +10 mg/L of **1b'**
6：SDU70-*⊿corA* +15 mg/L of **1b'**
7：SDU70-*⊿corA* +20 mg/L of **1b'**
8：SDU70-*⊿corA* +50 mg/L of **1b'**

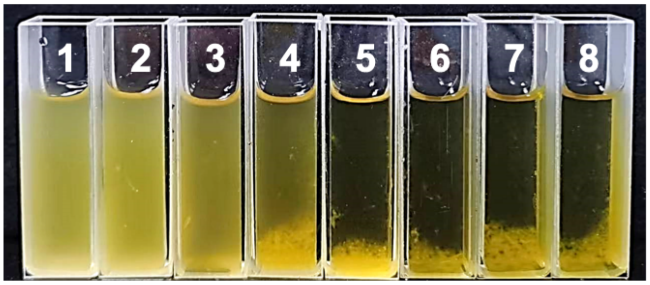

1：DK1622-wt
2：DK1622+1 mg/L of **1**
3：DK1622+2 mg/L of **1**
4：DK1622+5 mg/L of **1**
5：DK1622+10 mg/L of **1**
6：DK1622+15 mg/L of **1**
7：DK1622+20 mg/L of **1**
8：DK1622+50 mg/L of **1**

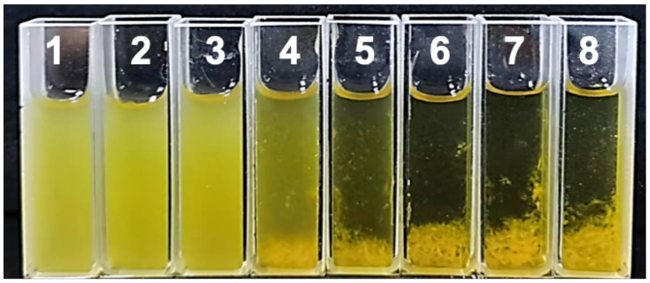

1：DK1622-wt
2：DK1622+1 mg/L of **1b'**
3：DK1622+2 mg/L of **1b'**
4：DK1622+5 mg/L of **1b'**
5：DK1622+10 mg/L of **1b'**
6：DK1622+15 mg/L of **1b'**
7：DK1622+20 mg/L of **1b'**
8：DK1622+50 mg/L of **1b'**

Figure S10. Agglutination assay of the biosynthetically related compounds in relation to 5-unsubstituted and 5-methylated pyrazinones. Each compound was added at 5 mg/L. The white arrows denote the settling of agglutinated cell clumps.

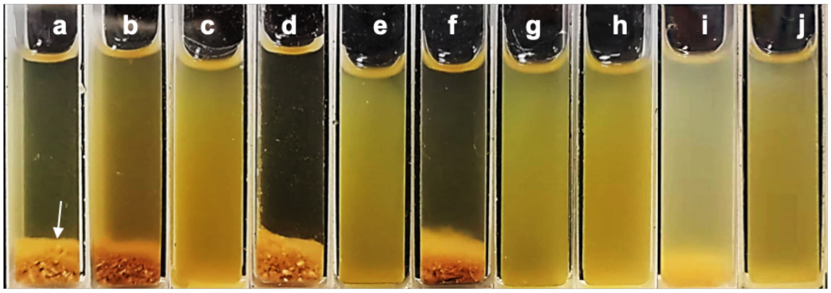

a, SDU70-wt
b, SDU70-*corA*
c, SDU70-△*corA*
d, SDU70-△*corA* + **1**
e, SDU70-△*corA* + **9**
f, SDU70-△*corA* + **1b'**
g, SDU70-△*corA* + **8**
h, SDU70-△*corA* + **1a''**
i, SDU70-△*corA* + **10**
j, SDU70-△*corA* + **10b'**

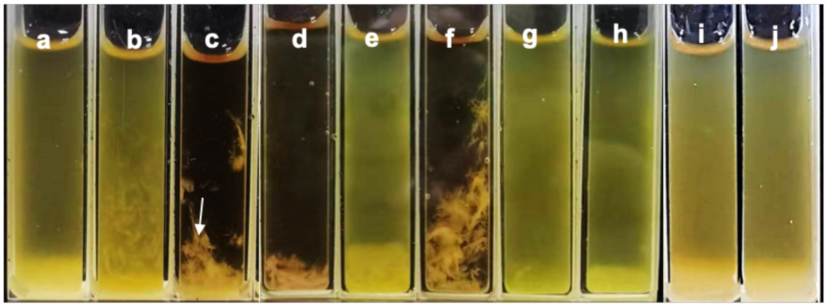

a, DK1622-wt
b, DK1622-vector
c, DK1622-*corA*
d, DK1622-wt + **1**
e, DK1622-wt + **9**
f, DK1622-wt + **1b'**
g, DK1622-wt + **8**
h, DK1622-wt + **1a''**
i, DK1622-wt + **10**
j, DK1622-wt + **10b'**

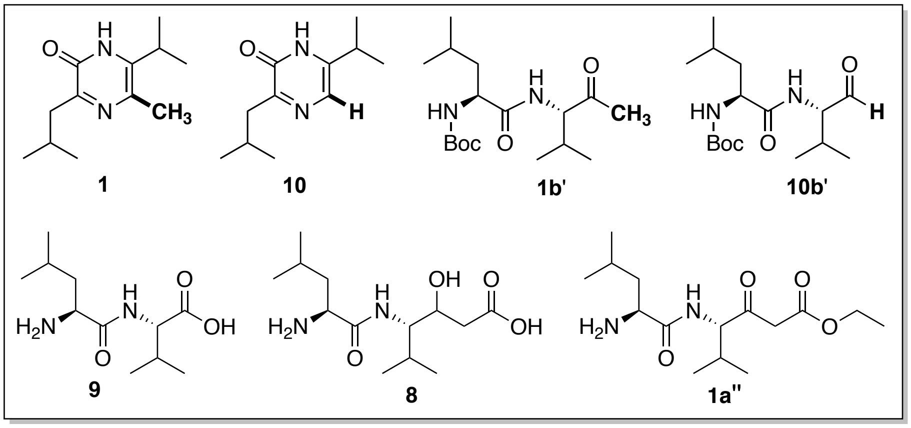

Figure S11. Biotransformation of 1b' by the strains SDU70-*△corA* and DK1622-wt. The cultures of SDU70-*△corA* or DK1622-wt were supplemented with **1b'** just prior to inoculation. After 3 days of growth, the presence of **1b'** and/or **1** in the cultures was monitored by HPLC-DAD detected at 280 nm (**A**). The compounds identification was done on the basis of and the characteristic UV spectra of **1b'** and **1** (**B**). The strains SDU70-*△corA* and DK166-wt did not converted **1b'** into **1**.

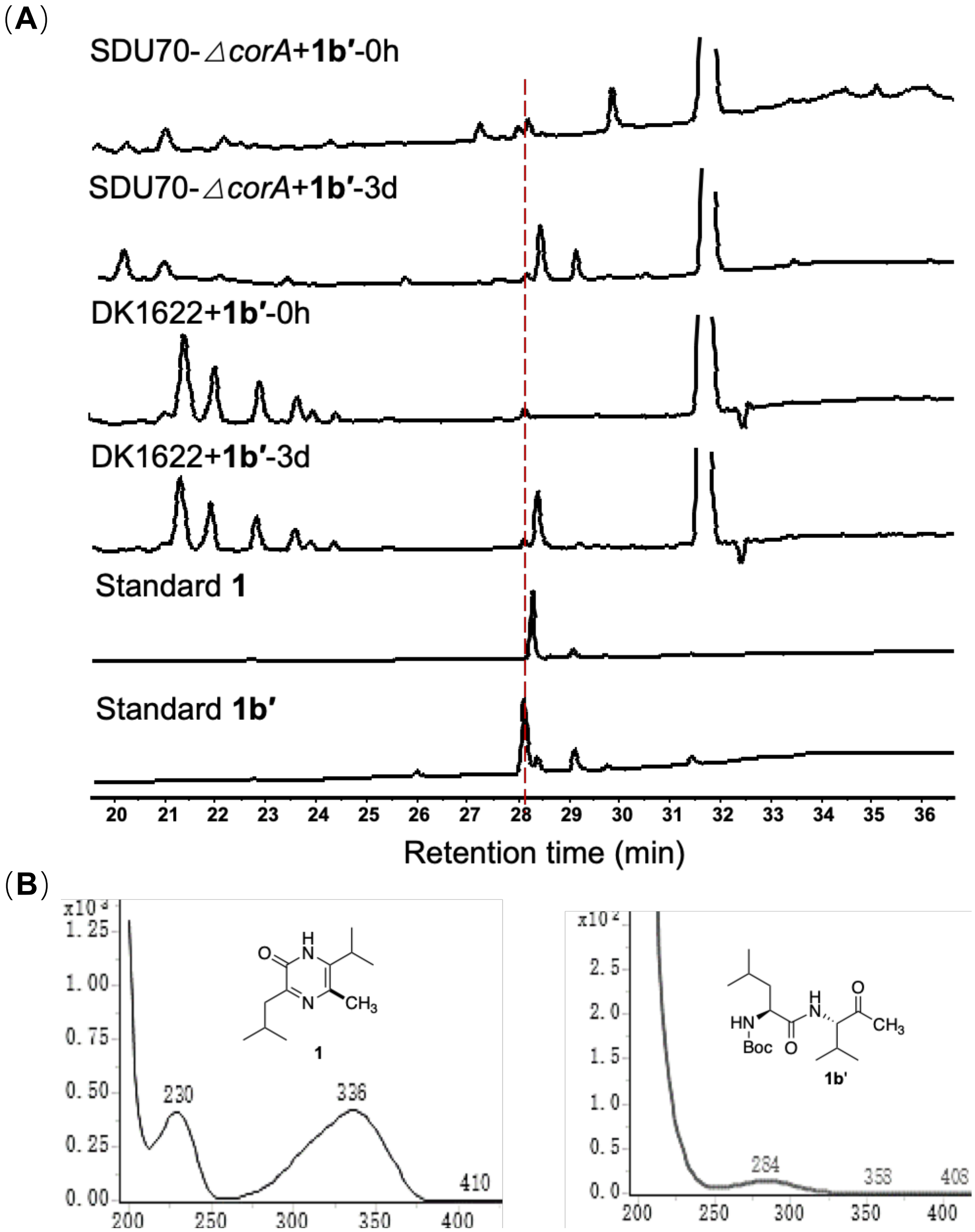

Figure S12. *corB* is a self-resistance gene against the cognate products encoded by *corA*. The displayed is schematic summary of agglutination of SDU70 and DK1622 mutants endowed with different combination of *corA* and *corB*. Compounds **1** or **1b'** were chemically complemented at a concentration of 5 mg/L when applicable. Source data are provided in Figure S13 and Figure S14.

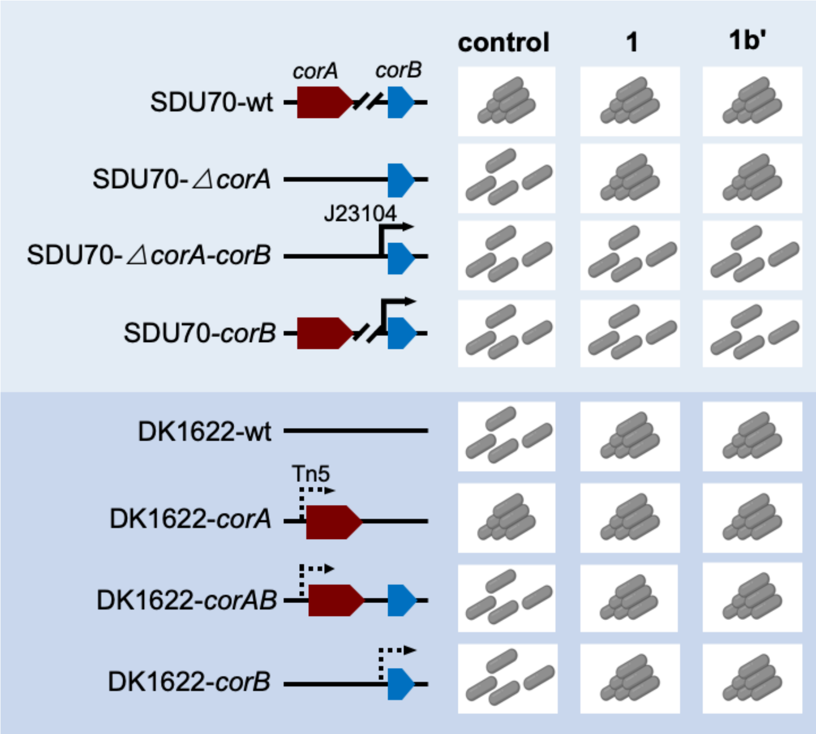

Figure S13. Agglutination assay of SDU70 and DK1622 mutants endowed with different genotypic combination of *corA* and *corB*. The overexpression of *corB* in SDU70 and/or DK1622 could substantially attenuate agglutination that was stimulated by endogenous expression of *corA* and/or exogenous addition of **1** or **1b'**. Compounds **1** and **1b'** were added at 5 mg/L.

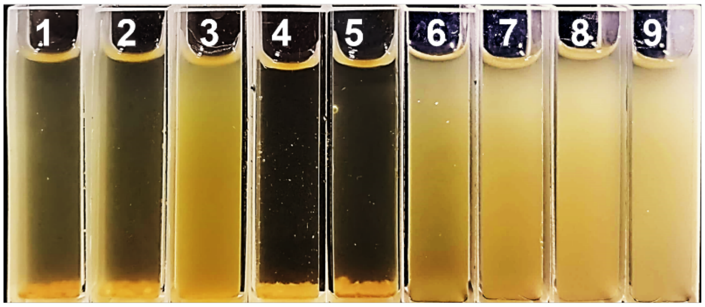

1: SDU70-wt
2: SDU70-*corA*
3: SDU70-△*corA*
4: SDU70-△*corA* +**1**
5: SDU70-△*corA* +**1b'**
6: SDU70-*corB*
7: SDU70-△*corA-corB*
8: SDU70-△*corA-corB* +**1**
9: SDU70-△*corA-corB*+**1b'**

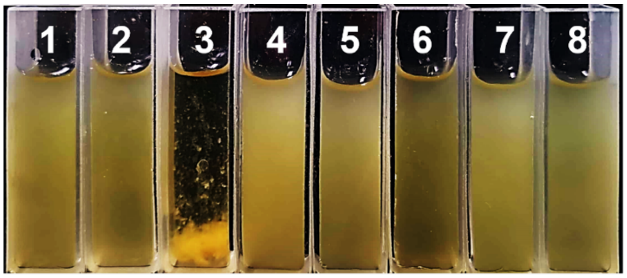

1: DK1622-wt
2: DK1622-vector
3: DK1622-*corA*
4: DK1622-*corB*
5: DK1622-*corAB*
6: DK1622-*corB*+**1**
7: DK1622-*corB*+**9**
8: DK1622-*corB*+**1b'**

Figure S14. Agglutination assay of *corB*-overexpression mutants treated with an increasing concentration of 1 or 1b'. SDU70-*corB* and SDU70-△*corA*-*corB* did not agglutinate even when as high as 50 mg/L of **1** or **1b'** were supplied. The clumping phenomenon of DK1622-*corB* became obvious when 15~20 mg/L of **1** was present. The constitutive promotors J23104 and Tn5 were used for overexpression of *corB* in SDU70 and DK1622, respectively. The promotor J23104 is around five times stronger than the promotor Tn5. [8]

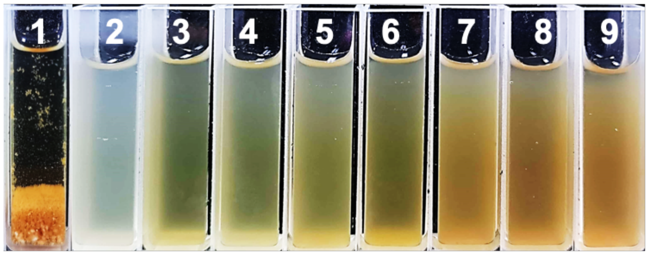

1 : SDU70-*corA*
2 : SDU70-*corB*
3 : SDU70-*corB* +1 mg/L **1**
4 : SDU70-*corB* +2 mg/L **1**
5 : SDU70-*corB* +5 mg/L **1**
6 : SDU70-*corB* +10 mg/L **1**
7 : SDU70-*corB* +15 mg/L **1**
8 : SDU70-*corB* +20 mg/L **1**
9 : SDU70-*corB* +50 mg/L **1**

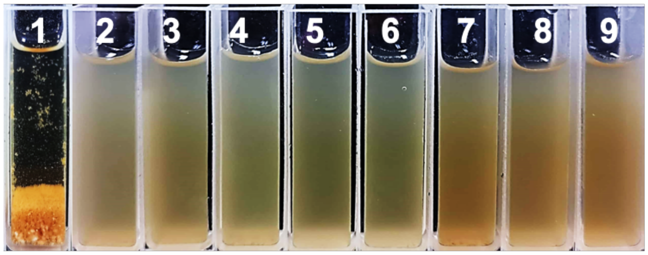

1 : SDU70-*corA*
2 : SDU70-*corB*
3 : SDU70-*corB* +1 mg/L **1b'**
4 : SDU70-*corB* +2 mg/L **1b'**
5 : SDU70-*corB* +5 mg/L **1b'**
6 : SDU70-*corB* +10 mg/L **1b'**
7 : SDU70-*corB* +15 mg/L **1b'**
8 : SDU70-*corB* +20 mg/L **1b'**
9 : SDU70-*corB* +50 mg/L **1b'**

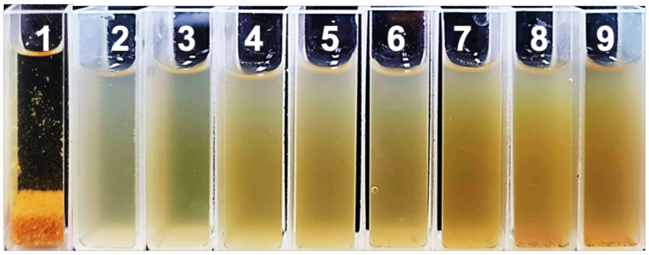

1 : SDU70-wt
2 : SDU70-△*corA*-*corB*
3 : SDU70-△*corA*-*corB* +1 mg/L **1**
4 : SDU70-△*corA*-*corB* +2 mg/L **1**
5 : SDU70-△*corA*-*corB* +5 mg/L **1**
6 : SDU70-△*corA*-*corB* +10 mg/L **1**
7 : SDU70-△*corA*-*corB* +15 mg/L **1**
8 : SDU70-△*corA*-*corB* +20 mg/L **1**
9 : SDU70-△*corA*-*corB* +50 mg/L **1**

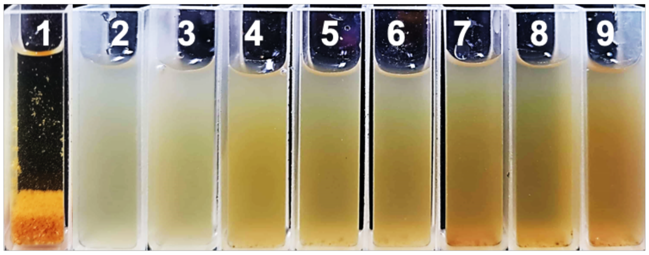

1 : SDU70-wt
2 : SDU70-△*corA*-*corB*
3 : SDU70-△*corA*-*corB* +1 mg/L **1b'**
4 : SDU70-△*corA*-*corB* +2 mg/L **1b'**
5 : SDU70-△*corA*-*corB* +5 mg/L **1b'**
6 : SDU70-△*corA*-*corB* +10 mg/L **1b'**
7 : SDU70-△*corA*-*corB* +15 mg/L **1b'**
8 : SDU70-△*corA*-*corB* +20 mg/L **1b'**
9 : SDU70-△*corA*-*corB* +50 mg/L **1b'**

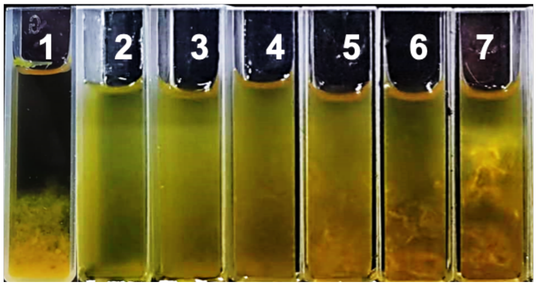

1 : DK1622-*corA*
2 : DK1622-*corB*
3 : DK1622-*corB*+5 mg/L **1**
4 : DK1622-*corB*+10 mg/L **1**
5 : DK1622-*corB*+15 mg/L **1**
6 : DK1622-*corB*+20 mg/L **1**
7 : DK1622-*corB*+50 mg/L **1**

Figure S15. Transcription of *corA* and *corB* in SDU70-wt. (**A**) Gel electrophoresis of PCR products amplified from *corA* and *corB*, respectively. Lanes S, RT-PCR amplification using the cDNA from SDU70-wt as template; Lanes +, positive controls using the genomic DNA of SDU70-wt as the template; Lanes −, negative controls wherein no reverse transcriptase was added; M, the marker. (**B**) Relative quantification of transcription of *corA* and *corB* in SDU70-wt.

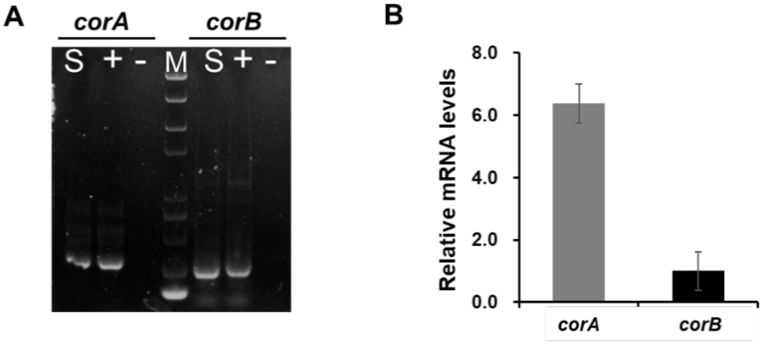

Figure S16. Enzymatic hydrolysis of coralinones using CorB. CorB (Figure S4) was used to hydrolyze compounds **1**, **2**, and **1b'**. Compounds dissolved in the buffer without addition of CorB was used as a control. HPLC-DAD analysis demonstrated that no enzymatic reaction happened.

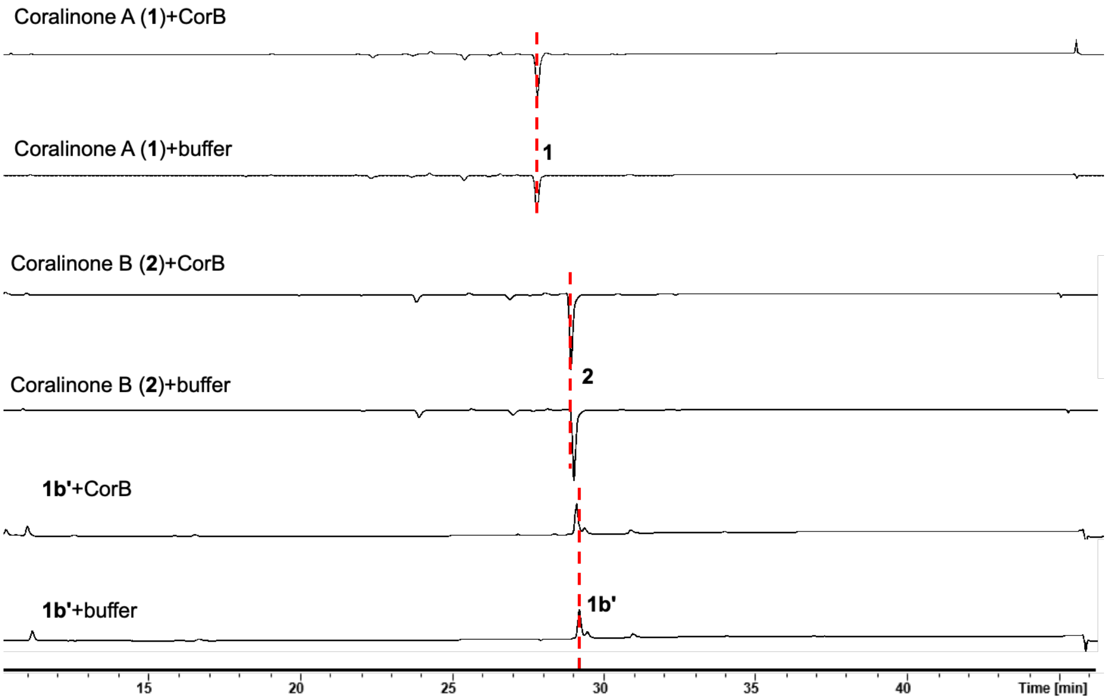

Figure S17. MST assay of CorB to coralinone A. CorB demonstrated excellent binding ability with the dye. The addition of **1** did not disturb CorB-dye binding, as monitored by the instrument.

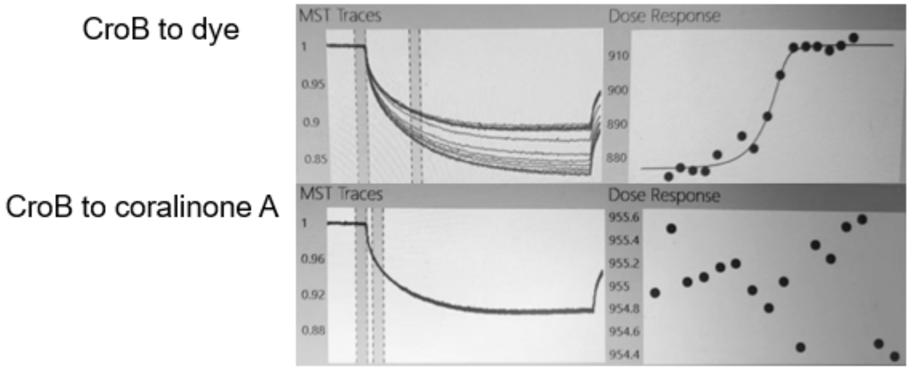

Figure S18. Sequence alignment of CorB with the peptidases DegP and DegQ. The accession number for DegP and DegQ are WP_106490465.1 and WP_127516748.1, respectively. The key residues requisite for the proteolysis activity is highlighted with black triangles.

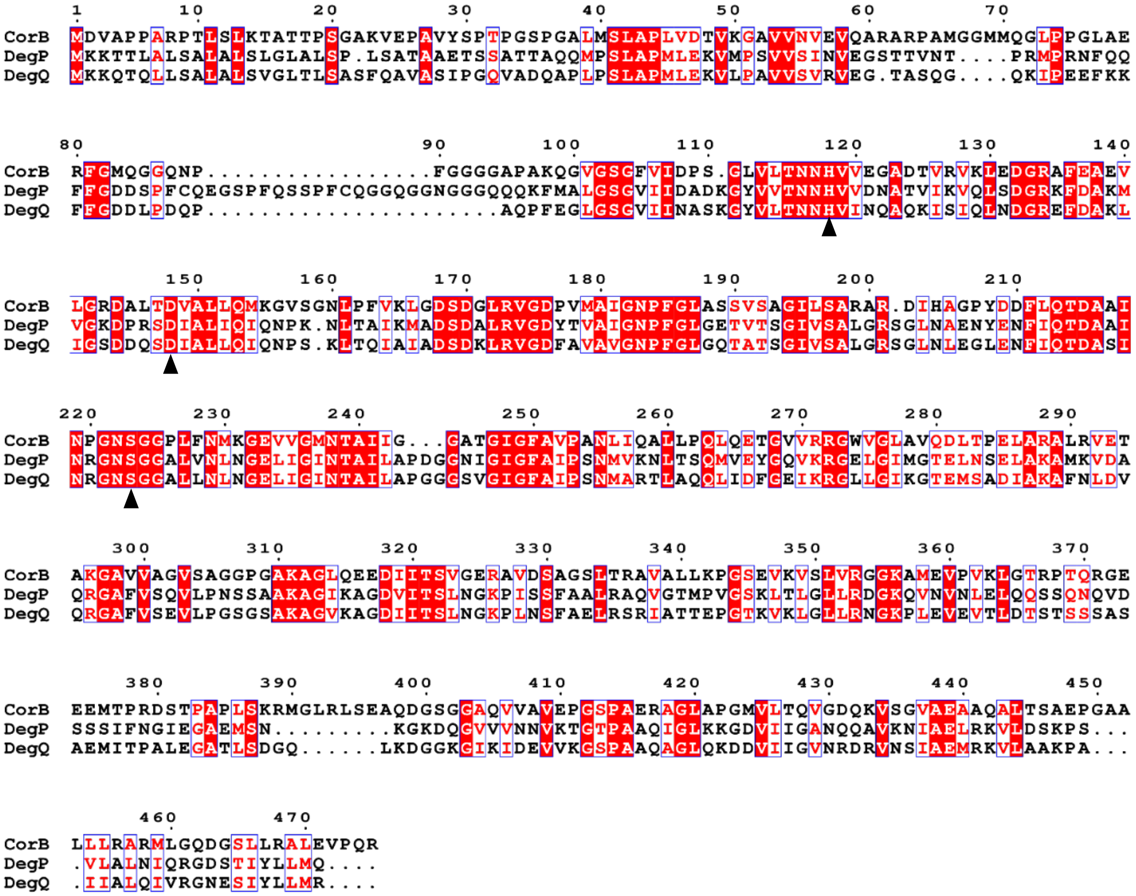

Figure S19. Comparison of 3D structures of CorB, DegP and DegQ. The structure of CorB was predicted by AlphaFold2. The structures of DegP (6JJL) and DegQ (3STI) were retrieved from PDB database. The catalytic triad of Ser-Asp-His in these three proteins was zoomed and highlighted.

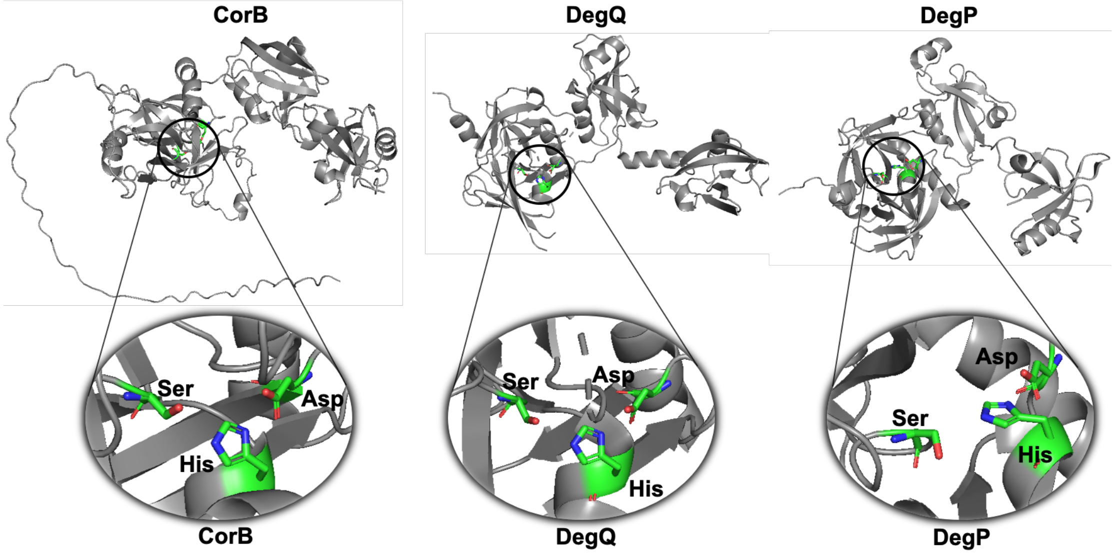

Figure S20. Taxonomic identification of the myxobacterium SDU70. The phylogenetic tree was constructed based on 16S rRNA gene sequences of myxobacteria. The position of *Corallococcus exiguus* SDU70 among related taxa shows it falls into the genus *Corallococcus*. The neighbor-joining method was used, and only bootstrap values above 70% (percentages of 1000 replications) are indicated. Bar, 0.005 nucleotide substitutions per site.

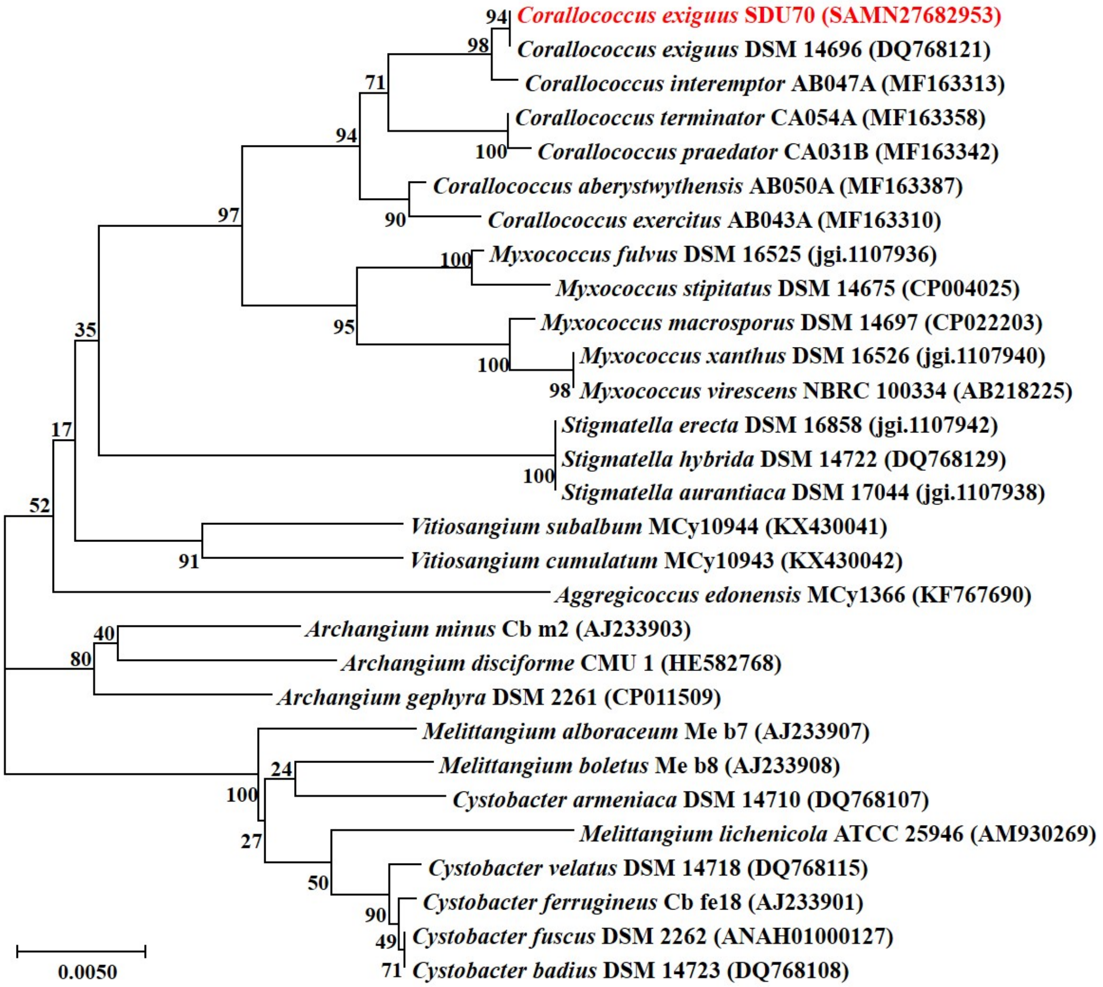

Figure S21. Diagrammatic sketch for the disruption and/or overexpression of *corA* and *corB* in SDU70. All the genetic manipulations in SDU70 were achieved by single-crossover recombination. The primer pair △*corA*-*corB*-check-F/R was used to verify the insertional disruption of *corA* and meanwhile overexpression of *corB*. "-" in the gel electrophoresis represent the negative controls of PCR amplification using the genome of the SDU70 wild type as a template. The primers for PCR verification of correct mutants are listed in Table S6. Furthermore, the correct genotypes could also been validated by the metabolic analysis (Figure S8).

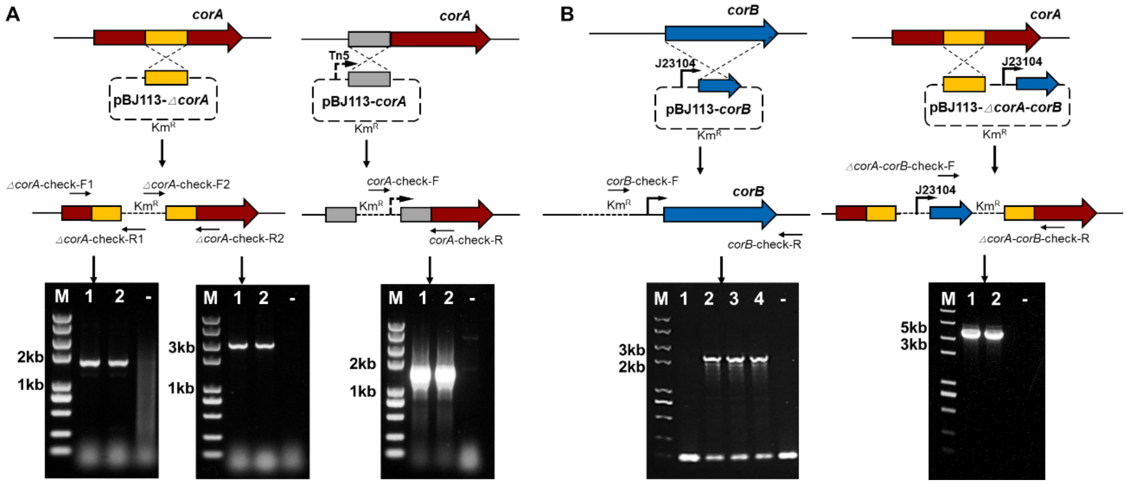

Figure S22. Schematic diagram for the plasmid construction for heterologous expression of *corA* and/or *corB* in DK1622. The plasmid pSWU19 can be integrated into the genome of DK1622 by the action of integrase. RBS: ribosome binding site.

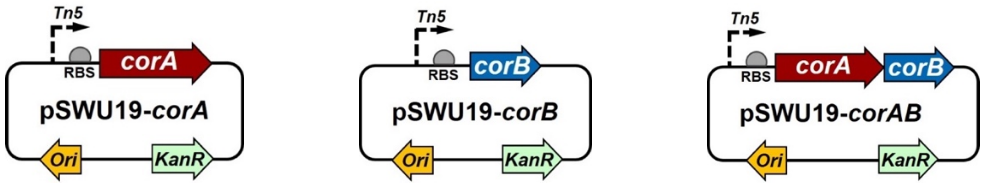

All spectra employed in structure elucidation.

S1. ^{1}H NMR (600 MHz, methanol-*d*4) of coralinone A (1).

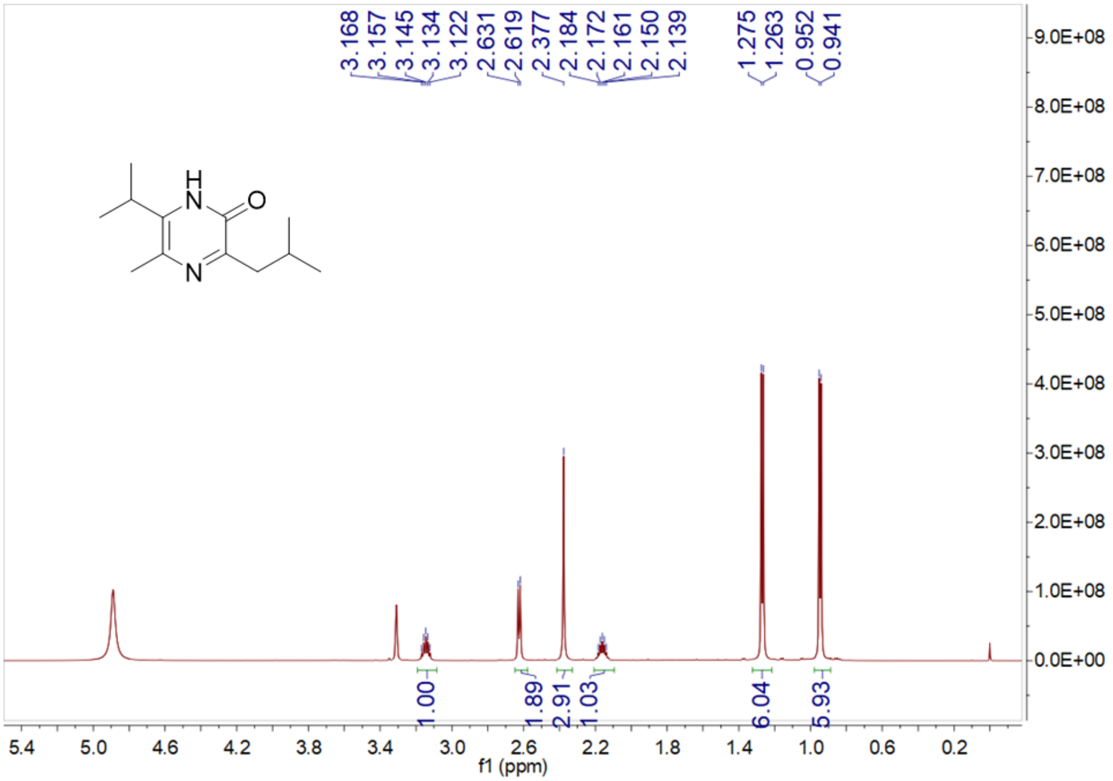

S2. ^{13}C NMR (150 MHz, methanol-*d*4) spectrum of coralinone A (1).

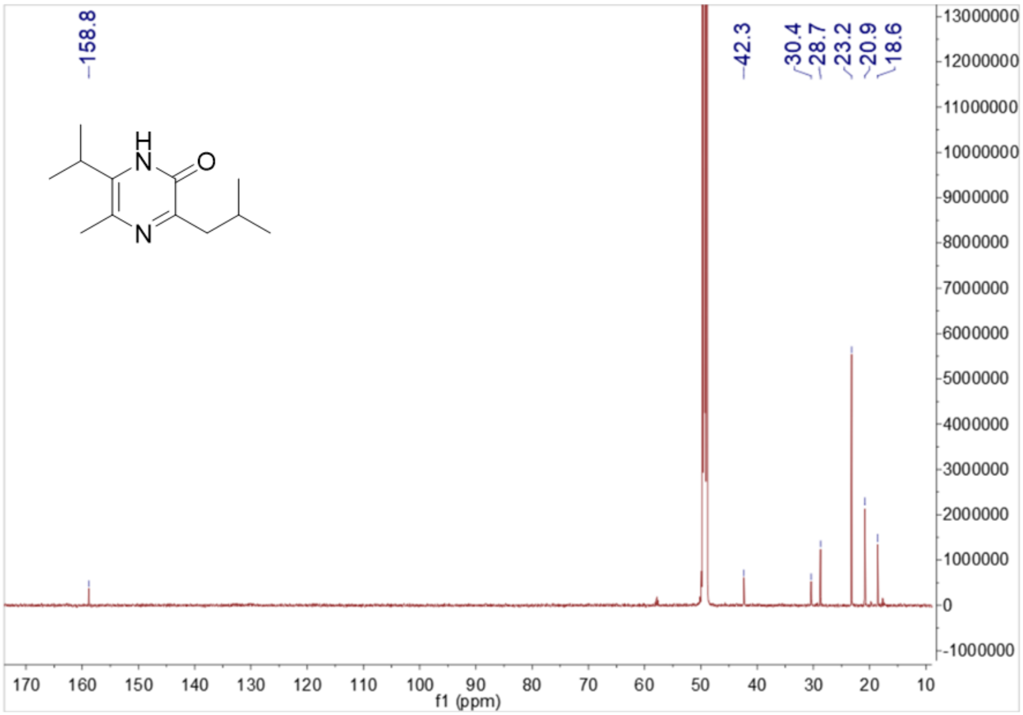

S3. DEPTQ (150 MHz, methanol-d_4) spectrum of coralinone A (1).

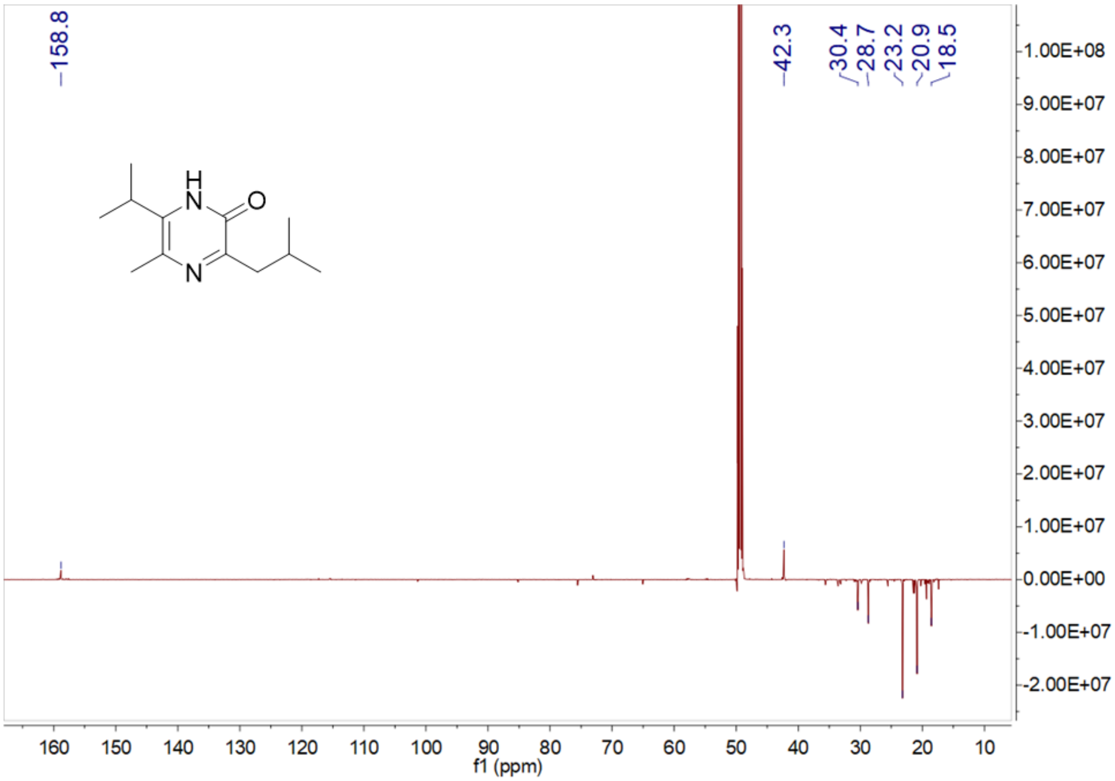

S4. HSQC (600 MHz, methanol-d_4) spectrum of coralinone A (1).

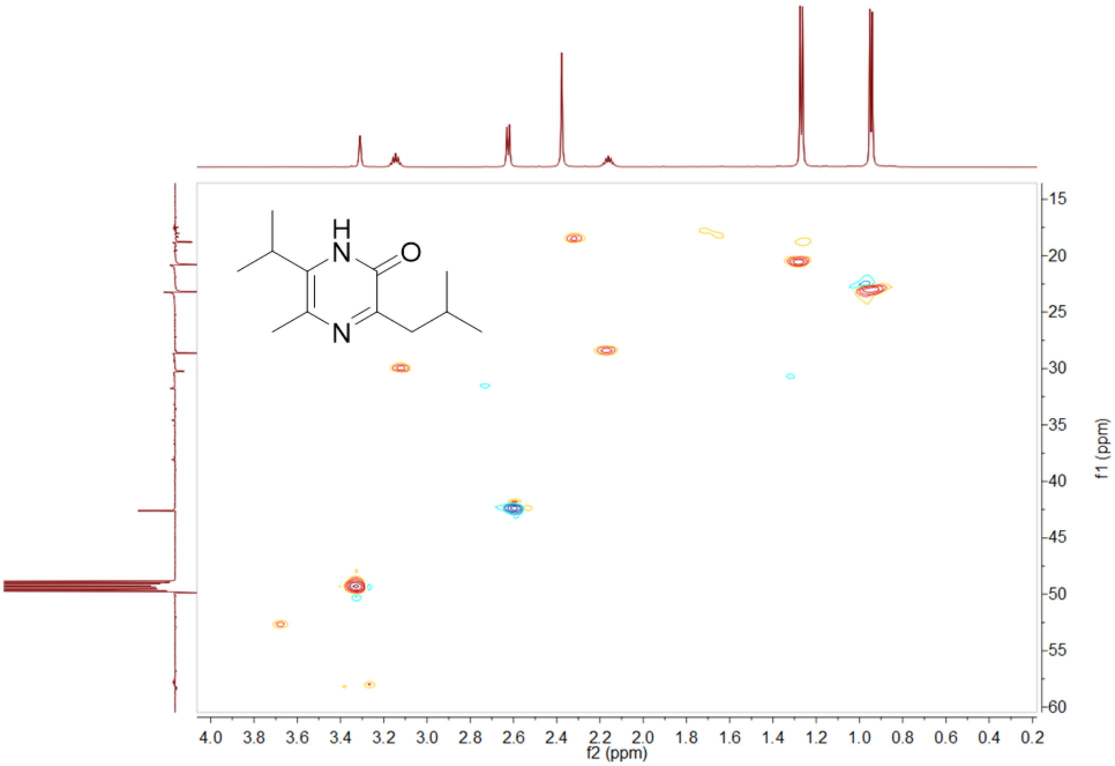

S5. HMBC (600 MHz, methanol-d_4) spectrum of coralinone A (1).

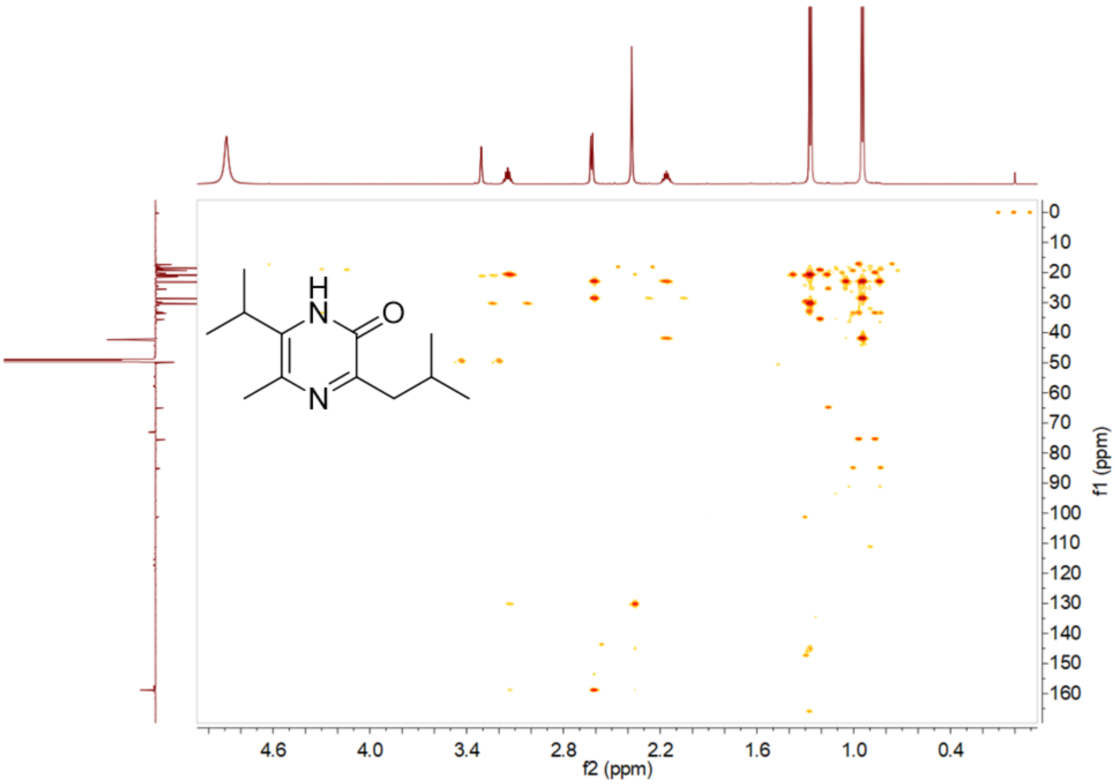

S6. ^{1}H-^{1}H COSY (600 MHz, methanol-d_4) spectrum of coralinone A (1).

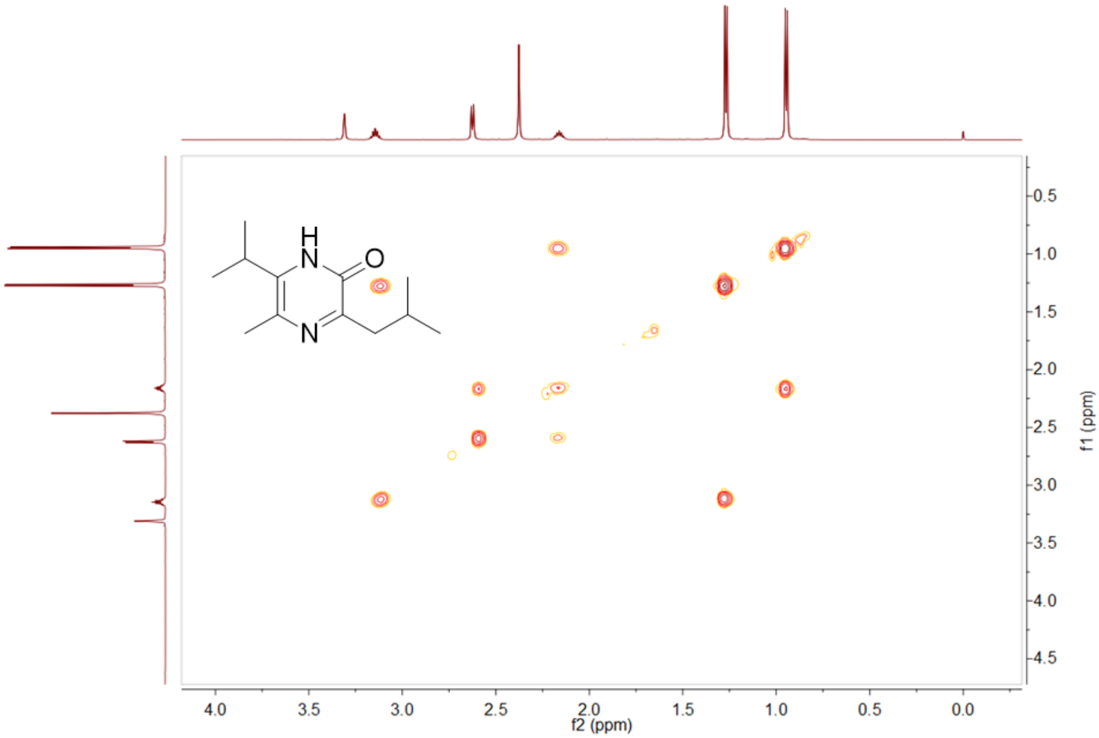

S7. ^{1}H NMR (600 MHz, methanol-d_4) of coralinone B (2).

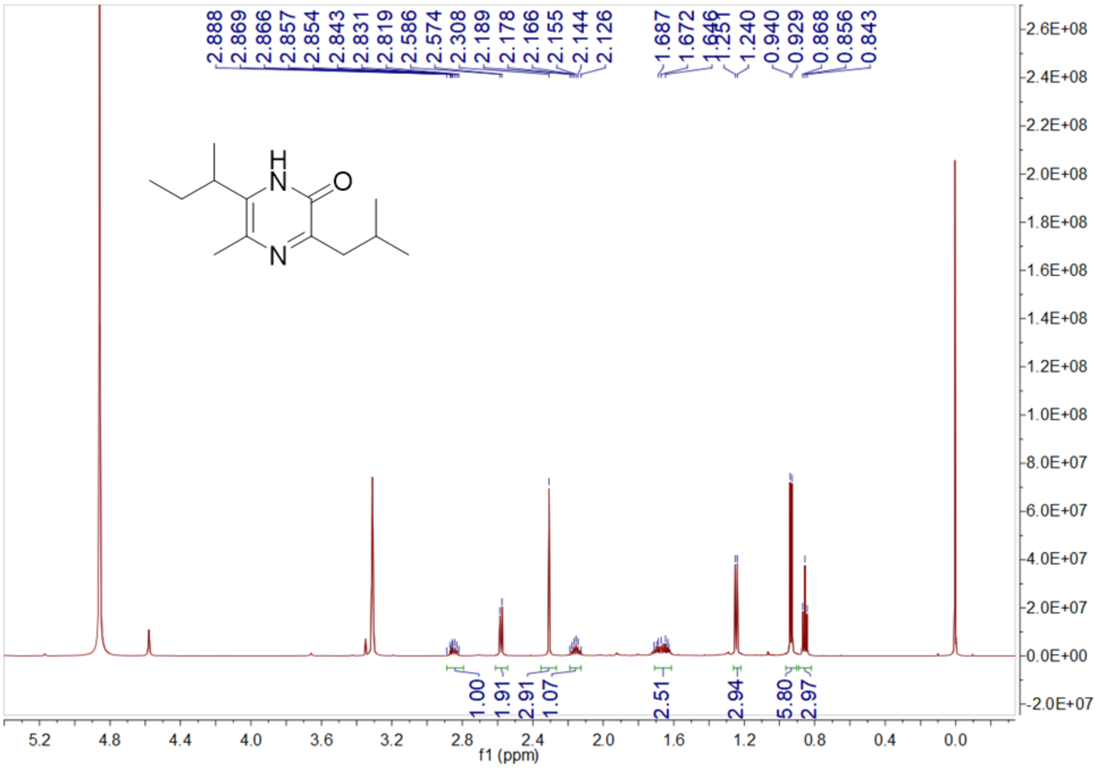

S8. ^{13}C NMR (150 MHz, methanol-d_4) spectrum of coralinone B (2).

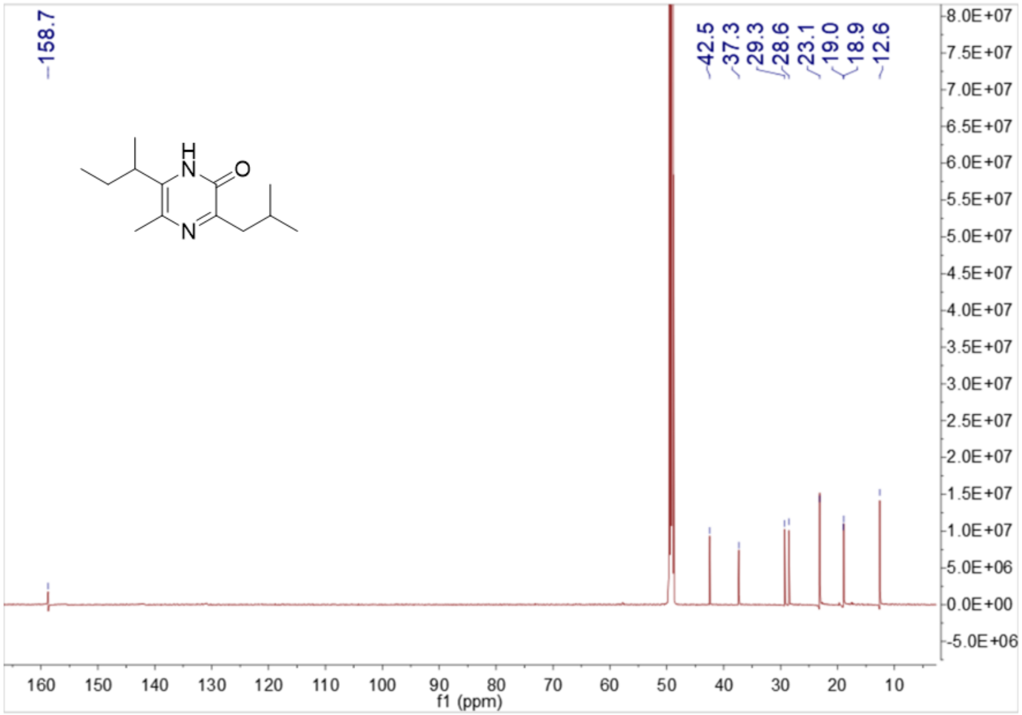

S9. DEPTQ (150 MHz, methanol-d4) spectrum of coralinone B (2).

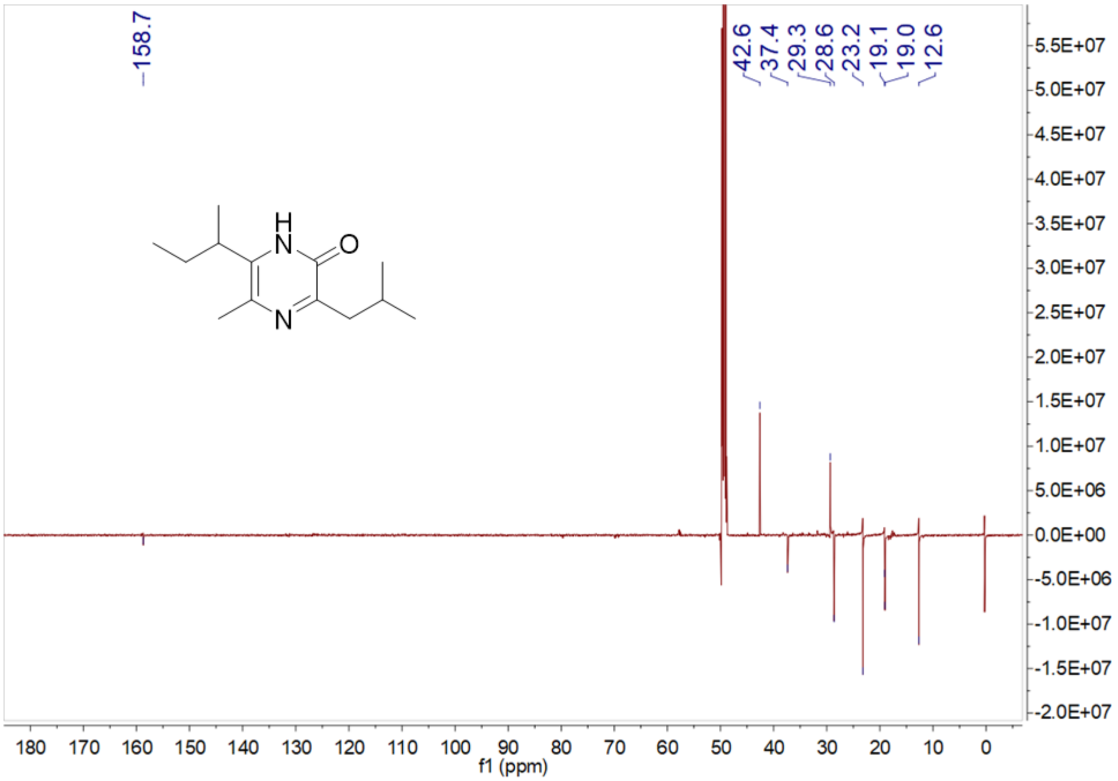

S10. HSQC (600 MHz, methanol-d_4) spectrum of coralinone B (2).

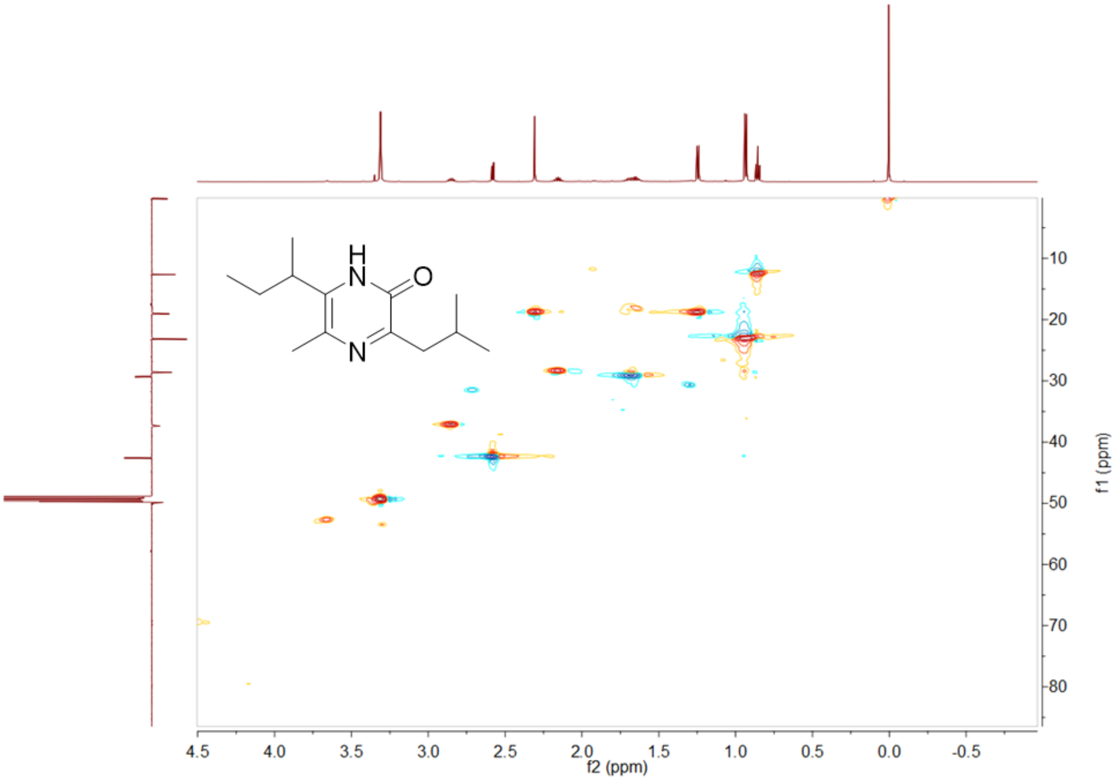

S11. HMBC (600 MHz, methanol-d_4) spectrum of coralinone B (2).

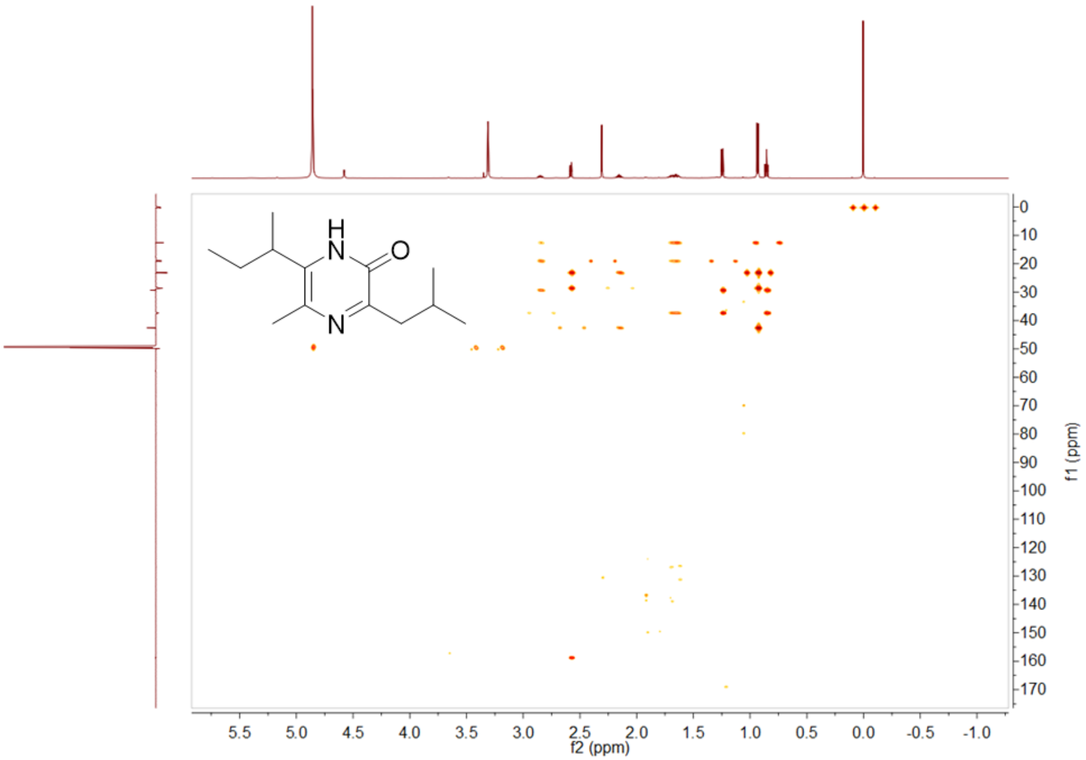

S12. ^{1}H-^{1}H COSY (600 MHz, methanol-d4) spectrum of coralinone B (2).

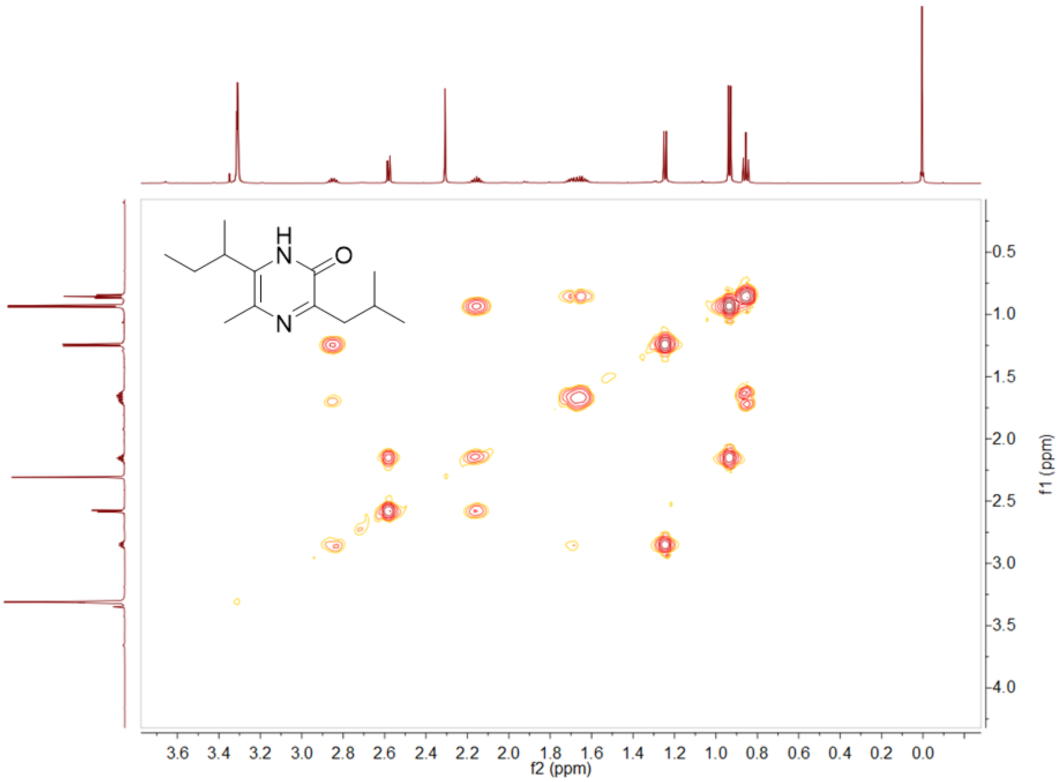

S13. HRESIMS spectrum of coralinone B (2).

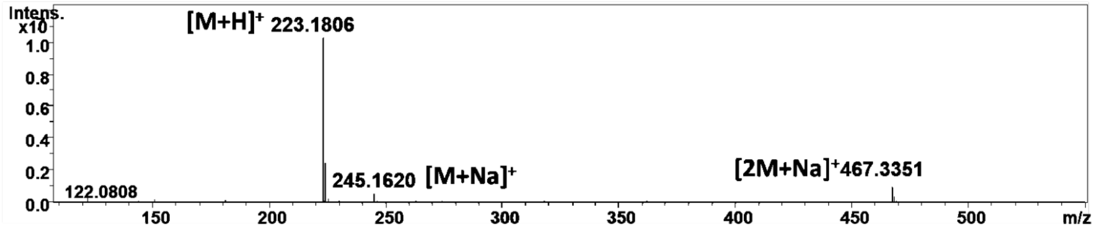

S14. HRESIMS spectrum of coralinone A (1).

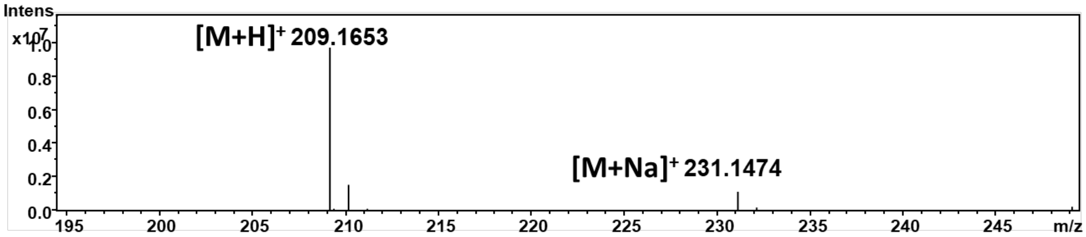

S15. ^{1}H NMR (600 MHz, methanol-d4) of Leu-Val-SNAC.

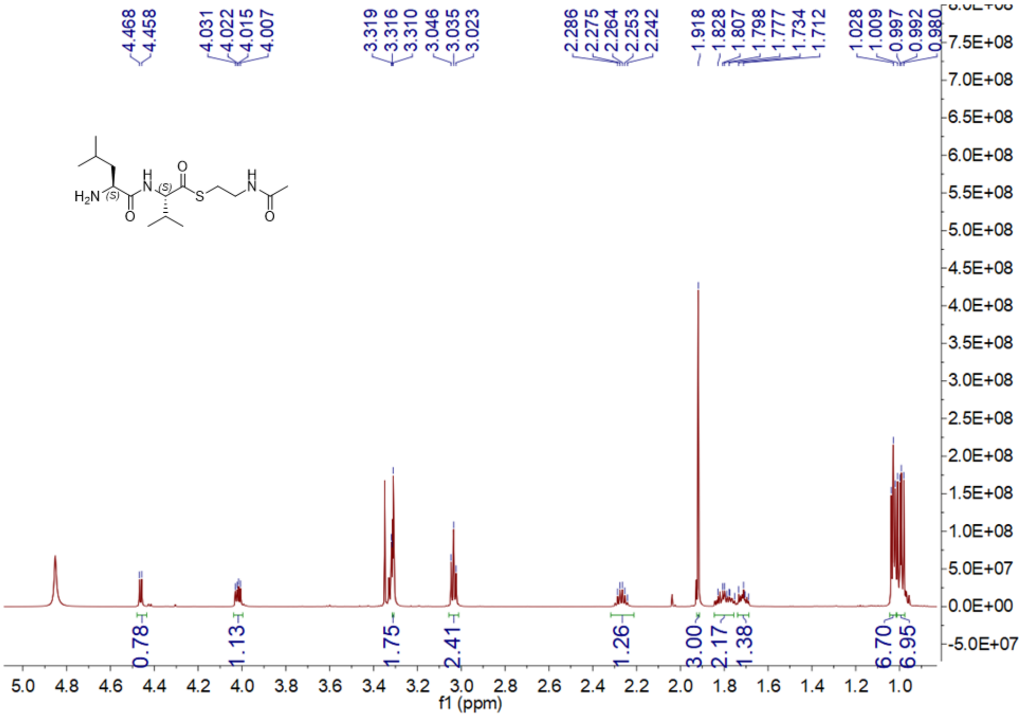

S16.DEPTQ (150 MHz, methanol-d_4) spectrum of Leu-Val-SNAC.

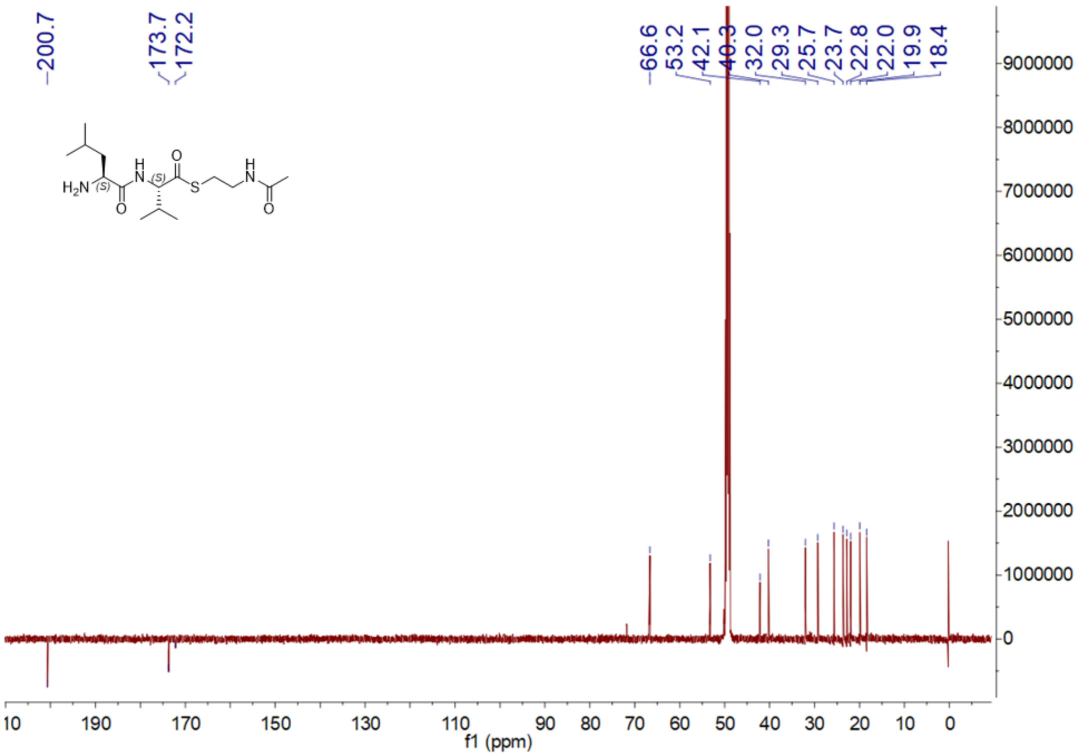

S17. ^{1}H NMR (600 MHz, $CDCl_3$) of 1a''.

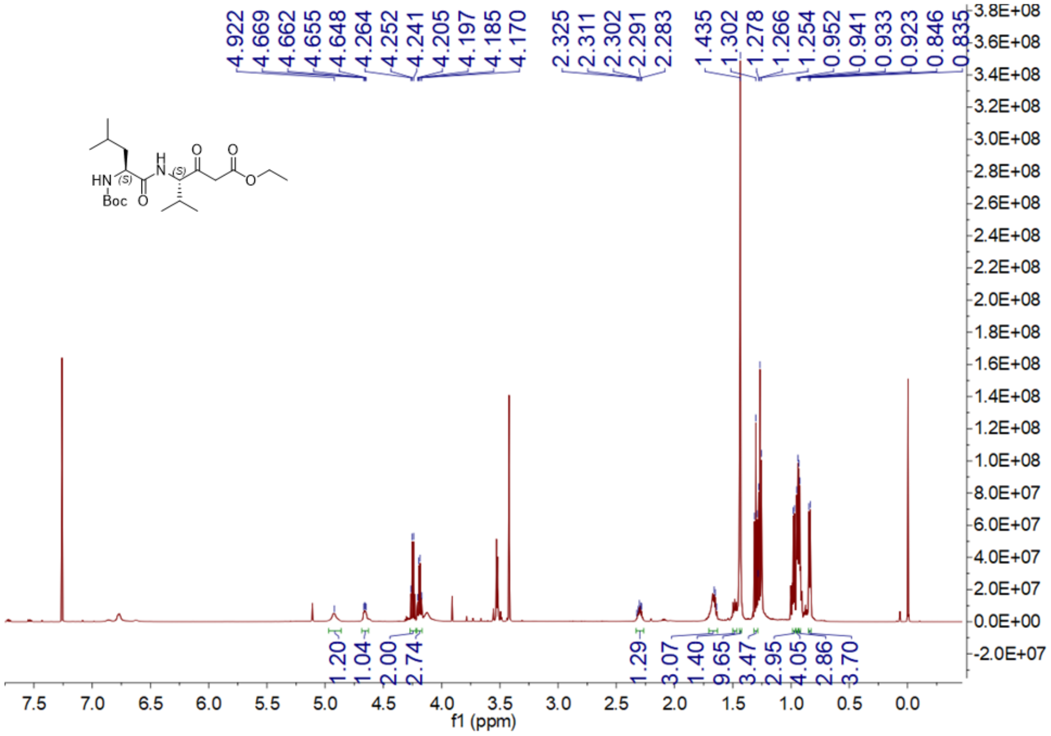

S18. ^{13}C NMR (150 MHz, $CDCl_3$) of 1a".

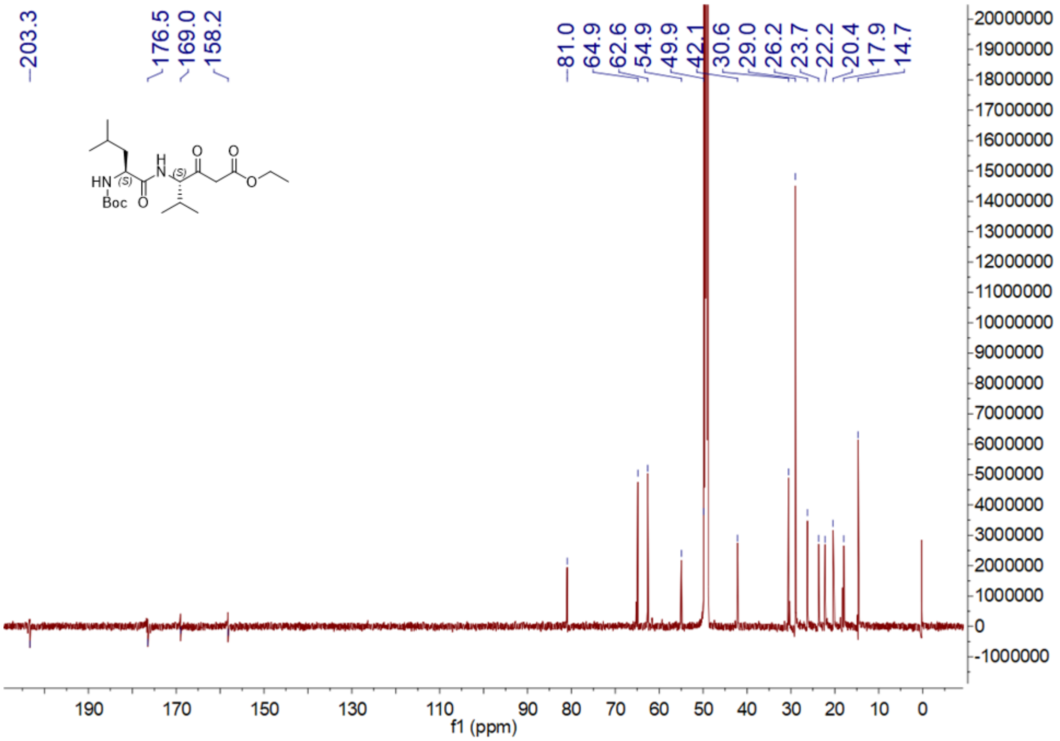

S19. ^{1}H NMR (600 MHz, methanol-d_4) of cyclic-Leu-Val-ethyl formate.

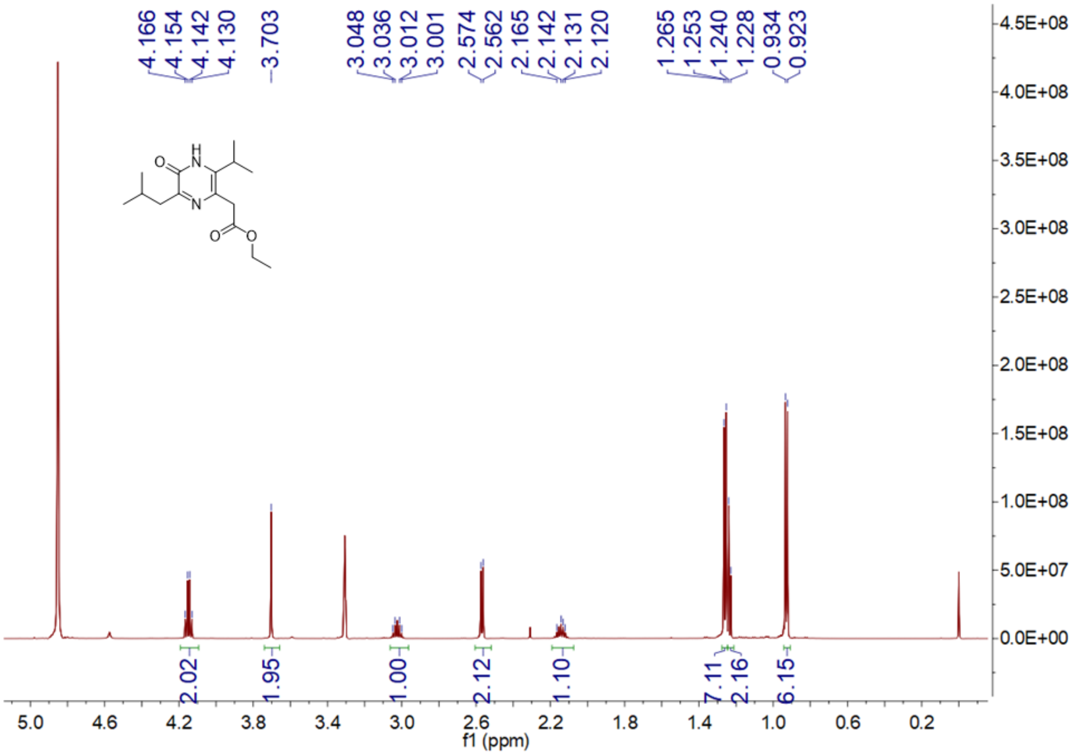

S20.^{13}C NMR (150 MHz, methanol-d_4) spectrum of cyclic-Leu-Val-ethyl formate.

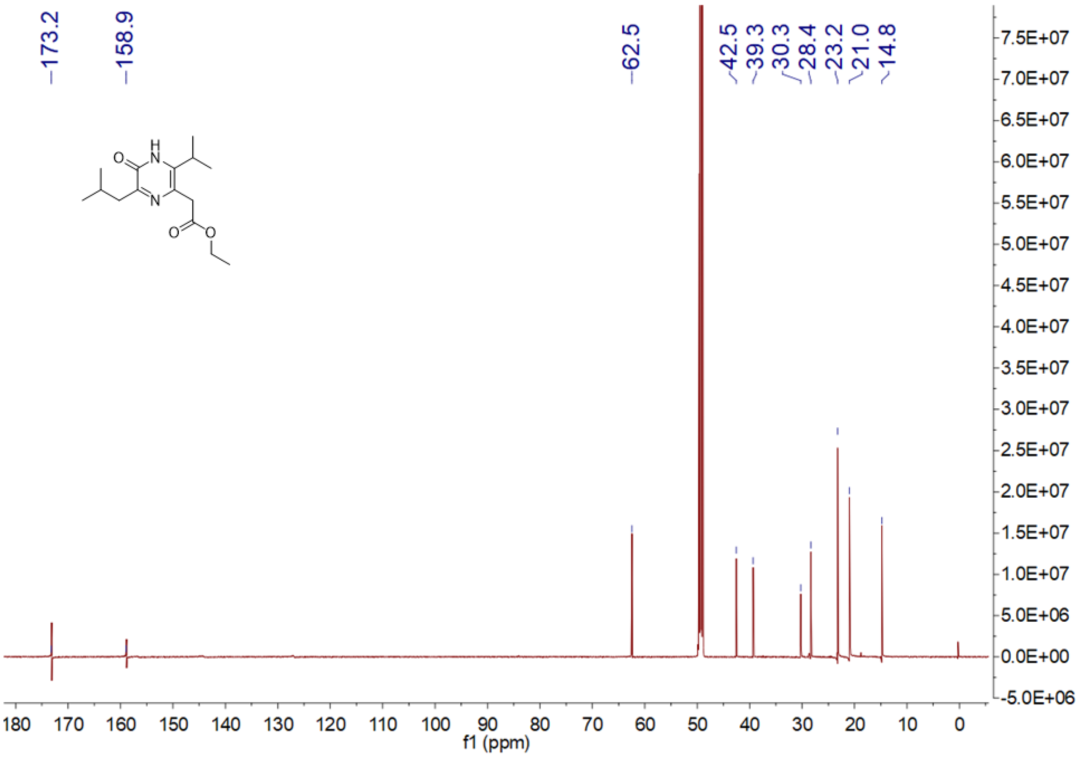

S21. ^{1}H NMR (600 MHz, $CDCl_3$) of 1b'.

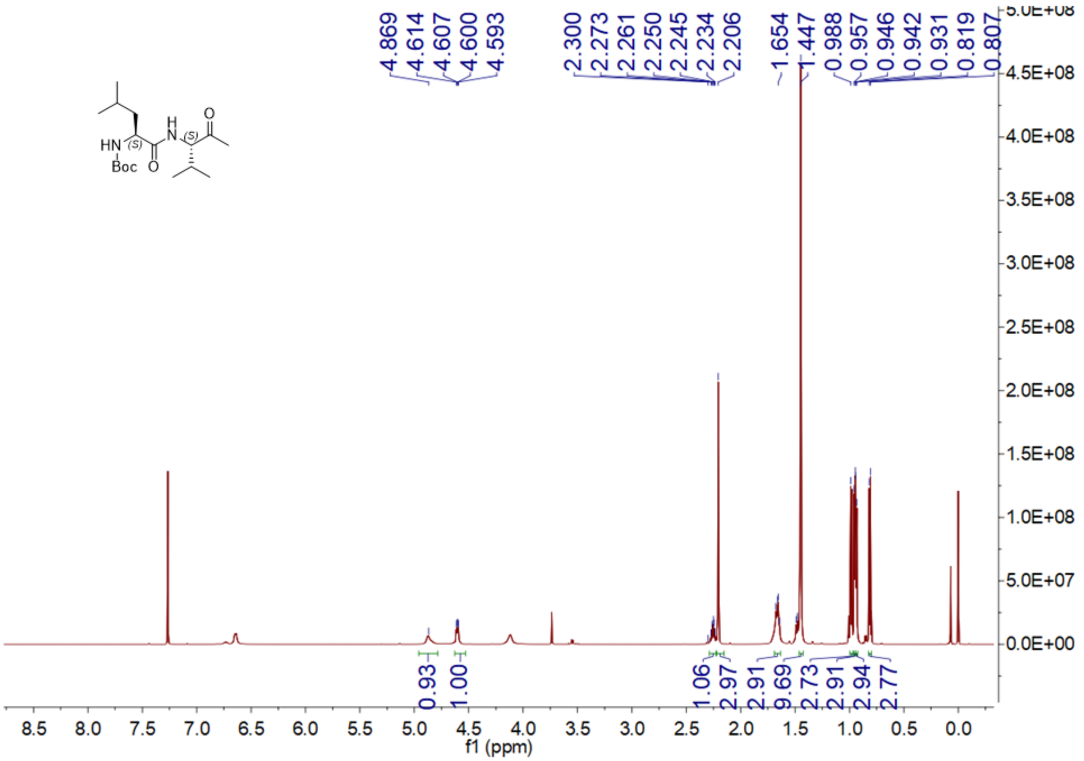

S22.^{13}C NMR (150 MHz, methanol-d_4) spectrum of 1b'.

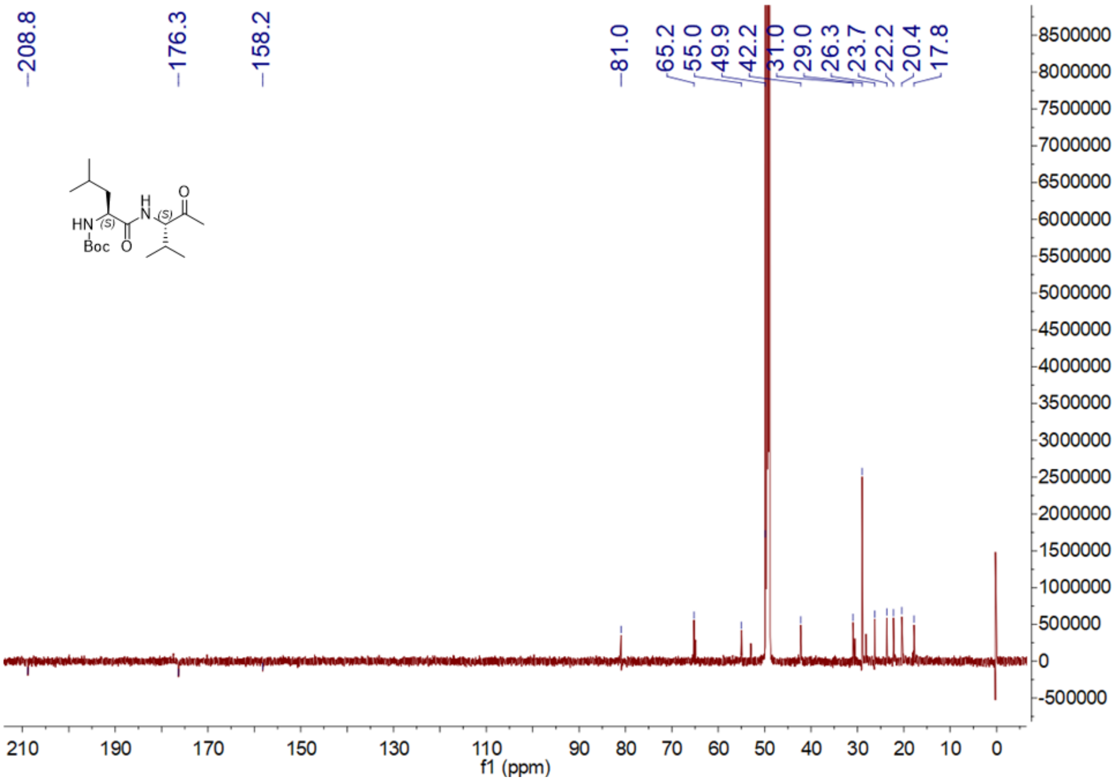

S23. ^{1}H NMR (600 MHz, methanol-d_4) of Boc-Leu-Val-hydroxy-methylhexanoic acid.

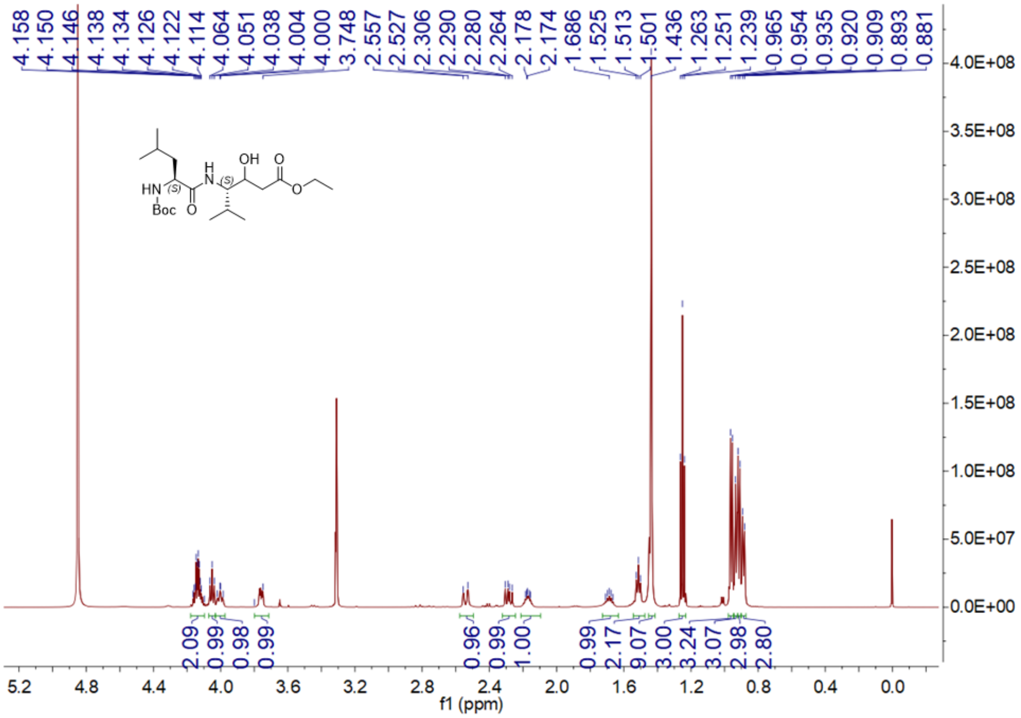

S24.^{13}C NMR (150 MHz, methanol-d_4) spectrum of Boc-Leu-Val-hydroxy-methylhexanoic acid.

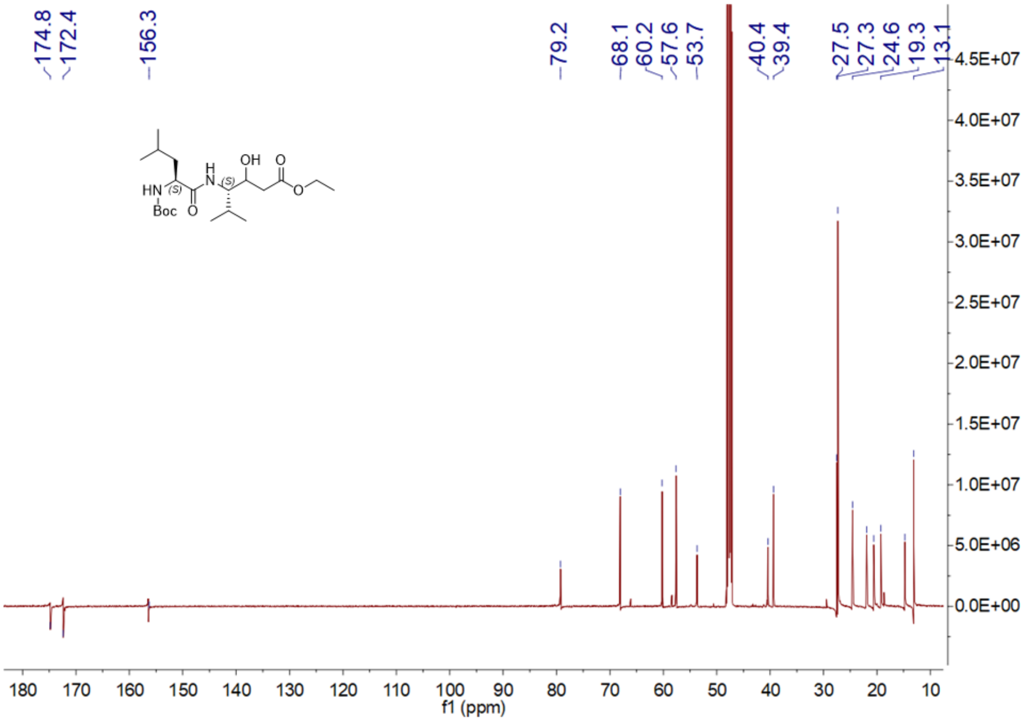

S25. ^{1}H NMR (600 MHz, methanol-d_4) of pre-8.

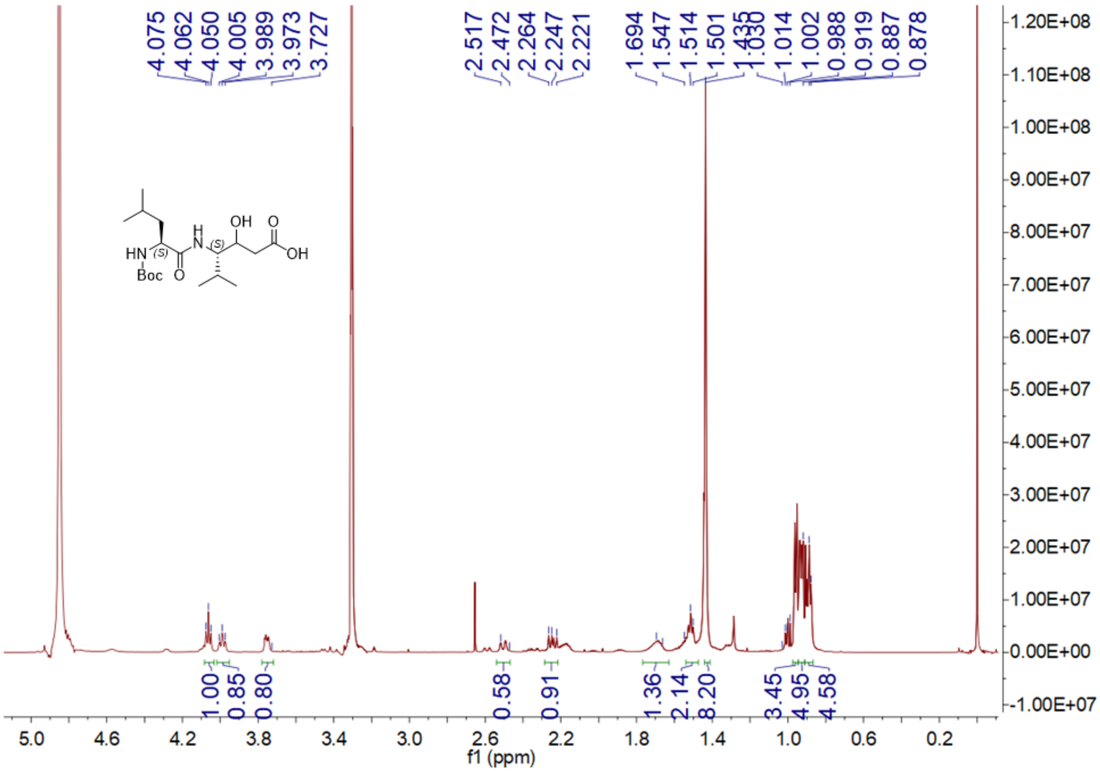

S26.^{13}C NMR (150 MHz, methanol-d_4) spectrum of pre-8.

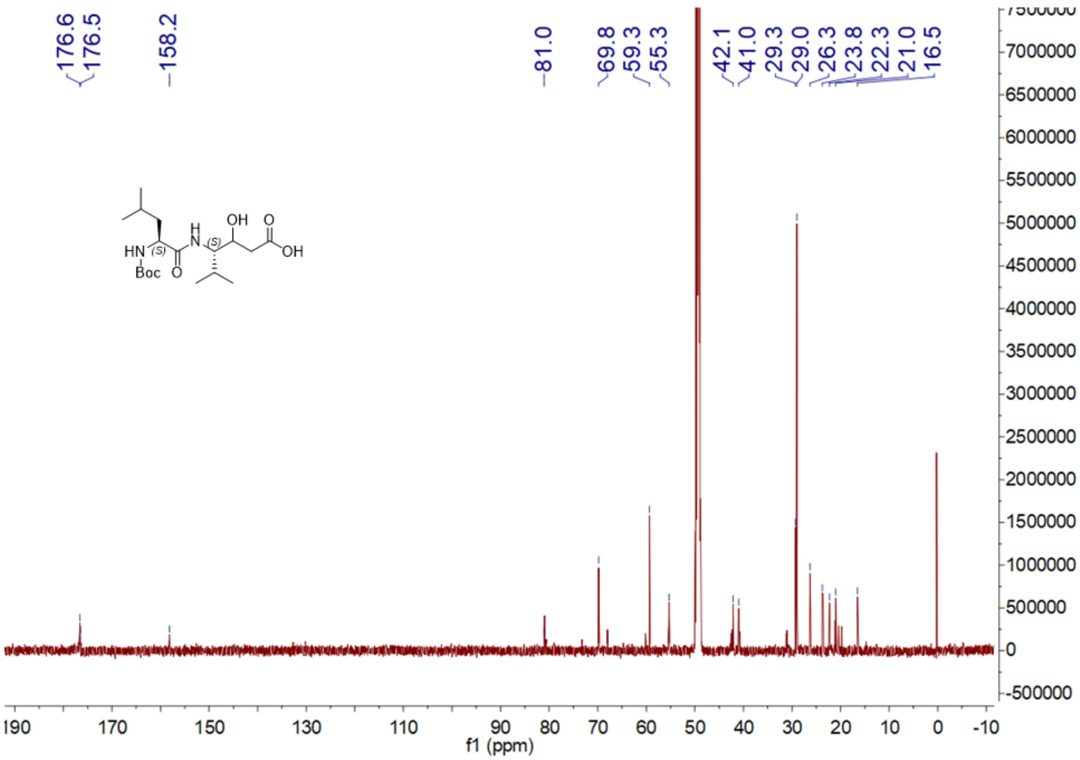

S27. ^{1}H NMR (600 MHz, $CDCl_3$) of 10b'.

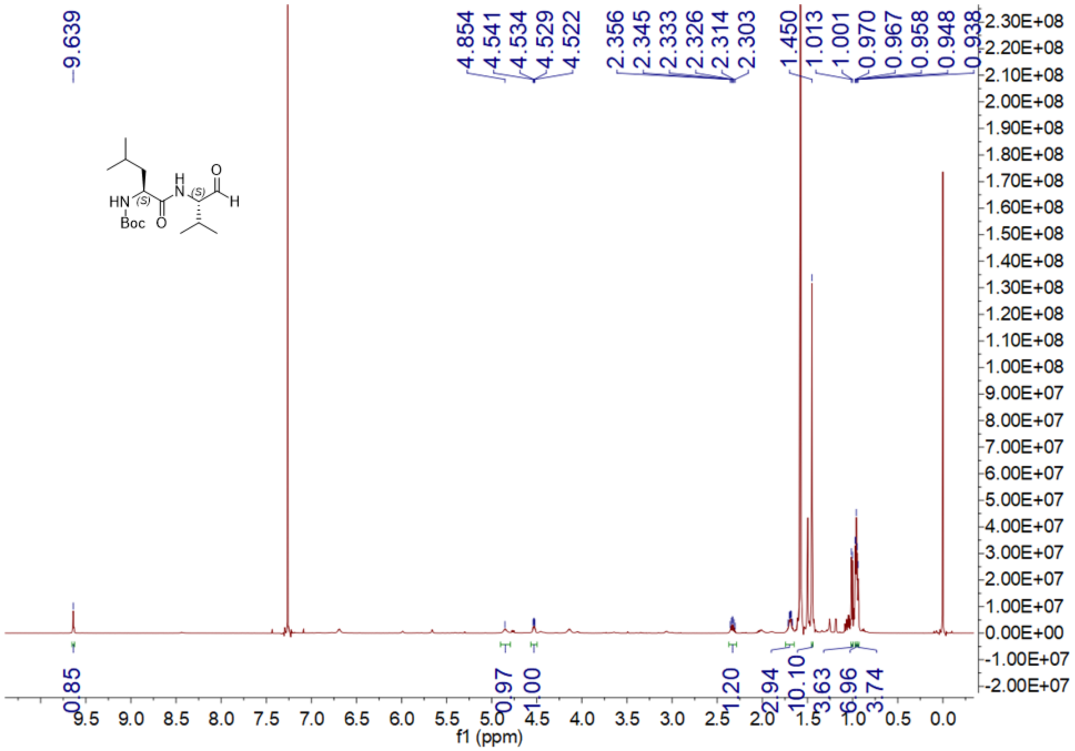

S28. ^{1}**H NMR (600 MHz, methanol-*d*$_4$) of 10.**

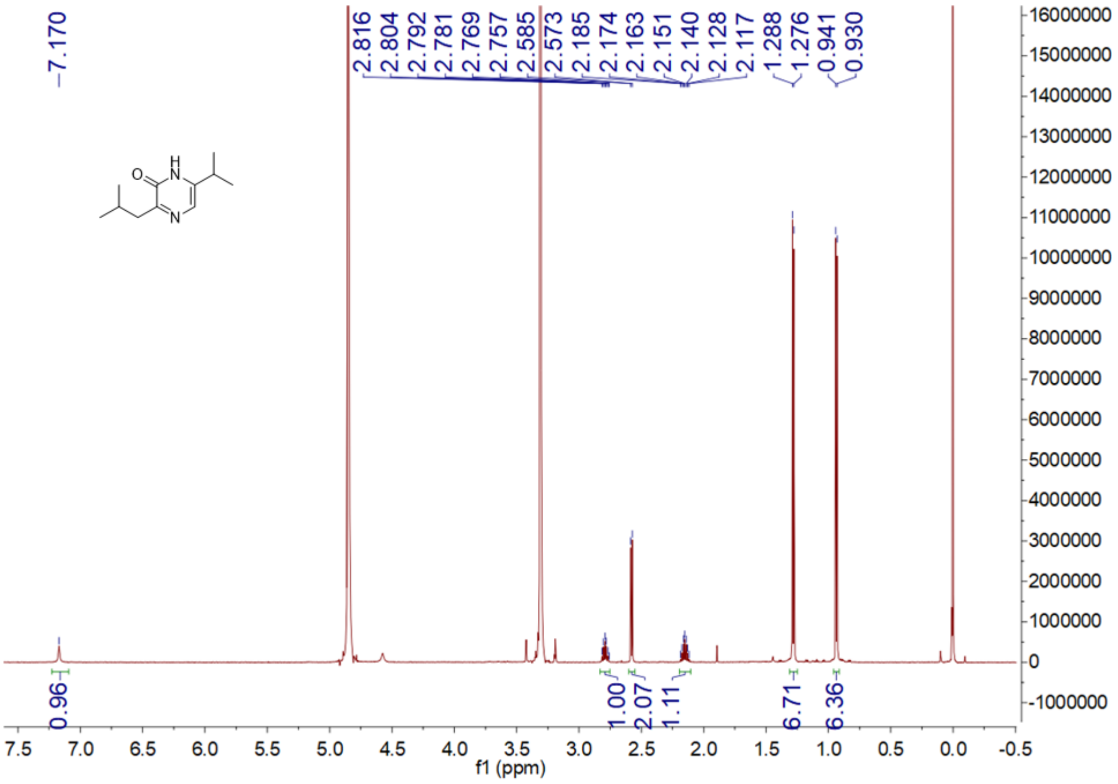

S29. HRESIMS spectrum of synthesis compounds.

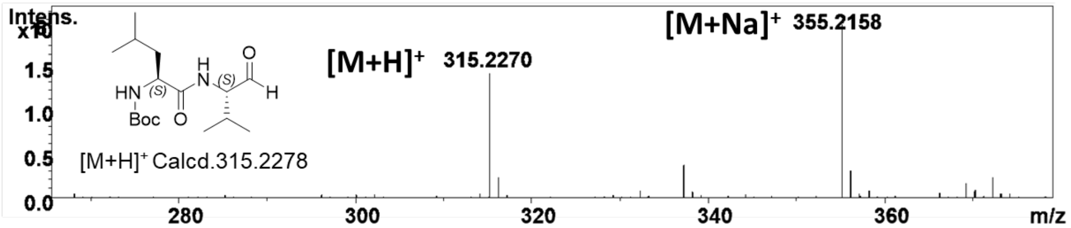

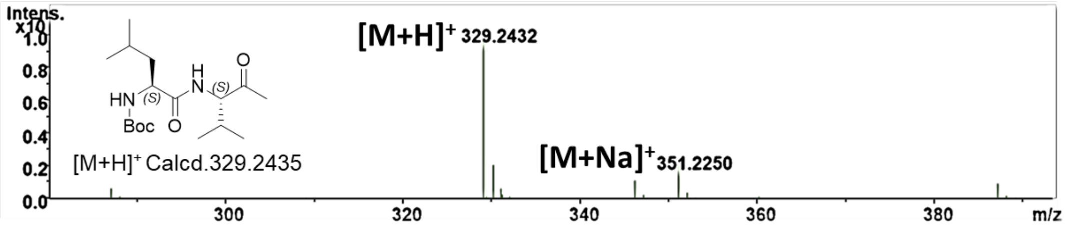

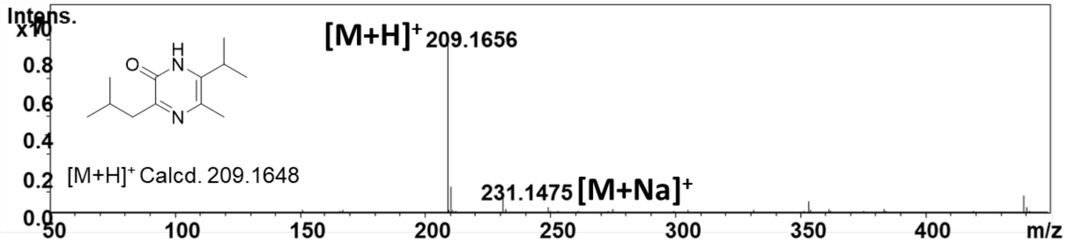
[M+H]+ 209.1656
231.1475 [M+Na]+
[M+H]+ Calcd. 209.1648

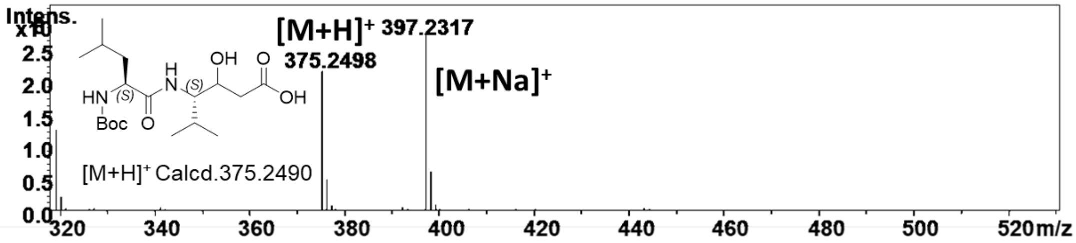
[M+H]+ 375.2498
397.2317 [M+Na]+
[M+H]+ Calcd.375.2490

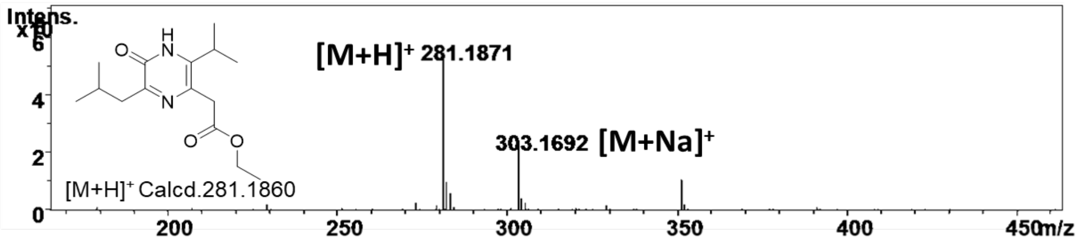
[M+H]+ 281.1871
303.1692 [M+Na]+
[M+H]+ Calcd.281.1860

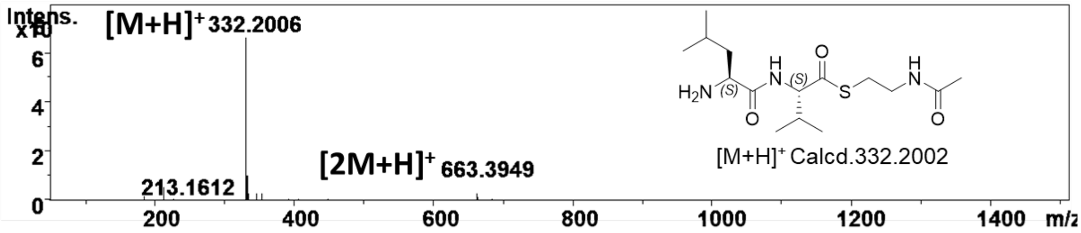
[M+H]+ 332.2006
213.1612
[2M+H]+ 663.3949
[M+H]+ Calcd.332.2002

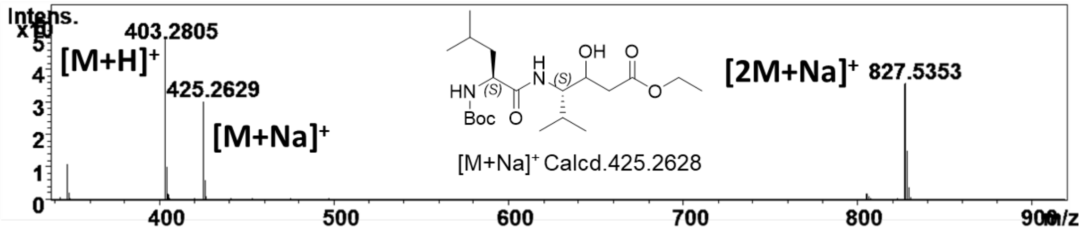
[M+H]+ 403.2805
425.2629 [M+Na]+
[2M+Na]+ 827.5353
[M+Na]+ Calcd.425.2628

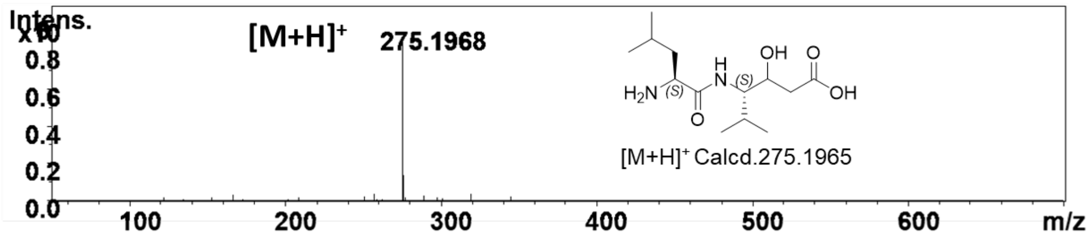
[M+H]+ 275.1968
[M+H]+ Calcd.275.1965

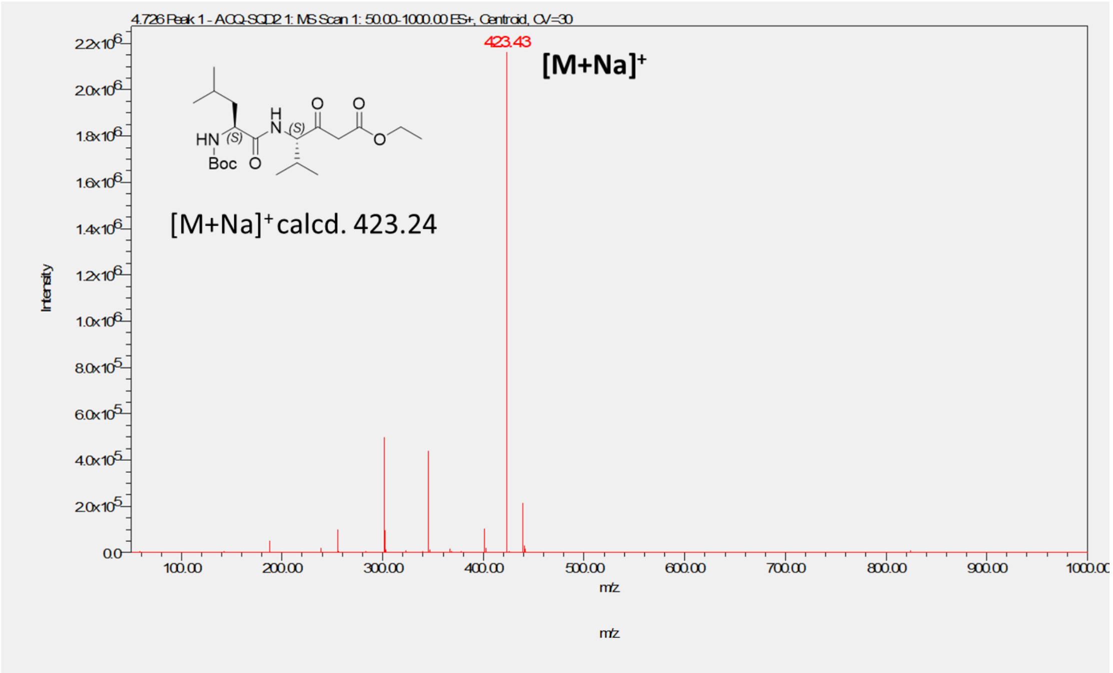
4.726 Peak 1 - ACQ-SQD2 1: MS Scan 1: 50.00-1000.00 ES+, Centroid, CV=30
423.43
[M+Na]+
HN
Boc
(S)
O
H
N
(S)
O
O
O
[M+Na]+ calcd. 423.24
Intensity
m/z

References.

1. Li, Y.; Zhuo, L.; Li, X.; Zhu, Y.; Wu, S.; Shen, T.; Hu, W.; Li, Y. Z.; Wu, C., Myxadazoles, Myxobacterium-Derived Isoxazole-Benzimidazole Hybrids with Cardiovascular Activities. *Angew. Chem. Int. Ed. Engl.* **2021,** *60* (40), 21679-21684.
2. Hu, J. Q.; Wang, J. J.; Li, Y. L.; Zhuo, L.; Zhang, A.; Sui, H. Y.; Li, X. J.; Shen, T.; Yin, Y.; Wu, Z. H.; Hu, W.; Li, Y. Z.; Wu, C., Combining NMR-Based Metabolic Profiling and Genome Mining for the Accelerated Discovery of Archangiumide, an Allenic Macrolide from the Myxobacterium *Archangium violaceum* SDU8. *Org. Lett.* **2021,** *23* (6), 2114-2119.
3. Dale, K., Coupling cell movement to multicellular development in myxobacteria. *Nat. Rev. Microbiol.* **2003,** *1* (1), 45-54.
4. R, Z. D.; E, S. A.; Zhaomin, Y.; R, K. J., Chemosensory pathways, motility and development in Myxococcus xanthus. *Nat. Rev. Microbiol.* **2007,** *5* (11), 862-872.
5. S, W. S.; D, K., Genetic and functional evidence that Type IV pili are required for social gliding motility in Myxococcus xanthus. *Mol. microbiol.* **1995,** *18* (3), 547-558.
6. Ueki, T.; Inouye, S.; Inouye, M., Positive-negative KG cassettes for construction of multi-gene deletions using a single drug marker. *Gene* **1996,** *183* (1), 153-157.
7. Pfeifer, B. A.; Admiraal, S. J.; Gramajo, H.; Cane, D. E.; Khosla, C., Biosynthesis of Complex Polyketides in a Metabolically Engineered Strain of E. coli. *Science* **2001,** *291* (5509), 1790-1792.
8. WeiFeng, H.; Luo, N.; XinJing, Y.; LeLe, Z.; Wei, H.; YueZhong, L.; Changsheng, W., Characterization of Constitutive Promoters for the Elicitation of Secondary Metabolites in Myxobacteria. *ACS synth. biol.* **2021,** *10*(11), 2904-2909.
