# Supplementary material for: Deciphering the Biosynthesis and Physiological Function of 5-Methylated Pyrazinones Produced by Myxobacteria
Source: ACS Cent Sci. 2024 Feb 7;10(3):555–68. doi: 10.1021/acscentsci.3c01363 (PMC10979478; doi:10.1021/acscentsci.3c01363)
Supplement: Supplementary file 2 — oc3c01363_si_002.pdf [file oc3c01363_si_002.pdf]

oc-2023-01363d.R1

Name: Peer Review Information for "Deciphering The Biosynthesis and Physiological Function of 5-Methylated Pyrazinones Produced by Myxobacteria"

First Round of Reviewer Comments

Reviewer: 1

Comments to the Author

In this manuscript, Zhu et al. report the conmrehensive biochemical study on 5-methylated pyrazinones produced by myxobacteria. The authors first discovered 5-methylated pyrazinone natural products from myxobacteria and its biosynthesis mediated by PSK-NRPS was established by in vitro and in vivo analysis. Interestingly, coralinone was found to exacerbate cellular aggregation of C. exiguus SDU70 and M. xanthus DK1622 by stimulating the secretion of extracellular matrix. It was also suggested that corB is a self-resistance gene encoding a protease to antagonize the agglutination-inducing effect of the natural product. According to the phylogenetic analysis, this mechanism is predicted to be conserved in myxobacteria. The work is comprehensive, and the manuscript is easy to read, and expands our knowledge regarding the chemical ecology of myxobacteria. This reviewer recommends publication in this journal after minor modification.

1. Fig 4, DFT calculation: Transition states in the pathway should be optimized and calculated to determine the activation energies. The provided data are not useful enough to determine the pathway. In addition, it is not clear whether the decarboxylation and oxidation are non-enzymatic.

2. Page 3, “the substrates preference of CorA was in general accordance with in vivo results” The substrate preference of CarA was not studied in vitro.

3. Figure S12: Based on the result of DK1622+1b’-3d, 1b’ was converted to 1, which is not consistent with the text.

4. “13C-labelled S- adenosglmethiomine (SAM)” adenosylmethionine.

5. Page 2, line 50: Gibbs energy

6. Functions of other enzymes in gene cluster is still unclear. The function analysis of ORF2, 3, 7 and 8 should be performed.

Reviewer: 2

Comments to the Author

This manuscript by Zhu et al. reported the biosynthesis of 5-methylated

pyrazinones in a myxobacterial strain Corallococcus exiguus SDU70 and the characterization of their physiological functions. 5-Methylated pyrazinones coralinones A (1) and B (2) were isolated from C. exiguus SDU70 and their structures were fully elucidated, including NMR and X-ray analysis. One NRPS-PKS hybrid CoA was mined from the genome of this strain and functionally validated for the biosynthesis of coralinones A (1) and B (2) by heterologous expression in E. coli and in vitro enzyme reactions. Notably, several new analogs were produced in in vitro reactions. In addition, the C5-Me was derived from the acetate unit installed by a PKS through a decarboxylation reaction. The authors further reported the cellular aggregation of C. exiguus SDU70 and M. xanthus DK1622 induced by coralinones as well as a synthetic intermediate through genetic manipulation and chemical supplementation. Interestingly, only one intermediate Boc-protected dipeptide aldehyde 10b' showed potent inhibition toward cathepsin L. The authors found that coralinone stimulates the secretion of extracellular matrix, which is correlated with cell aggregation. Through the phylogenetic analysis of the coralinone BGCs mined from available myxobacterial genomes, the authors found three clades and more importantly, co-occurred protease CoB. CoB was found to be a secreted protease that can degrade extracellular matrix, serving as a self-resistance mechanism.

Overall, this work is significant in uncovering the biosynthesis of 5-methylated pyrazinones and also their physiological functions. The experiments were well designed and performed. Its quality meets well the standard of this journal. Some minor comments are show below, which should be properly addressed.

(1). From 34L culture, the authors purified 3.8 mg of compound 1 (3.8 mg) and and 7.2 mg of 2, indicating 0.1 to 0.2 mg/L of these compounds. However, chemical complementation experiments (Fig 4) required 5 mg/L compound 1. Please explain the difference or test different concentrations.

(2). Significantly more 2 over 1 was produced in transformed E. coli and in vitro reactions. But cultured C. exiguus SDU70 produced them at a similar level. Please discuss the difference.

(3). Please discuss in detail the decarboxylation reaction shown in Figure 2. Any preceding examples can be cited.

(4). In Figure 7, the sizes of CoB homologs in different BGCs varied. Do they all encode the enzyme of the same family?

(5). Providing statistic information in figure legends as appropriate.

Reviewer: 3

Comments to the Author

The manuscript submitted by Zhu et al. describes the structure, activity, biosynthesis, self-resistance, and phylogenetic distribution of newly discovered pyranzinones from the myxobacterium Corallococcus exiguus SDU70. The authors include data from numerous experiments to support their conclusion that 1) coralinones are 5-methylated pyrazinones (traditional isolation and structural elucidation), 2) produced by an NRPS-PKS cluster (genome mining; heterologous expression; in vitro biocatalysis), and 3)induce agglutination of SDU70 and M. xanthus in liquid cultures (activity assays). The authors also identify a likely self-resistance mechanism that involves a co-clustered proteolytic enzyme CorB and provide evidence that CorB alleviates coralinone-induced aggregation. Overall, this is a thorough investigation of a small signaling molecule produced by a myxobacterium. The associated chemical ecology of myxobacterial small molecules in Nature and potential role in myxobacterial development (and maybe motility) make this extensive effort very interesting to numerous disciplines interested in myxobacteria. The included experiments and resulting data are well done and sufficient for the major conclusions provided by the authors. However, the current version of the manuscript includes many minor errors that negatively impact the quality and breadth of the authors' work. Below are comments/questions for the authors as well as a list of noticed errors to be correct (not exhaustive).

Discussion: The final sentence of paragraph 1 very carefully describes the work as "the first experimentally validated case for small molecule-regulated agglutination of myxobacteria in submerged cultures." I think this is correct as written, but the very specific details and limited context for other small molecules from myxobacteria found to induce aggregation/agglutination (which are the initial steps in fruiting body formation) limit the appeal of the authors' effort. For example, the pheromone stigmalone was found to induce aggregation of Stigmatella aurantiaca in the early 80s (Stephens et al. J Bacteriol 1982). The authors even mention the recent discovery that ambruticin induces early fruiting body formation (which includes aggregation) during competition between M. xanthus and S. cellulosum. What the authors have done with this work that stands out is the complete chemical and biosynthetic characterization, identification of likely self-resistance, mapping of phylogenetic distribution, and discovery of activity for aggregation-inducing metabolites from a myxobacterium. I think some extra context describing known connections between myxobacterial development and small molecule signaling might benefit the reception and impact of the work across disciplines.

Is C. exiguus SDU70 a branched chain amino acid auxotroph? Myxobacteria are known to be BCAA auxotrophs, and this is often associated with their predatory lifestyles i.e. they must acquire BCAAs from lysed prey. If so, your discovery presents an interesting scenario where SDU70 might produce coralinones when local valine/leucine concentrations are high (after prey lysis) to induce rippling or aggregation and ensure thorough acquisition of nutrients.

Do the authors see any connections between coralinones and myxobacterial A-factors? I couldn’t help but draw parallels between the structure(s) and activity of coralinones and candidate A-factors. If the authors are able to confidently reference similarities between them during the discussion, their discovery might penetrate the less chemically-inclined disciplines interested in myxobacteria and chemical ecology.

In the activity results section, the authors mention the active concentration (5 mg/l) of coralinone A mirrors the total production of coralinones in SDU70. However, the reported isolated yield from 36 liters was 11 mg (coralinone A and B combined). Can the authors justify their use of "mirrors" here?

Abstract: “biomemetic” should be biomimetic

Abstract: “featuring with 5-methylated” needs rewording

Abstract: “PKS moiety” is technically correct when referencing the PKS “half” of the hybrid cluster, but there may be confusion with the chemical usage of moiety.

Abstract: Describing cultures as liquid or aqueous instead of “submerged” would be more apt.

Abstract: The self-resistance sentence is confusing as written without context provided by the manuscript. Rewriting the sentence to be more descriptive may improve clarity.

Introduction: Myxobacteria have been assigned to the phyla Myxococotta (or at least proposed to be) and are no longer considered proteobacteria.

Introduction: I’m not sure the peptide bond-forming reaction of pyrazinones should be described as a Mannich reaction. Mannich reactions are C-C bond-forming reactions.

Pg2:column2:line21: SAM is misspelled

Pg2:column2:line29: identification is misspelled

Pg2:column2:line52: First use of E. coli shouldn’t abbreviate the genus

Pg2:column2:line53: PPTase shouldn’t be abbreviated, and it would be helpful to clarify that sfp specifically primes the thiolation/ACP domains of both NRPSs and PKSs.

Figure 3: The included abbreviation “dPKS” is not used.

Page3:column1:line20: “homogenesis” should be corrected to homogenous

Page5:col2:line17: known is misspelled

Figure 7: “ketosynthetase” should be corrected to ketosynthase; “adenyl transferase” should be corrected to acyltransferase; and “thioesteras” should be corrected to thioesterase

Page8:col1:line10: morphology is misspelled

Promoter is misspelled as “promotor” throughout the manuscript

Page8:col2:line3: “questionate” should be corrected to question

Page8:col2:line6: strength is misspelled

Page8:col2:line19: judged is misspelled

Page9:col1:line15: subsequent is misspelled

Methods: Volumes for the cultures used in isotope labeling experiments should be included

Page11:col1:line2: full-length is misspelled

Page11:col1:line39: substituted is misspelled

Page11:col1:line50: codon is misspelled

Page12:col1:line1: images is misspelled

SI: Table S4 has errors when referencing M. xanthus and C. exiguus.

SI: Coralinone B is mislabelled in Figure S8

SI: Figure S9 has included white panels that appear to obfuscate something near the axis

Author's Response to Peer Review Comments:

Response to editor

U EMAIL: Please include the email address of the corresponding author on the first page of the manuscript, and the Supporting Information if submitted, with an asterisk next to their name in the author list. Please be sure to label “email.” >>> done

TOC: Please move to the last page of the manuscript, beneath the References, and label as "TOC Graphic." >>> done

SI PARAGRAPH: If the manuscript is accompanied by any supporting information for publication, a brief description of the supplementary material is required in the manuscript. The appropriate format is: Supporting Information. Brief statement in non-sentence format listing the contents of the material supplied as Supporting Information. >>> ˝SI PARAGRAPH" has been added to the main text.

GENERAL REF FORMATTING: Periodical references should contain authors’ surnames followed by initials, article title, journal abbreviation, year, volume number, and page range. Refs with more than 10 authors should list the first 10 and then be followed by “et al.”

>>>The format of references has been corrected according to the requirement.

Web sources must include access date.

>>>The access date for the downloading the myxobacterial genomic sequences from RefSeq database has been specified.

SYNOPSIS MISSING: The synopsis should be no more than 200 characters (including spaces) and should reasonably correlate with the TOC graphic. The synopsis is intended to explain the importance of the article to a broader readership across the sciences. Please place your synopsis in the manuscript file after the TOC graphic, and label it as "Synopsis."

>>> A short synopsis has been added after TOC graphic.

Reviewer: 1

Comments:

In this manuscript, Zhu et al. report the conmrehensive biochemical study on 5methylated pyrazinones produced by myxobacteria. The authors first discovered 5methylated pyrazinone natural products from myxobacteria and its biosynthesis mediated by PSK-NRPS was established by in vitro and in vivo analysis. Interestingly, coralinone was found to exacerbate cellular aggregation of C. exiguus SDU70 and M. xanthus DK1622 by stimulating the secretion of extracellular matrix. It was also suggested that corB is a self-resistance gene encoding a protease to antagonize the agglutinationinducing effect of the natural product. According to the phylogenetic analysis, this mechanism is predicted to be conserved in myxobacteria. The work is comprehensive, and the manuscript is easy to read, and expands our knowledge regarding the chemical ecology of myxobacteria. This reviewer recommends publication in this journal after minor modification.

>>>It is highly appreciated for the positive words to our work.

1. Fig 4, DFT calculation: Transition states in the pathway should be optimized and calculated to determine the activation energies. The provided data are not useful enough to determine the pathway. In addition, it is not clear whether the decarboxylation and oxidation are non-enzymatic.

>>>We delete the DFT calculation (Figure S4) in the revised supporting information, since it does not help improve the quality of the manuscript, but just cause confusion thereby.

Both the biomimetic total synthesis (supporting information) and *in vitro* biochemical reaction (Figure 3) confirmed that the decarboxylation and oxidation do not require any additional enzymatic catalysts. In organic chemistry, β-keto decarboxylation is a quite easily-happening reaction without the need for catalyst (see https://www.integratedmcat.com/organic-mechanisms/Decarboxylation-of-a-Beta-Keto-Acid/030209_n040). And also, the non-enzymatic reaction of the oxidation is commonly accepted for the formation of pyrazinone backbone, as you can find in our cited references 8–11, 21, 29 in the manuscript.

In the revised manuscript, we briefly discuss the possibility of the two pathways, as following:

"we postulated that Route I is more favorable, because β-keto acid loses carbon dioxide quite easily, where the immediate product will be a resonant stabilized enolate anion followed by tautomerism into ketone.[24] "

2. Page 3, “the substrates preference of CorA was in general accordance with in vivo results” The substrate preference of CorA was not studied in vitro.

>>>In the original version of our manuscript, we have actually probed on the substrate preference of CorA using the pairwise combination assay *in vitro*, and the data was provided in Figure S6 (now Figure S5). In Figure S7, we further tested the substrate preference of the first A domain in CorA using the standard ATP–PPi exchange assay, and the obtained results were in agreement with the pairwise combination assay. Regretfully, we have to compromise on the second A domain because we failed to obtain the soluble protein despite of exhaustive efforts. The experimental procedure for the ATP–PPi exchange assay has been added into the Method section of the revised manuscript.

3. Figure S12: Based on the result of DK1622+1b’-3d, 1b’ was converted to 1, which is not consistent with the text.

>>>Thanks for the scrutiny. According to the UV spectrum, the peak in the HPLC chromatogram DK1622+1b’-3d is not compound **1**. It is just an anonymous compound with similar retention time to **1**. Therefore, the figure and the text as described are consistent.

4. “13C-labelled S- adenosglmethiomine (SAM)” adenosylmethionine. >>> revised

5. Page 2, line 50: Gibbs energy >>> revised

6. Functions of other enzymes in gene cluster is still unclear. The function analysis of ORF2, 3, 7 and 8 should be performed.

>>>The putative functions of ORF2, 3, 7 and 8 have been bioinformatically predicted and summarized in Table S1. The experimental validation of their putative functions will require many more tedious genetic and biochemical work, which should be published in a separate paper.

Reviewer: 2

Comments:

This manuscript by Zhu et al. reported the biosynthesis of 5-methylated

pyrazinones in a myxobacterial strain Corallococcus exiguus SDU70 and the characterization of their physiological functions. 5-Methylated pyrazinones coralinones A

(1) and B (2) were isolated from C. exiguus SDU70 and their structures were fully elucidated, including NMR and X-ray analysis. One NRPS-PKS hybrid CoA was mined from the genome of this strain and functionally validated for the biosynthesis of coralinones A (1) and B (2) by heterologous expression in E. coli and in vitro enzyme reactions. Notably, several new analogs were produced in in vitro reactions. In addition, the C5-Me was derived from the acetate unit installed by a PKS through a decarboxylation reaction. The authors further reported the cellular aggregation of C.

exiguus SDU70 and M. xanthus DK1622 induced by coralinones as well as a synthetic intermediate through genetic manipulation and chemical supplementation. Interestingly, only one intermediate Boc-protected dipeptide aldehyde 10b' showed potent inhibition toward cathepsin L. The authors found that coralinone stimulates the secretion of extracellular matrix, which is correlated with cell aggregation. Through the phylogenetic analysis of the coralinone BGCs mined from available myxobacterial genomes, the authors found three clades and more importantly, co-occurred protease CoB. CoB was found to be a secreted protease that can degrade extracellular matrix, serving as a selfresistance mechanism.

Overall, this work is significant in uncovering the biosynthesis of 5-methylated pyrazinones and also their physiological functions. The experiments were well designed and performed. Its quality meets well the standard of this journal. Some minor comments are show below, which should be properly addressed. >>>Many thanks for the acceptance to our manuscript.

(1). From 34L culture, the authors purified 3.8 mg of compound 1 (3.8 mg) and and 7.2 mg of 2, indicating 0.1 to 0.2 mg/L of these compounds. However, chemical complementation experiments (Fig 4) required 5 mg/L compound 1. Please explain the difference or test different concentrations.

>>> As well known to natural product chemists, the isolated yield is always smaller than actual yield by the microbial producers, because there is an inevitably considerable loss during the extraction and isolation process. We used the purified coralinones as chemical standard to roughly quantify the compounds **1** and **2** in the HP20 resin-resolved crude extract of SDU70 fermentation broth (Figure S1), which turned out to be ~1.5 mg/L. We reasoned that actual total yield of coralinones by SDU70 should be more than 1.5 mg/L, because (1) the coralinones retained intracellular escaped from the calculation; (2) potential minor congeners such as compounds **3**–**6** masked in the HPLC profile were neither taken into account. In other words, coralinones are definitely produced at mg scale by SDU70. As you can find in Figure S9, 2 mg/L actually started to cause visible agglutination, although it was not as severe as 5 mg/L. In this sense, the tested potency of purified **1** is reasonable. To be more explicit, we modified the two sentences related to the potency of **1** on the page 4 of the revised manuscript, as following:

> "*The pellet morphogenesis of SDU70-△corA was significantly impeded compared with SDU70-wt and SDU70-corA, which could be restored by the chemical complementation of **1** at 5 mg/L (Figure 4B), a concentration mirroring the total production level (~1.5 mg/L) of coralinones given by SDU70-wt (Figure S1)* ".
>
> *"In addition, **1** caused the flocculation of SDU70-△corA and DK1622-wt in a dosedependent manner, whereby 2 mg/L started to be active, and 5 mg/L caused severe flocculation (Figure S10)".*

(2). Significantly more 2 over 1 was produced in transformed E. coli and in vitro reactions. But cultured C. exiguus SDU70 produced them at a similar level. Please discuss the difference.

>>> The regulation of NPs biosynthesis in microbes is very complex and might be affected by many factors. We speculate that the difference in the relative abundance of **1** and **2** might be attributed to the difference in the precursor supply and/or CorA activity in *E. coli* and *C. exiguus* SDU70. Extrapolated from the question/comment 2 of the reviewer 3, myxobacteria have a distinct metabolism compared with E. coli, which might also contribute to the different production of coralinone.

We add a brief discussion in the page 3 of the revised manuscript, as following:

"*Noteworthy, the inconsistency in the relative abundance of **1** and **2** given by the recombinant E. coli BAP1 and native C. exiguus SDU70 (Figure S1) implicated the substrate selectivity of CorA and/or precursor supply is different in these two hosts*"

(3). Please discuss in detail the decarboxylation reaction shown in Figure 2. Any preceding examples can be cited.

>>>In organic chemistry, β-keto decarboxylation is an easily-happening reaction without the need for catalyst. Actually, both biomimetic total synthesis work (see the supporting information file) and biochemical reactions (Figure 3) also unambiguously corroborated the spontaneous decarboxylation. In the page 2 of the revised manuscript, we discuss the decarboxylation and cite a review paper with this respect, as following:

"we postulated that Route I is more favorable, because β-keto acid loses carbon dioxide quite easily, where the immediate product will be a resonant stabilized enolate anion followed by tautomerism into ketone.[24] "

(4). In Figure 7, the sizes of CoB homologs in different BGCs varied. Do they all encode the enzyme of the same family?

>>> We have manually checked peptidases in Figure 7. Interestingly, peptidase exists in Clade I and III, but not in Clade II. The sizes of CorB homologs in Clade I and III are different. In Clade I, the CorB homologues belong to the Do protease family (*J Biochem Mol Biol.* 2005, 38, 266-74; *Nature* 2008, 453, 885–890.), with the prominent member is DegP that we have extensively described in the main manuscript. Instead in Clade III, the CorB homologues are belonging to the DPP11 subtype of S46 family (*Sci Rep*. 2019, 9, 13587; *Biochimie*, 2018, 147, 25-35).

(5). Providing statistic information in figure legends as appropriate.

>>> Statistic information was added into the figure legends where needed. Basically, each experiment was done in triplicate, and error bars correspond to standard deviation.

Reviewer: 3

Comments:

The manuscript submitted by Zhu et al. describes the structure, activity, biosynthesis, self-resistance, and phylogenetic distribution of newly discovered pyranzinones from the myxobacterium Corallococcus exiguus SDU70. The authors include data from numerous experiments to support their conclusion that 1) coralinones are 5-methylated pyrazinones (traditional isolation and structural elucidation), 2) produced by an NRPS-PKS cluster (genome mining; heterologous expression; in vitro biocatalysis), and 3) induce agglutination of SDU70 and M. xanthus in liquid cultures (activity assays). The authors also identify a likely self-resistance mechanism that involves a co-clustered proteolytic enzyme CorB and provide evidence that CorB alleviates coralinone-induced aggregation. Overall, this is a thorough investigation of a small

signaling molecule produced by a myxobacterium. The associated chemical ecology of myxobacterial small molecules in Nature and potential role in myxobacterial development (and maybe motility) make this extensive effort very interesting to numerous disciplines interested in myxobacteria. The included experiments and resulting data are well done and sufficient for the major conclusions provided by the authors. However, the current version of the manuscript includes many minor errors that negatively impact the quality and breadth of the authors' work. Below are comments/questions for the authors as well as a list of noticed errors to be correct (not exhaustive).

>>>It is grateful for the positive comments and valuable suggestions. We perceive the reviewer as a mentor to our work.

(1) Discussion: The final sentence of paragraph 1 very carefully describes the work as "the first experimentally validated case for small molecule-regulated agglutination of myxobacteria in submerged cultures." I think this is correct as written, but the very specific details and limited context for other small molecules from myxobacteria found to induce aggregation/agglutination (which are the initial steps in fruiting body formation) limit the appeal of the authors' effort. For example, the pheromone stigmalone was found to induce aggregation of Stigmatella aurantiaca in the early 80s (Stephens et al. J Bacteriol 1982). The authors even mention the recent discovery that ambruticin induces early fruiting body formation (which includes aggregation) during competition between M. xanthus and S. cellulosum. What the authors have done with this work that stands out is the complete chemical and biosynthetic characterization, identification of likely selfresistance, mapping of phylogenetic distribution, and discovery of activity for aggregation-inducing metabolites from a myxobacterium. I think some extra context describing known connections between myxobacterial development and small molecule signaling might benefit the reception and impact of the work across disciplines. >>>Many thanks for this excellent suggestion. Accordingly, we added more discussions to attract attentions from a broader range of scientific communities, as following:

> "*The lipid stigmalone produced by Stigmatella aurantiaca functionates as a pheromone that induces the cellular aggregation and enhances fruiting body formation.*[42]*" Romanowski and co-workers recently identified the lipodepsipeptide selethramide that promotes motility of its native producer belonging to Gram-negative Burkholderia, a bacterial family sharing considerable physiological similarities to myxobacteria.*[44] *These examples strongly support a close connection between developmental growth and sophisticated small molecules deliberately encoded by myxobacteria. It is conceivable that many more alike cases remain to be unveiled, which will provide a new avenue to investigate the sociobiology of myxobacteria. From another perspective, it might be particularly fruitful to exploit the ecology-based strategies to unlock the silent BGCs for continued natural products discovery from myxobacteria, given their distinct life patterns (social behavior, predation, swarming, etc).*
> .

(2) Is C. exiguus SDU70 a branched chain amino acid auxotroph? Myxobacteria are known to be BCAA auxotrophs, and this is often associated with their predatory lifestyles i.e. they must acquire BCAAs from lysed prey. If so, your discovery presents an interesting scenario where

SDU70 might produce coralinones when local valine/leucine concentrations are high (after prey lysis) to induce rippling or aggregation and ensure thorough acquisition of nutrients.

>>> We sincerely appreciate for the guidance! The aim of this study is to elucidate the biogenesis of 5-methylated pyrazinones in myxobacteria, and reveal their physiological roles in modulating cellular aggregation. We think "auxotrophy and/or predation" are out the scope of this manuscript at this moment. However, your comment indeed positively inspires us to further carry out another paper, to systematically investigate if coralinones are associated with other physiological processes of myxobacteria, including but not limited to predation, biofilm formation, rippling, etc. In addition, efforts are still underway in our laboratory to identify the binding targets of coralinones through transcriptomics and proteomics.

(3) Do the authors see any connections between coralinones and myxobacterial Afactors? I couldn't help but draw parallels between the structure(s) and activity of coralinones and candidate A-factors. If the authors are able to confidently reference similarities between them during the discussion, their discovery might penetrate the less chemically-inclined disciplines interested in myxobacteria and chemical ecology.

>>> Currently, we are not sure if there is any relationship between coralinones and myxobacterial A-factors or C-factor, although it would be interesting to investigate this aspect in the future. We do mention this point in the discussion section of the revised manuscript (page 9) to attract attention of scientists from multiple disciplines, as following:

> *"For myxobacteria, cellular aggregation has been previously ascribed to peptidic substances like A-factors[48,49] and/or C-factor.[50] The involvement of small-molecule morphogens for coordinating this physiological process is surprising to us. It's yet unclear if mechanistic connections exist for the signaling pathways of coralinone and A-factors or C-factor."*

(4) In the activity results section, the authors mention the active concentration (5 mg/l) of coralinone A mirrors the total production of coralinones in SDU70. However, the reported isolated yield from 36 liters was 11 mg (coralinone A and B combined). Can the authors justify their use of "mirrors" here?

>>>Please kindly refer to the answer to the question 1 of the reviewer 2.

Abstract: "biomemetic" should be biomimetic

>>> revised

Abstract: "featuring with 5-methylated" needs rewording

>>> "coralinone featuring with 5-methylated pyrazinone backbone" was changed into "the 5-methylated pyrazinone coralinone".

Abstract: "PKS moiety" is technically correct when referencing the PKS "half" of the hybrid cluster, but there may be confusion with the chemical usage of moiety. >>> "PKS moiety" was changed into "PKS part".

Abstract: Describing cultures as liquid or aqueous instead of "submerged" would be more apt.

>>> We have described all the words associated with "submerged" into "liquid" throughout this manuscript.

Abstract: The self-resistance sentence is confusing as written without context provided by the manuscript. Rewriting the sentence to be more descriptive may improve clarity.

>>> we have modified this sentence:

"*This phylogenetic contextualization provoked us to identify corB located in the proximity of corA as a self-resistance gene. CorB was experimentally verified to be a protease that hydrolyzes extracellular proteins to antagonize the agglutinationinducing effect of coralinone.*"

Introduction: Myxobacteria have been assigned to the phyla Myxococotta (or at least proposed to be) and are no longer considered proteobacteria. >>> revised.

Introduction: I'm not sure the peptide bond-forming reaction of pyrazinones should be described as a Mannich reaction. Mannich reactions are C-C bond-forming reactions.

>>> Yes, strictly speaking, Mannich reaction is a multi-component reaction (see https://www.organic-chemistry.org/namedreactions/mannich-reaction.shtm). Mannich reaction mechanism begins with the formation of an iminium ion from the reaction between formaldehyde and amine (C-N bond formation), followed by C-C formation. In other words, Mannich reaction firstly undergoes C-N condensation. To avoid confusion, we still delete the wording "Mannich reaction" in the introduction section.

Pg2: column2: line 21: SAM is misspelled >>> the typo was corrected.

Pg2: column2: line29: identification is misspelled >>> the typo was corrected.

Pg2: column2: line52: First use of E. coli shouldn't abbreviate the genus

>>> *E. coli* was changed into *Escherichia coli*

Pg2: column2: line53: PPTase shouldn't be abbreviated, and it would be helpful to clarify that sfp specifically primes the thiolation/ACP domains of both NRPSs and PKSs.

>>> We have replaced the "PPTase" with "phosphopantetheinyl transferases". And also, we reworded the sfp priming, as following:

"a strain engineered with sfp gene that encodes a promiscuous phosphopantetheinyl transferase for the heterologous priming of A and AT domains of both NRPSs and PKSs"

Figure 3: The included abbreviation "dPKS" is not used.

>>> In Figure 3, we have replaced "dCorA-PKS" with "dPKS".

Page3: column1: line20: "homogenesis" should be corrected to homogenous >>> the typo was corrected

Page5: col2： line17: known is misspelled >>> the typo was corrected

Figure 7: “ketosynthetase” should be corrected to ketosynthase; “adenyl transferase” should be corrected to acyltransferase; and “thioesteras” should be corrected to thioesterase >>> revised

Page8: col1: line10: morphology is misspelled

>>> the typo was corrected

Promoter is misspelled as “promotor” throughout the manuscript >>> the typo was corrected

Page8: col2: line3: “questionate” should be corrected to question >>> the typo was corrected

Page8: col2: line6: strength is misspelled >>> the typo was corrected

Page8: col2: line19: judged is misspelled >>> the typo was corrected

Page9: col1: line15: subsequent is misspelled

>>> the typo was corrected

Methods: Volumes for the cultures used in isotope labeling experiments should be included

>>> The sentence: "*SDU70 was transferred to 100 mL liquid medium and grown for 36 h*", has been added.

Page11: col1: line2: full-length is misspelled >>> the typo was corrected

Page11: col1: line39: substituted is misspelled >>> the typo was corrected

Page11: col1: line50: codon is misspelled >>> the typo was corrected

Page12: col1: line1: images is misspelled >>> the typo was corrected

SI: Table S4 has errors when referencing M. xanthus and C. exiguus. >>> The errors in Table S4 have been corrected.

SI: Coralinone B is mislabelled in Figure S8

>>> Corrected

SI: Figure S9 has included white panels that appear to obfuscate something near the axis >>> we have changed the correct graph.
